# Synthesis and In Vitro Evaluation of the Anticancer Effect of Novel Phosphonium Vindoline Derivatives

**DOI:** 10.3390/ijms26083775

**Published:** 2025-04-16

**Authors:** Mónika Halmai, Viktória Donkó-Tóth, Péter Keglevich, Károly Kánai, Márton Weber, Miklós Dékány, Ejlal A. Abdallah, Noémi Bózsity, István Zupkó, Andrea Nehr-Majoros, Éva Szőke, Zsuzsanna Helyes, László Hazai

**Affiliations:** 1 Department of Organic Chemistry and Technology, Faculty of Chemical Technology and Biotechnology, Budapest University of Technology and Economics, Műegyetem rkp. 3, H-1111 Budapest, Hungary; 2 Spectroscopic Research Department, Gedeon Richter Plc., Gyömrői út 19-21, H-1103 Budapest, Hungary; 3 Institute of Pharmacodynamics and Biopharmacy, University of Szeged, Eötvös u. 6, H-6720 Szeged, Hungary; 4 Department of Pharmacology and Pharmacotherapy, Medical School & Centre for Neuroscience, University of Pécs, Szigeti út 12, H-7624 Pécs, Hungary; 5 National Laboratory for Drug Research and Development, Magyar Tudósok krt. 2, H-1117 Budapest, Hungary; 6 HUN-REN PTE Chronic Pain Research Group, Szigeti út 12, H-7624 Pécs, Hungary; 7 PharmInVivo Ltd., Szondi Gy. u. 10, H-7629 Pécs, Hungary

**Keywords:** triphenylphosphine, vindoline, phosphonium salts, anticancer effect, cell viability

## Abstract

The *Vinca* alkaloid vindoline was coupled at position 17 with several trisubstituted phosphine derivatives and their in vitro anticancer activities on 60 human tumor cell lines (NCI60) were investigated. This phosphonium-type ionic side chain is beneficial because it allows therapeutic molecules to pass through the cell membrane. Thus, the candidates coupled to it can exert their activities in the mitochondria. The coupling of vindoline with the trisubstituted phosphines was achieved through flexible or rigid linkers. Instead of the ionic phosphonium structural part, a neutral moiety, namely the triphenylmethyl group, was also added to the side chain, being sterically similar but without a charge and phosphorus atom. In addition, the triphenylphosphine element was also built at position 10 of vindoline. Most of the derivatives showed low micromolar growth inhibition (*GI*_50_) values against most cell lines. Among them, conjugate **9e** was outstanding: it exhibited nanomolar anticancer activity on the RPMI-8226 leukemia cell line (*GI*_50_ = 20.0 nM). Compound **9g** elicited cell cycle disturbance and apoptosis on A2780 ovary cancer cells and inhibited their migration at subantiproliferative concentrations. The selectivity of the conjugates was determined by their effects on non-tumor Chinese hamster ovary (CHO) cells in the CellTiter-Glo Luminescent Cell Viability Assay. Compound **9e** showed an estimated half-maximal inhibitory concentration (*IC*_50_) value of 1.36 µM, suggesting good selectivity on cancer cells. These results open new perspectives of novel phosphonium-based vindoline derivatives as anticancer compounds.

## 1. Introduction

The range of biologically active compounds containing phosphorus is considerably wide [1,2,3,4,5,6]. In addition to chemical weapons and pesticides, many organic phosphorus compounds are also found in the field of medicine [7,8,9,10]. These include bisphosphonates or dronates, which are used in bone-related disorders, e.g., in cases of osteoporosis [11,12,13]. In other parts of the clinic, such as in anticancer chemotherapy, organophosphorus compounds are also used [14,15,16,17,18,19].

**Figure 1:**
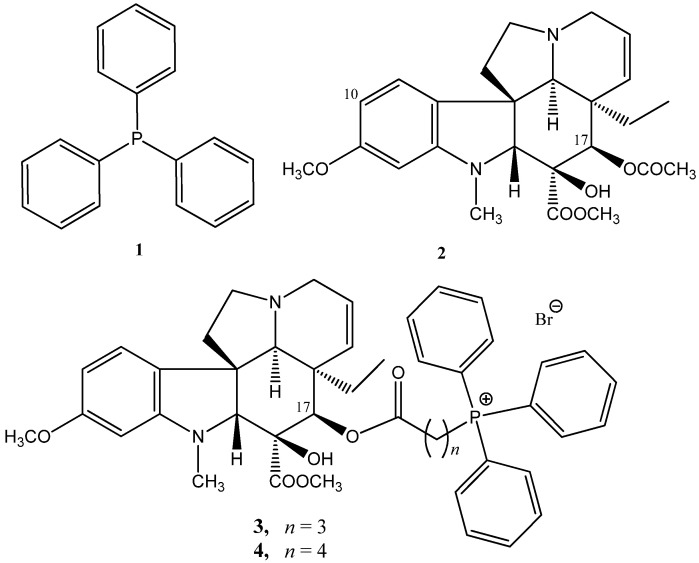
The structure of triphenylphosphine (**1**), vindoline (**2**), and previously synthesized vindoline–TPP conjugates (**3**, **4**).

One of the most exciting derivatives in this area is triphenylphosphine (**1**, TPP) (Figure 1), which, due to its lipophilic character, can improve cell membrane permeability as part of a hybrid and promote the accumulation of drugs inside the cell, especially in the mitochondria [20,21,22,23,24]. Mitochondria are cell organelles with a double membrane system which perform several tasks: oxidative phosphorylation–ATP biosynthesis, regulation of the ion concentration of the cytoplasm, maintenance of Ca^2+^ homeostasis, participation in signaling processes, and, where appropriate, activation of apoptosis. Insufficiently functioning mitochondria begin to release free radicals (ROS compounds) that can damage the components of the cell, including DNA. Oxidative stress damages the mitochondria, resulting in malfunction inside the cell because the cell cannot divide or maintain itself due to lack of energy, and apoptosis happens [25].

The rate of intracellular transport of TPP^+^ is 107–108 times higher than that of hydrophilic cations (e.g., Na^+^) [26]. Due to the new structure (in a hybrid), the obtained derivatives will not or will be less likely to be substrates for P-gp, eliminating the phenomenon of resistance. Moreover, a related publication revealed that the TPP unit itself has a cytotoxic effect [27]. The TPP cation (TPP^+^) is one of the best-characterized lipophilic cations, which can transport, for example, active substances with antioxidant properties into the mitochondria and thus prevent diseases arising from mitochondrial stress [28]. Compared to other cell organelles, mitochondria have a high negative transmembrane potential (ΔΨ_m_ approximately 140–180 mV) [29]. In addition, the transmembrane potential of the mitochondria of tumor cells is much higher than that of normal cells. This provides an opportunity for the selective mitochondrial accumulation of positively charged active substances or active substance conjugates. Currently, the most efficient way to deliver drugs to specific mitochondria is the covalent coupling of the selected pharmacophore with a lipophilic cation [30,31].

Some triterpenoid–TPP conjugates are reported to exert significantly increased cytotoxic effects against murine and human cancer cells than the parent triterpenoid [32]. In addition, several anticancer hybrid molecules of this type are known in the literature [33,34]. Moreover, in our research group, TPP was previously coupled to vindoline (**2**), thereby producing hybrids **3** and **4** (Figure 1), which showed significant antitumor activity primarily in cases of breast cancer, colon cancer, and melanoma [35,36].

Vindoline (**2**), a natural organic molecule and one of the components of vinblastine, which is well-known and widely used in anticancer therapy, has already established its importance in many hybrid molecules [37,38,39,40,41,42].

**Figure 2:**
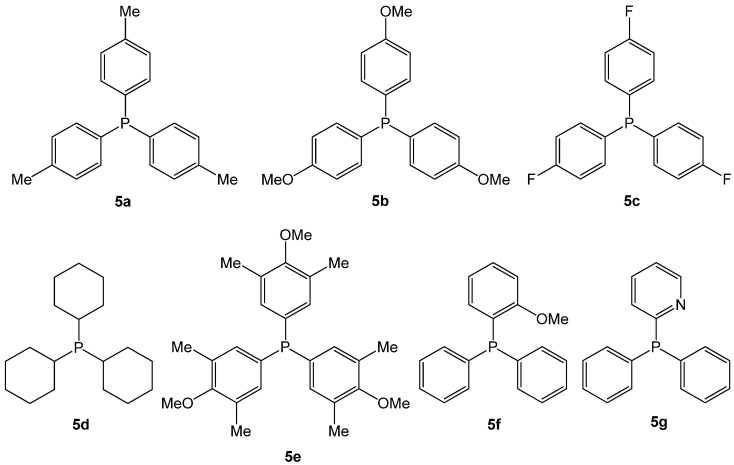
The structure of the selected trisubstituted phosphines (**5a**–**g**).

Based on the notable anticancer effects obtained previously [35,36], we aimed to combine vindoline with differently substituted phosphines. It was presumed that even the ineffective vindoline may have a valuable anticancer effect when attached to trisubstituted phosphines. We planned to perform the conjugation of vindoline at two positions (10 and 17). Two flexible linkers of different lengths and a rigid one were chosen to connect the phosphine and vindoline units. In this preliminary study, five differently substituted, readily available triphenylphosphine derivatives were chosen: tri(*p*-tolyl)phosphine (**5a**), tris(4-methoxyphenyl)phosphine (**5b**), tris(4-fluorophenyl)phosphine (**5c**), tris(4-methoxy-3,5-dimethylphenyl)phosphine (**5e**), and diphenyl(2-methoxyphenyl)phosphine (**5f**) (Figure 2). In addition, the coupling was also achieved with tricyclohexylphosphine (**5d**), containing non-aromatic rings, as well as diphenyl-2-pyridylphosphine (**5g**), containing a nitrogen atom in one of the phenyl groups. For comparison, we also aimed to attach the triphenylmethyl group, which does not contain a phosphorus atom but is sterically similar. Finally, we planned to evaluate the antiproliferative effect of the conjugates and investigate the selectivity with non-tumor cells.

## 2. Results and Discussion

### 2.1. Chemistry

#### 2.1.1. Coupling Trisubstituted Phosphines at Position 17 of Vindoline

**Scheme 1:**
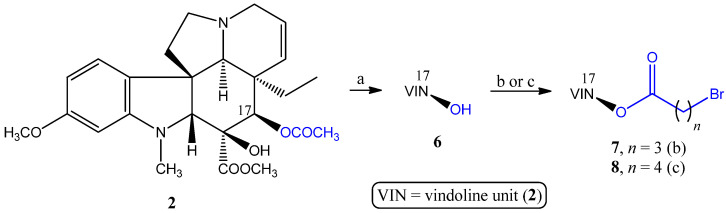
The formation of the linker at position 17 of vindoline (**2**): (a) Na_2_CO_3_, MeOH, reflux, 24 h, 99%; (b) 4-bromobutyric acid, DCC, DMAP, abs. DCM, rt, 4 h, 75%; (c) 5-bromovaleric acid, DCC, DMAP, abs. DCM, rt, 19 h, 69%.

The first goal was to connect the selected seven trisubstituted phosphines (**5a**–**g**) to vindoline (**2**) via position 17. First, a 4- and a 5-carbon flexible linker was built in two steps (Scheme 1). The synthesis of the linker-containing vindoline derivatives (**7** and **8**) was previously elaborated by us [36,42].

**Scheme 2:**
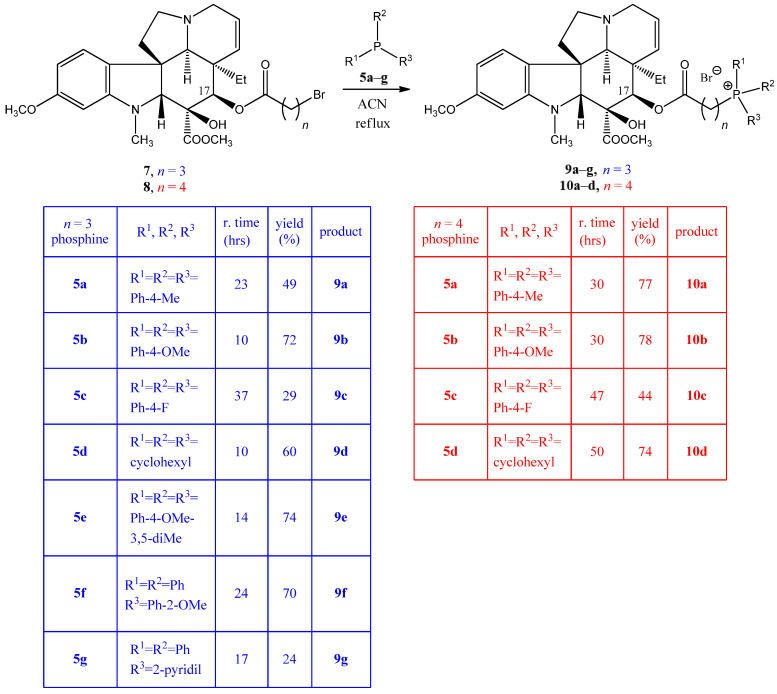
Synthesis of vindoline–phosphine conjugates (**9a**–**g** and **10a**–**d**) coupled via a 4- and a 5-carbon flexible linker at position 17 of vindoline.

In the first round, the conjugation with vindoline derivatives **7** and **8** was carried out with phosphines **5a**–**d.** Then, based on the initial biological results (the length of the linker had a negligible influence on the activity), in the case of the other three phosphines (**5e**–**g**), only **7** was used for the couplings. The **7** 17-bromoacylvindoline was reacted with the corresponding phosphine (**5a**–**g**) (Figure 2), while the **8** analog was reacted with phosphines **5a–d** in an acetonitrile solution under reflux (Scheme 2) in *P*-alkylation reactions, resulting in the vindoline–phosphine conjugates **9a**–**g** and **10a**–**d** with medium to good yields.

#### 2.1.2. Synthesis of a Conjugate via a More Rigid Linker

**Scheme 3:**
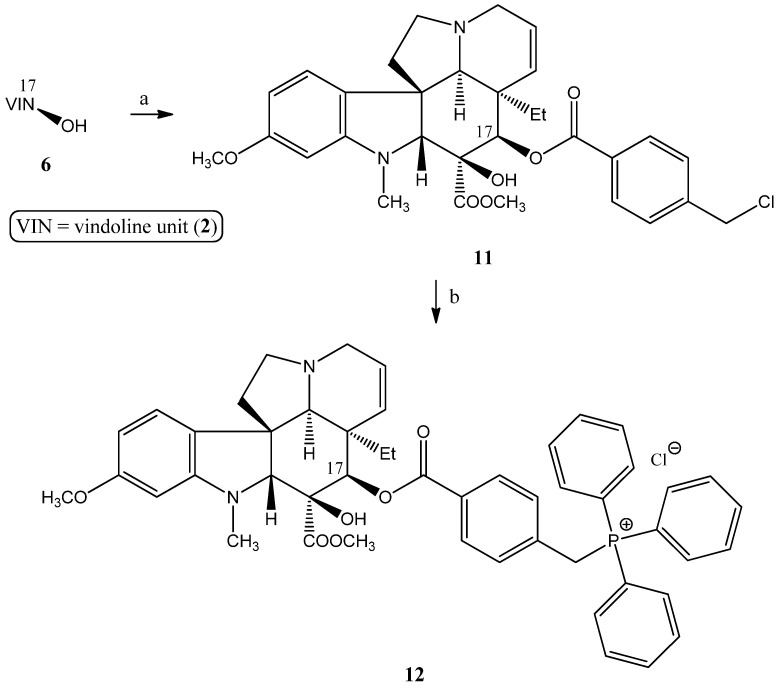
Synthesis of vindoline–TPP conjugate (**12**) coupled via a more rigid linker: (a) 4-(bromomethyl)benzoic acid, SOCl_2_, NEt_3_, abs. DCM, rt, 2 h, 28%; (b) triphenylphosphine (**1**), abs. ACN, reflux, 11 h, 43%.

The next aim was to couple vindoline and TPP (**1**) via a more rigid linker. As a relatively rigid linker, a side chain containing a benzene ring was planned to be formed on the vindoline (**2**). For this purpose, 17-desacetylvindoline (**6**) was *O*-acylated with 4-(bromomethyl)benzoic acid (Scheme 3). The reaction was carried out with thionyl chloride in the presence of triethyl amine in abs. dichloromethane. Under such reaction conditions, the correspondent acid chloride took part in the reaction and, at the same time, the bromine atom in the active benzyl position was replaced by chlorine due to the excess of thionyl chloride. The *P*-alkylation reaction between the chlorovindoline derivative **11** and the TPP (**1**)-afforded conjugate **12**.

#### 2.1.3. Formation of Triphenylmethyl Group at Position 17 of Vindoline

**Scheme 4:**
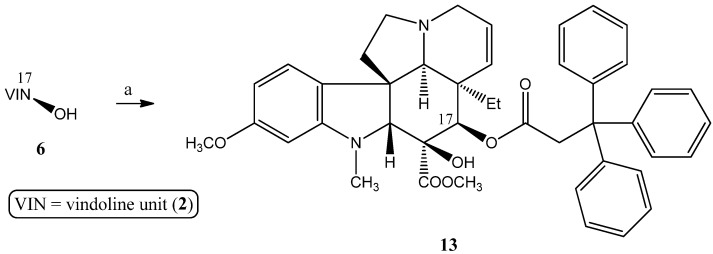
Synthesis of **13** derivative containing triphenylmethyl group at position 17 of vindoline: (a) 3,3,3-triphenylpropionic acid, SOCl_2_, abs. DCM, DMF, 55 °C, 3 h; then 17-desacetylvindoline (**6**), NEt_3_, abs. DCM, reflux, 1 h, 36%.

An interesting comparison can be made via the fact that instead of the ionic trisubstituted phosphine structural part, a neutral moiety was added to the side chain, namely a triphenylmethyl group, which is sterically similar but does not carry an ionic charge. Accordingly, 17-desacetylvindoline (**6**) was *O*-acylated with 3,3,3-triphenylpropanoyl chloride prepared in situ from the corresponding carboxylic acid (Scheme 4). The coupling reaction was performed in abs. dichloromethane in the presence of triethyl amine, and thus the ester **13** with similar space-filling was obtained.

#### 2.1.4. Coupling Triphenylphosphine at Position 10 of Vindoline

**Scheme 5:**
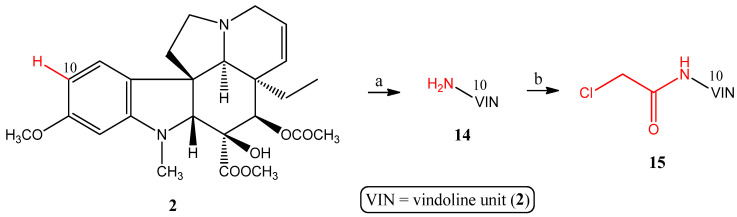
The formation of the linker at position 10 of vindoline (**2**): (a) (1) THF, 1M HCl, NaNO_2_, 0 °C, 30 min, (2) NaBH_4_, MeOH, Ar, rt, 4 h, 92%; (b) chloroacetyl chloride, abs. DCM, K_2_CO_3_, Ar, rt, 7 h, 64%.

Finally, producing a conjugate containing TPP at position 10 of vindoline was also set as a goal, which is another crucial structural modification option. The key intermediate 10-chloroacetamidovindoline (**15**) containing the linker was prepared using the method we developed earlier [40] (Scheme 5).

**Scheme 6:**
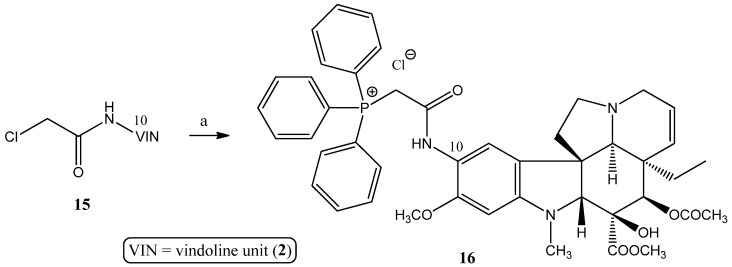
Synthesis of vindoline–triphenylphosphine conjugate **16** coupled via position 10 of vindoline: (a) triphenylphosphine (**1**), abs. ACN, reflux, 26 h, 45%.

The coupling of compound **15** with TPP (**1**) was achieved by the usual procedure in an acetonitrile solution, resulting in derivative **16** being substituted in position 10 (Scheme 6).

### 2.2. Evaluation of the Biological Activities

#### 2.2.1. NCI60 Screening

The in vitro antiproliferative activities of the synthesized compounds (**2**–**4**, **9a**–**f**, **10a**–**d**, and **12**) were examined against 60 human tumor cell lines (NCI60), representing leukemia, non-small cell lung, colon, CNS, ovarian, renal, prostate, and breast cancers, as well as melanoma, at the National Cancer Institute (NCI, USA) according to the standard protocols [43,44,45,46,47,48].

The screening results are demonstrated in the Supplementary Materials (Table S1 and Table S2), where the biological activities were determined for the 10 µM concentration. The percentages of growths show the amount of living cancer cells compared to a reference. The negative numbers indicate significant decreases in the cell number. In our previous work, we have shown that, as expected, vindoline (**2**), 17-deacetylvindoline (**6**), and the linker containing bromovindoline derivative **7** do not exert antiproliferative effects [42].

The experimental data obtained for the novel compounds **9a**–**f** containing 4-carbon linker (**1**) are presented in Table S1. The activity of vindoline (**2**) and the previously prepared phosphonium hybrid **3** containing TPP was also presented as a reference. Except for **9c** trifluoro derivative, all substituted hybrids showed more significant antiproliferative effects than the **3** parent molecule (**3**) containing an unsubstituted TPP unit. The hybrids containing tri(*p*-tolyl) (**9a**)-, tris(4-methoxyphenyl) (**9b**)-, and tris(4-methoxy-3,5-dimethylphenyl) (**9e**)-phosphine building blocks were outstanding, and a more than 70% average reduction was obtained in all three cases. Among them, derivative **9e** was the most potent, which showed an almost 100% cell-killing effect on most tested cell lines (average: −97.13%).

The growth percentages of compounds **10a**–**d**, containing a 5-carbon linker, and **12**, containing a more rigid linker, are summarized in Table S2. The effect of the earlier synthesized phosphonium derivative (**4**) containing TPP was also shown as a reference. Similarly to analogs containing a 4-carbon linker, except for trifluoro derivative **10c**, all substituted hybrids exerted more remarkable antiproliferative activity than the reference **4** containing an unsubstituted TPP part. The hybrids containing tri(p-tolyl) (**10a**)- and tris(4-methoxyphenyl) (**10b**)-phosphine structural elements were notable again, as a more than 70% average cell death was detected in both cases. Derivative **12**, containing a relatively rigid linker, had moderate cytotoxicity.

Since all novel compounds showed observable antiproliferative effects on several cancer cell lines during the one-dose test, they were subjected to a five-dose screening. The 50% growth inhibition (*GI*_50_) and their mean values are demonstrated in Table 1 and Table 2. Regarding mean *GI*_50_ values, derivatives **9a**, **9b**, **9e**, and **9f** and compounds **10a** and **10b** exceeded the corresponding reference (**3** or **4**), all showing mean *GI*_50_ values below 3.0 µM. Derivatives **9a** and **9b**, which contain a 4-carbon linker, were slightly more potent than the **10a** and **10b** analogs, which contain a linker that is one carbon longer. As expected from the one-dose studies, derivative **9e** was the most potent candidate, with a mean *GI*_50_ value of 0.309 µM. The most significant activity was shown by compound **9e** on the RPMI-8226 leukemia cell line (*GI*_50_ = 20.00 nM), but values below 100 nM were also obtained on the leukemia HL-60(TB), colon cancer COLO-205 and KM12, CNS cancer SNB-19 and U251, melanoma SK-MEL-2 and UACC-257, and breast cancer MCF7 and MDA-MB-468 cell lines. Furthermore, hybrid **9a** was also outstanding, with a mean *GI*_50_ value of 0.605 µM, which was the most effective on the HL-60(TB) leukemia cell line (*GI*_50_ = 0.162 µM). However, it should be noted that derivative **12**, containing a more rigid linker, had slightly weaker antitumor activity than the analog containing the more flexible linker.

#### 2.2.2. MTT Assay of Compounds **9g**, **13**, and **16**

The antiproliferative properties of the tested compounds were additionally determined using the MTT assay against a panel of human adherent cancer cell lines isolated from breast (MCF-7 and MDA-MB-231), cervical (HeLa and SiHa), and ovarian (A2780) tumors and also on the NIH/3T3 fibroblast cell line (Table 3). *IC*_50_ values were calculated from a wider concentration range (0.1–30 μM) when higher than 50% cell growth inhibition was obtained at 10 μM (Table 4). Cisplatin was used as a reference compound. Compound **9g**, containing a pyridine ring, demonstrated considerable activity, eliciting higher than 60% cell growth inhibition at 10 μM against all tested cancer cells. As expected, treatments with derivative **13** containing a trityl group instead of the ionic phosphonium part resulted in modest or negligible effect even at 30 μM. Finally, hybrid **16** containing TPP at position 10 of vindoline had no remarkable anticancer activity. Based on the obtained *IC*_50_ values, compound **9g** is more potent than cisplatin against cervical and triple-negative breast cancer cells, while MCF-7 and A2780 cells exhibited similar sensitivities. The noncancerous fibroblast cell line NIH/3T3 was used to gather preliminary data regarding the cancer selectivity of vindolin derivatives. Compound **9g** inhibited the growth of fibroblasts with a substantially higher *IC*_50_ (14.37 μM) than the cancer cells (1.50–6.48 μM). The tumor selectivity of compound **9g**, expressed as the ratio of *IC*_50_ on cancer cells and fibroblasts, is substantially higher than that of cisplatin (Table 4).

#### 2.2.3. Cell Cycle Analysis

**Figure 3:**
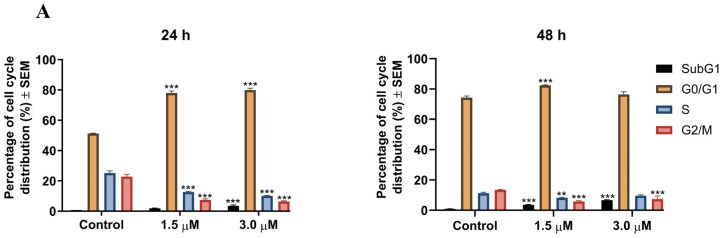

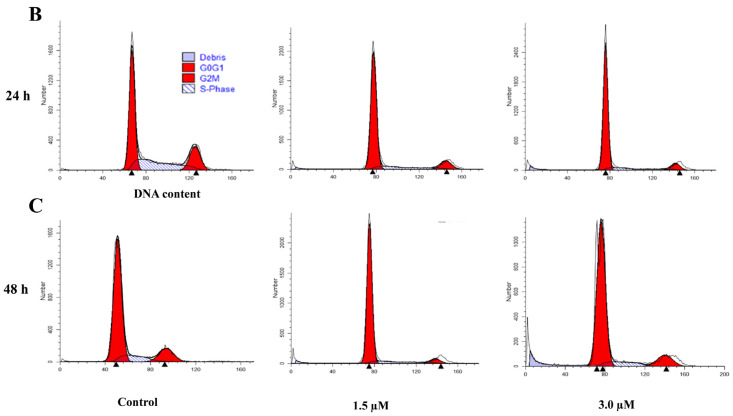
The effect of compound **9g** on the cell cycle distribution of A2780 cells after treatment with 1.5 or 3.0 μM (**A**). ** and *** indicate significance at *p* < 0.01 and *p* < 0.001, respectively, compared to control. Representative histograms after 24 (**B**) and 48 (**C**) hours of treatment.

Based on its promising *IC*_50_ and selectivity, compound **9g** was selected for further investigations, including cell cycle analysis. Treatment with derivative **9g** induced concentration- and time-dependent cell cycle disturbances in A2780 ovarian cancer cells, accompanied by apoptosis (Figure 3). At 24 h, hybrid **9g** (1.5 μM and 3.0 μM) significantly increased the proportion of cells in the G0/G1 phase while decreasing S and G2/M phase populations. After a 48 h exposure, the lower concentration maintained this pattern (G0/G1 elevation and S and G2/M reduction). The higher concentration, however, showed partial cell cycle recovery in the G0/G1 and S phases, while G2/M depletion persisted. In addition, a remarkable increase in the subG1 cell population was observed, particularly after the more prolonged exposure, indicating the proapoptotic property of the tested compound.

#### 2.2.4. Antimigratory Activity

**Figure 4:**
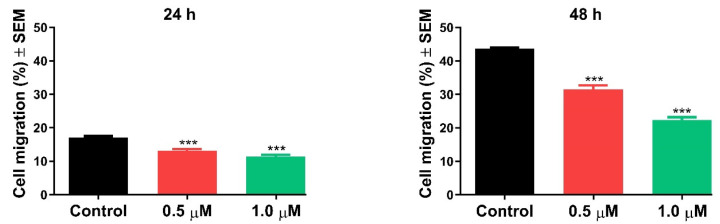

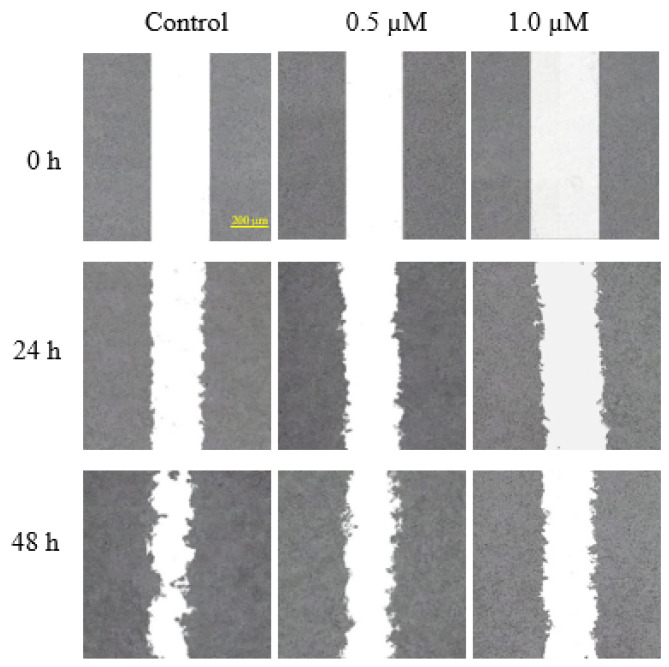
The effect of compound **9g** on the migration of A2780 cells. The graphs indicate the percentage of cell-free areas at 24 h and 48 h post-treatment compared to control (upper panels). *** indicates significance at *p* < 0.001 compared to control. Representative images of reduced wound healing at 0, 24, and 48 h post-treatment (lower panels).

The antimigratory properties of compound **9g** were investigated using the in vitro wound healing assay against the ovarian cancer cell line A2780. The results indicated that derivative **9g** demonstrated a concentration- and duration-dependent reduction in cell migration at sub-inhibitory concentrations, as observed at 24 and 48 h post-treatment, compared to the untreated controls (Figure 4).

The in vitro anticancer screening revealed some interesting structure–activity relationships regarding vindoline–TPP hybrids. The results suggest that (i) small changes in the length of the linker have negligible effects on antitumor activity; (ii) making the linker more rigid is not beneficial for anticancer activity; (iii) the cytotoxic effect can be further enhanced by incorporating a substituted TPP unit, especially in the case of tris(4-methoxy-3,5-dimethylphenylphosphine; (iv) the presence of the phosphorus atom and the ionic nature of the TPP moiety are crucial for the effect; and (v) he substitution of vindoline with TPP seems to be more beneficial in position 17 compared to position 10.

#### 2.2.5. Effect of Selected Conjugates on the Viability of Non-Tumor Chinese Hamster Ovary (CHO) Cells

**Figure 5:**
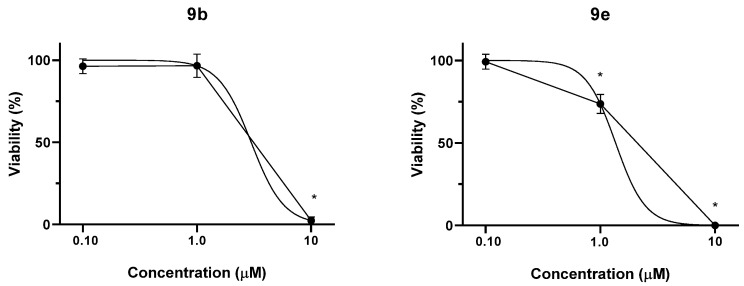
The effect of conjugates **9b** and **9e** on the viability of non-tumor CHO cells. Normalized viability values after 48 h compound treatment are represented as means ± SD of 3 independent experiments in technical triplicates (*n* = 9); one-way ANOVA + Dunnett’s multiple comparison test, * *p* < 0.0001 vs. control.

Conjugates **9b** and **9e** were selected based on their antitumor effects to be tested on non-tumor CHO cells in the CellTiter-Glo Luminescent Cell Viability Assay to investigate their potential selectivity for cancer cells. Following a 48 h treatment with the conjugates in the 0.10–10 µM concentration range, a concentration-dependent decrease in the luminescent signal was observed, indirectly representing the ATP production of living cells as an indicator of cell viability. While the 0.10 µM concentration did not affect CHO cell viability, the 1.0 µM concentration of conjugate **9e** resulted in a 26.4 ± 5.8% decrease in viability and the 0.10 µM treatment with both conjugates completely abolished the luminescent signal representing cytotoxicity (Figure 5). The *IC*_50_ values were estimated to be 2.98 µM and 1.36 µM for conjugates **9b** and **9e**, respectively. Since significant inhibitory effects of the conjugates were achieved at remarkably higher concentrations on the non-tumor CHO cells compared to the investigated tumor cell lines, good selectivity of these compounds on tumor cells can be predicted.

## 3. Materials and Methods

### 3.1. General

All chemicals were purchased from Sigma-Aldrich (Budapest, Hungary) and were used as received. Melting points were measured on a VEB Analytik Dresden PHMK-77/1328 apparatus (Dresden, Germany) and are uncorrected.

**Figure 6:**
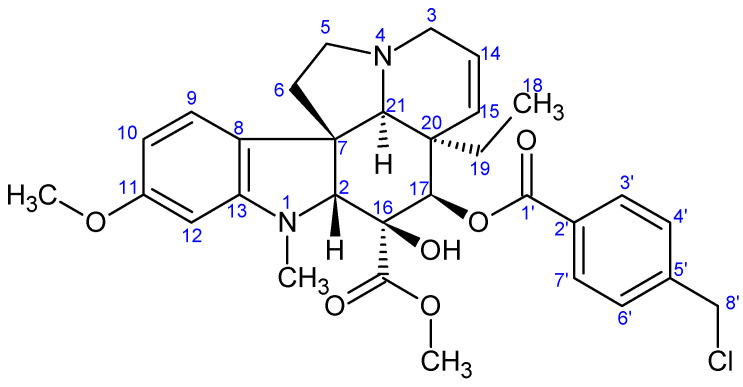
The skeleton numbering of compound **11** used for NMR assignment.

**Figure 7:**
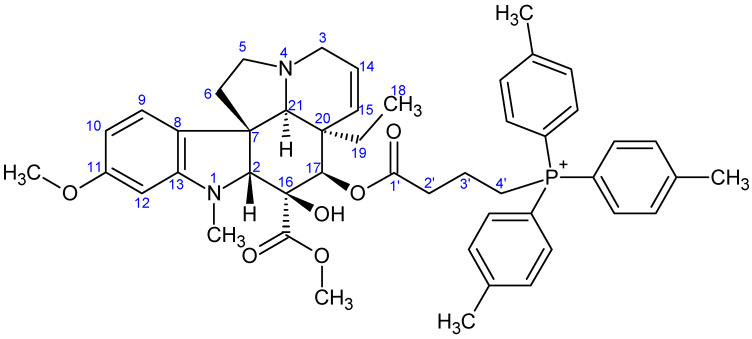
The skeleton numbering of compound **9a** used for NMR assignment.

**Figure 8:**
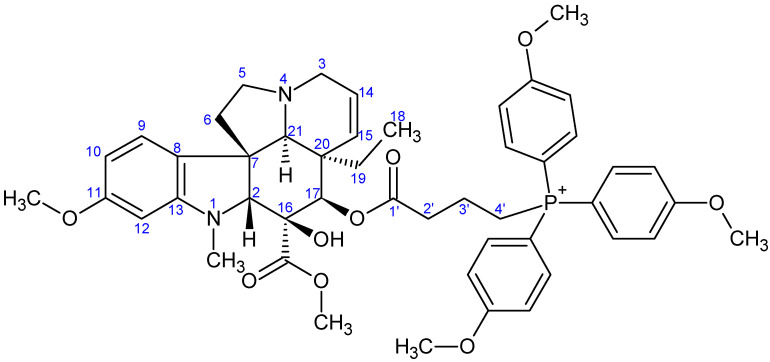
The skeleton numbering of compound **9b** used for NMR assignment.

**Figure 9:**
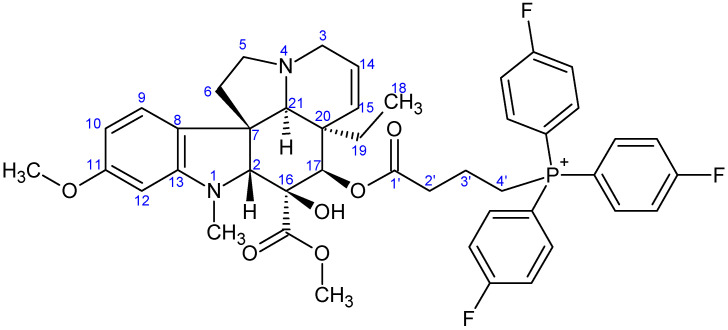
The skeleton numbering of compound **9c** used for NMR assignment.

**Figure 10:**
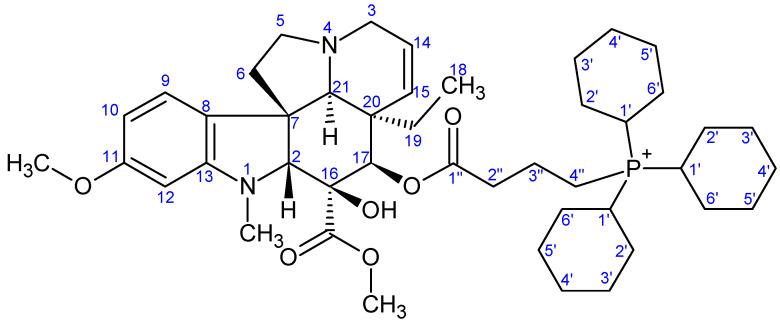
The skeleton numbering of compound **9d** used for NMR assignment.

**Figure 11:**
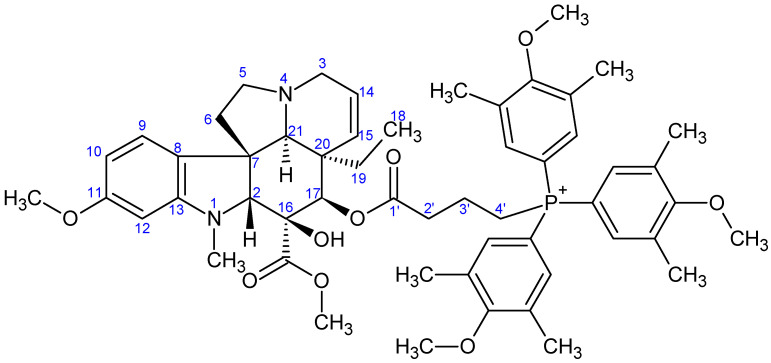
The skeleton numbering of compound **9e** used for NMR assignment.

**Figure 12:**
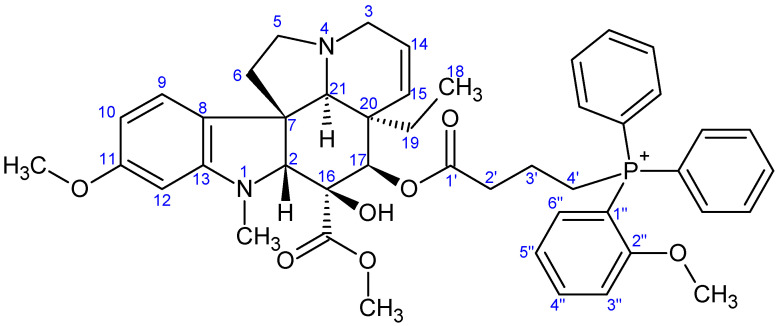
The skeleton numbering of compound **9f** used for NMR assignment.

**Figure 13:**
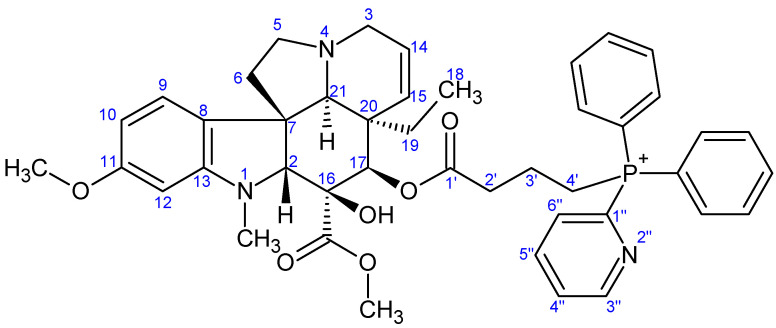
The skeleton numbering of compound **9g** used for NMR assignment.

**Figure 14:**
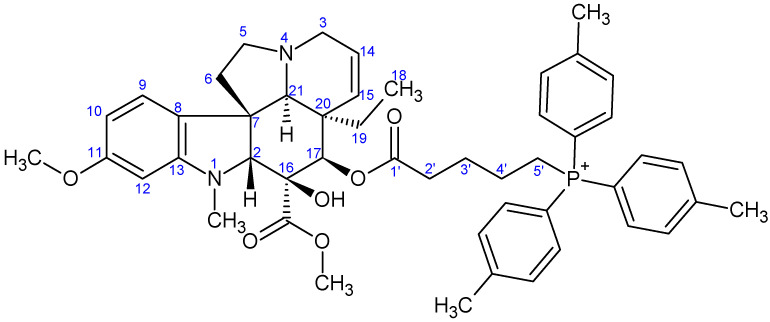
The skeleton numbering of compound **10a** used for NMR assignment.

**Figure 15:**
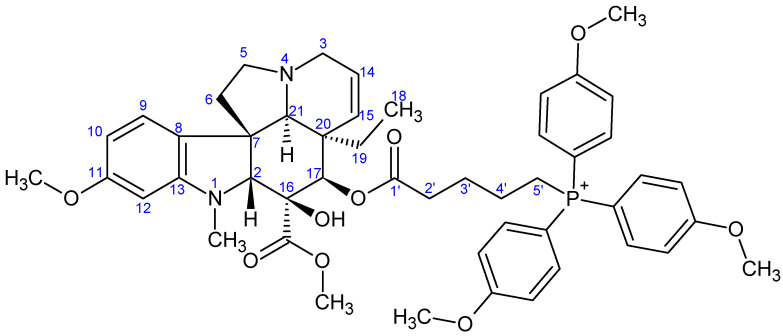
The skeleton numbering of compound **10b** used for NMR assignment.

**Figure 16:**
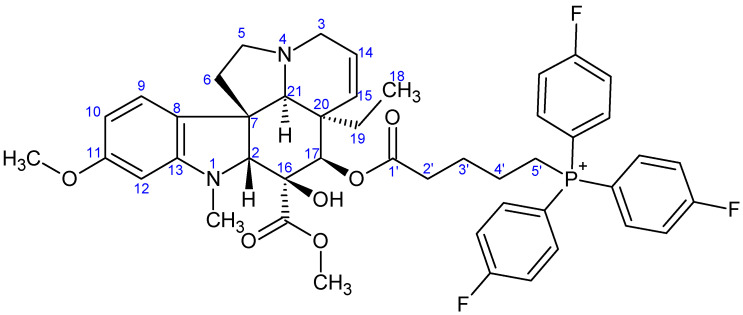
The skeleton numbering of compound **10c** used for NMR assignment.

**Figure 17:**
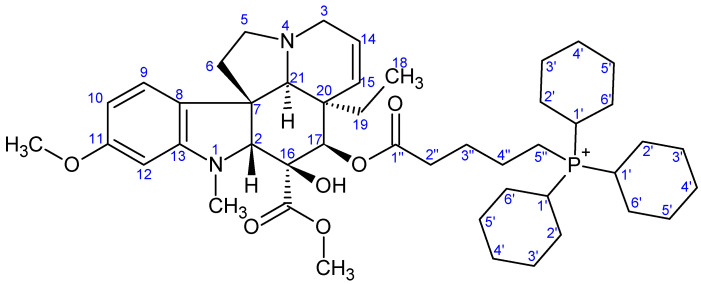
The skeleton numbering of compound **10d** used for NMR assignment.

**Figure 18:**
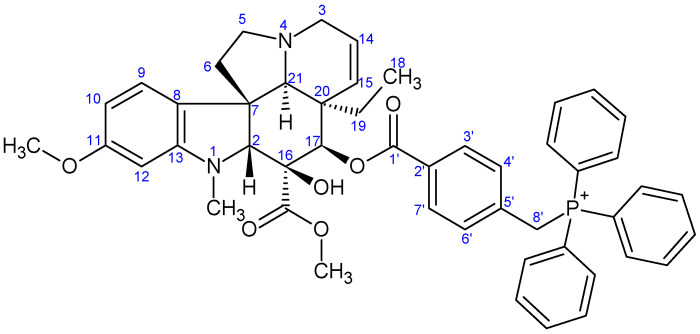
The skeleton numbering of compound **12** used for NMR assignment.

**Figure 19:**
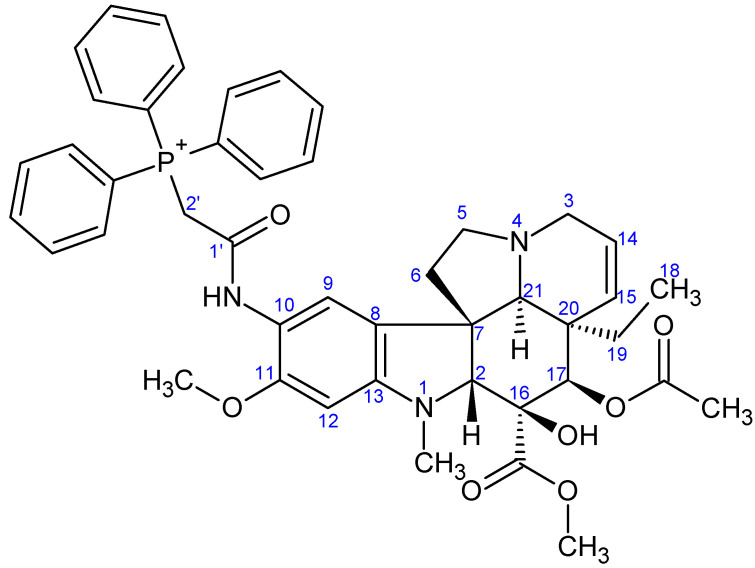
The skeleton numbering of compound **16** used for NMR assignment.

**Figure 20:**
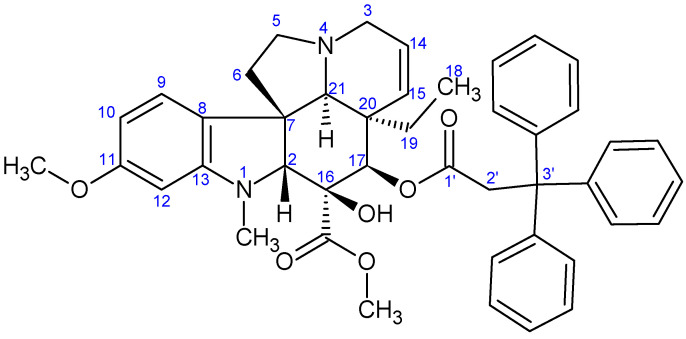
The skeleton numbering of compound **13** used for NMR assignment.

NMR measurements were performed on a Bruker Avance III HDX 400 MHz NMR spectrometer equipped with a ^31^P–^15^N{1H–^19^F} 5 mm CryoProbe Prodigy BBO probe (Bruker Corporation, Billerica, MA, USA), a Bruker Avance III HDX 500 MHz NMR spectrometer equipped with a ^1^H{^13^C/^15^N} 5 mm TCI CryoProbe, a Varian VNMRS 600 MHz NMR System NMR spectrometer, and a Bruker Avance III HDX 800 MHz NMR spectrometer equipped with a ^1^H–^19^F{^13^C/^15^N} 5 mm TCI CryoProbe (Bruker Corporation, Billerica, MA, USA). ^1^H And ^13^C chemical shifts are given on the delta scale as parts per million (ppm) relative to tetramethyl silane. One-dimensional ^1^H, and ^13^C spectra and two-dimensional ^1^H–^1^H COSY, ^1^H–^1^H NOESY, ^1^H–^13^C HSQC, and ^1^H–^13^C HMBC spectra were acquired using pulse sequences included in the standard spectrometer software packages (Bruker TopSpin 3.5, Bruker Corporation; VNMRJ 3.2, Agilent Technologies, Santa Clara, CA, USA). NMR spectra were processed with Bruker TopSpin 3.5 pl 6 (Bruker Corporation, Billerica, MA, USA) and ACD/Spectrus Processor version 2017.1.3 (Advanced Chemistry Development, Inc., Toronto, ON, Canada). The skeleton numbering of compounds **9a**–**g**, **10a**–**d**, **11**–**13**, and **16** used for NMR assignment are presented in Figure 6, Figure 7, Figure 8, Figure 9, Figure 10, Figure 11, Figure 12, Figure 13, Figure 14, Figure 15, Figure 16, Figure 17, Figure 18, Figure 19 and Figure 20. The ^1^H NMR, ^13^C NMR and HRMS spectra of all the newly synthesized compounds (**9a**−**g**, **10a**−**d**, **11**−**13**, and **16**) can be found in the Supplementary Materials (Figures S1–S45).

ESI-HRMS and MS-MS analyses were performed on a Thermo Velos Pro Orbitrap Elite (Thermo Fisher Scientific, Bremen, Germany) system. The ionization method was ESI, operated in positive ion mode. The protonated molecular ion peaks were fragmented by CID (collision-induced dissociation) at a normalized collision energy of 40–45%. For the CID experiment, helium was used as the collision gas. Data acquisition and analysis were accomplished with Xcalibur software version 4.0 (Thermo Fisher Scientific).

The reactions were followed by analytical thin layer chromatography (TLC) on DC-Alufolien Kieselgel 60 F_254_ (Merck, Budapest, Hungary) plates. Preparative TLC analyses were performed on silica gel 60 PF_254+366_ (Merck) glass plates. Column chromatography was carried out using Silica gel 60 (0.040–0.063 mm) (Merck).

### 3.2. Chemistry

#### 3.2.1. Synthesis of the Linker-Containing Vindoline Derivatives (**7**, **8**, **11**, and **15**) and Their Precursors (**6** and **14**)

**Preparation of 17-desacetylvindoline (6)**

^1^H NMR, m.p., and *R_f_* data were in good agreement with the literature [37,42].

**Preparation of 17-(*O*-4-bromobutanoyl)vindoline (7)**

^1^H NMR, m.p., and *R_f_* data were in good agreement with the literature [36,42].

**Preparation of 17-(*O*-5-bromopentanoyl)vindoline (8)**

^1^H NMR, m.p., and *R_f_* data were in good agreement with the literature [36].

**Preparation of 17-(*O*-4-(chloromethyl)benzoyl)vindoline (11)**

First, 184 mg (0.854 mmol, 1.2 eq.) of 4-(bromomethyl)benzoic acid was dissolved in 5 mL of dry DCM. Then, 0.25 mL (3.42 mmol, 4.0 eq.) of thionyl chloride and 4 drops of DMF were added to the solution. The mixture was stirred at 50 °C under an Ar atmosphere for 3 h, after which an additional 0.25 mL (3.42 mmol, 4.0 eq.) of thionyl chloride was added and the temperature was increased to 60 °C. After a total of 5 h of stirring, the reaction mixture was evaporated to dryness and then dissolved in 2 mL of dry DCM (solution B). Next, 295 mg (0.712 mmol) of 17-deacetylvindoline (**6**) was dissolved in 5 mL of dry DCM and 0.24 mL (1.71 mmol, 2.4 eq.) of NEt_3_ was added (solution A). Solution A was cooled to 0 °C using an ice-water bath and the previously prepared solution B was added dropwise. The mixture was stirred at room temperature for 2 h. After completion of the reaction, 5 mL of distilled water was added to the mixture. After phase separation, the water phase was extracted with 3 × 15 mL DCM. The organic phases were combined and dried over MgSO_4_, then filtered, and the drying agent was washed with DCM. The solvent was evaporated and the crude product was purified by preparative TLC (DCM:MeOH = 15:1). Finally, 114 mg (28%) of product **20** was isolated. M.p.: 110–112 °C. TLC (DCM:MeOH = 15:1); *R_f_* = 0.85. ^1^H NMR (499.9 MHz; DMSO-*d*_6_): *δ* (ppm) 0.43 (3H; t; *J* = 7.3 Hz; H_3_-18); 1.07 (1H; dq; *J* = 14.2, 7.3 Hz; H_x_-19); 1.61 (1H; dq; *J* = 14.2, 7.3 Hz; H_y_-19); 2.24–2.30 (2H; m; H_2_-6); 2.56–2.64 (4H; N(1)-CH_3_, H_x_-5); 2.69 (1H; s; H-21); 2.82 (1H; br d; *J* = 16.4 Hz; H_x_-3); 3.26–3.31 (1H; m; H_y_-5); 3.48 (1H; br dd; *J* = 16.4, 4.7 Hz; H_y_-3); 3.57 (3H; s; C(16)-COOCH_3_); 3.64 (1H; s; H-2); 3.72 (3H; s; C(11)-OCH_3_); 4.82 (2H; s; H_2_-8′); 5.06 (1H; br d; *J* = 10.1 Hz; H-15); 5.44 (1H; s; H-17); 5.81 (1H; ddd; *J* = 10.1, 4.7, 1.2 Hz; H-14); 6.24 (1H; d; *J* = 2.2 Hz; H-12); 6.32 (1H; dd; *J* = 8.2, 2.2 Hz; H-10); 7.09 (1H; d; *J* = 8.2 Hz; H-9); 7.56–7.60 (2H; m; H-4′, H-6′); 7.85–7.90 (2H; m; H-3′, H-7′); 8.95 (1H; s; C(16)-OH). ^13^C NMR (125.7 MHz; DMSO-*d*_6_): *δ* (ppm) 7.5 (C-18); 30.6 (C-19); 38.0 (N(1)-CH_3_); 42.7 (C-20); 43.5 (C-6); 45.1 (C-8′); 50.5 (C-3); 51.3 (C-5); 51.7 (C(16)-COOCH_3_); 52.0 (C-7); 55.0 (C(11)-OCH_3_); 66.3 (C-21); 76.7 (C-17); 78.7 (C-16); 82.7 (C-2); 95.6 (C-12); 104.6 (C-10); 123.1 (C-9); 124.7 (C-14); 125.4 (C-8); 129.0 (C-4′, C-6′); 129.4 (C-3′, C-7′); 129.5 (C-15); 129.6 (C-2′); 142.7 (C-5′); 153.5 (C-13); 160.5 (C-11); 165.0 (C-1′); 171.6 (C(16)-COOCH_3_). HRMS: M+H = 567.22529 (delta = −0.6 ppm; C_31_H_36_O_6_N_2_Cl). HR-ESI-MS-MS (CID = 40%; rel. int. %): 549(10); 507(12); 397(100); 188(21).

**Preparation of 10-aminovindoline (14)**

^1^H NMR, m.p., and *R_f_* data were in good agreement with the literature [42].

**Preparation of 10-chloroacetamidovindoline (15)**

^1^H NMR, m.p., and *R_f_* data were in good agreement with the literature [40,42].

#### 3.2.2. Synthesis of Vindoline–TPP Phosphonium Salts (**9a**–**g**, **10a**–**d**, **12**, and **16**)

**Preparation of product 9a**

First, 50 mg (0.089 mmol) of 17-(*O*-4-bromobutanoyl)vindoline (**7**) and 54 mg (0.178 mmol, 5.0 eq.) tri(*p*-tolyl)phosphine (**5a**) were dissolved in 3 mL of dry acetonitrile. The mixture was stirred at reflux temperature under an Ar atmosphere. After one day, an additional 54 mg (0.178 mmol, 2.0 eq.) of tri(*p*-tolyl)phosphine (**5a**) was added to the mixture. After 23 h of stirring, the mixture was evaporated under reduced pressure and the crude product was purified by preparative TLC (DCM:MeOH = 10:1). After purification, 38 mg (49%) of product **9a** was isolated. M.p.: 151–153 °C. TLC (DCM:MeOH = 10:1); *R_f_* = 0.32. ^1^H NMR (499.9 MHz; DMSO-*d*_6_) *δ* (ppm): 0.41 (3H; t; *J* = 7.3 Hz; H_3_-18); 0.94 (1H; dq; *J* = 14.3, 7.3 Hz; H_x_-19); 1.45 (1H; dq; *J* = 14.3, 7.3 Hz; H_y_-19); 1.64–1.76 (2H; m; H_2_-3′); 2.18–2.24 (2H; m; H_2_-6); 2.41–2.53 (11H; m; H_2_-2′, 3×P-Ph-CH_3_); 2.52–2.61 (4H; m; N(1)-CH_3_, H_x_-5); 2.66 (1H; s; H-21); 2.80 (1H; br d; *J* = 16.4 Hz; H_x_-3); 3.25–3.37 (2H; m; H_y_-3, H_y_-5); 3.38–3.49 (2H; m; H_2_-4′); 3.56 (3H; s; C(16)-COOCH_3_); 3.57 (1H; s; H-2); 3.70 (3H; s; C(11)-OCH_3_); 5.07 (1H; br d; *J* = 10.2 Hz; H-15); 5.20 (1H; s; H-17); 5.71 (1H; ddd; *J* = 10.1, 4.9, 1.4 Hz; H-14); 6.19 (1H; d; *J* = 2.2 Hz; H-12); 6.29 (1H; dd; *J* = 8.2, 2.2 Hz; H-10); 7.06 (1H; d; *J* = 8.2 Hz; H-9); 7.56–7.60 (6H; m; 6×P-Ph: H_meta_); 7.61–7.68 (6H; m; 6×P-Ph: H_orto_); 8.89 (1H; s; C(16)-OH). ^13^C NMR (125.7 MHz; DMSO-*d*_6_) *δ* (ppm): 7.5 (C-18); 17.7 (C-3′) 20.1 (d; *J* = 52.0 Hz; C-4′); 21.1 (3×P-Ph-CH_3_); 30.4 (C-19); 33.6 (d; *J* = 18.3 Hz; C-2′); 38.0 (N(1)-CH_3_); 42.3 (C-20); 43.5 (C-6); 50.3 (C-3); 51.0 (C-5); 51.6 (C(16)-COOCH_3_); 52.0 (C-7); 55.0 (C(11)-OCH_3_); 66.0 (C-21); 76.2 (C-17); 78.7 (C-16); 82.6 (C-2); 95.5 (C-12); 104.5 (C-10); 115.0 (d; *J* = 88.3 Hz; 3×P-Ph: C_ipszo_); 123.1 (C-9); 124.4 (C-14); 125.2 (C-8); 129.8 (C-15); 130.8 (d; *J* = 12.8 Hz; 6×P-Ph: C_meta_); 133.3 (d; *J* = 10.6 Hz; 6×P-Ph: C_orto_); 145.7 (d; *J* = 2.4 Hz; 3×P-Ph: C_para_); 153.3 (C-13); 160.5 (C-11); 171.2 (C-1′); 171.6 (C(16)-COOCH_3_). HRMS: M+ = 787.38248 (delta= −5.8 ppm; C_48_H_56_O_6_N_2_P). HR-ESI-MS-MS (CID = 40%; rel. int. %): 391(100); 373(5).

**Preparation of product 9b**

First, 17-(*O*-4-bromobutanoyl)vindoline (**7**) (50 mg, 0.089 mmol) and tris(4-methoxyphenyl)phosphine (**5b**) (189 mg, 0.534 mmol, 6.0 eq.) were dissolved in dry acetonitrile (3 mL). The mixture was stirred at reflux temperature under an Ar atmosphere for 10 h, then evaporated under reduced pressure. Then, 58 mg (72%) of pale orange crystalline product **9b** was isolated after preparative TLC (DCM:MeOH = 10:1). M.p.: 136–138 °C. TLC (DCM:MeOH = 10:1); *R_f_* = 0.33. ^1^H NMR (499.9 MHz; DMSO-*d*_6_) *δ* (ppm): 0.42 (3H; t; *J* = 7.3 Hz; H_3_-18); 0.95 (1H; dq; *J* = 14.3, 7.3 Hz; H_x_-19); 1.46 (1H; dq; *J* = 14.3, 7.3 Hz; H_y_-19); 1.64–1.76 (2H; m; H_2_-3′); 2.18–2.24 (2H; m; H_2_-6); 2.41–2.62 (6H; m; N(1)-CH_3_, H_x_-5, H_2_-2′); 2.67 (1H; s; H-21); 2.80 (1H; br d; *J* = 16.4 Hz; H_x_-3); 3.24–3.44 (4H; m; H_y_-3, H_y_-5, H_2_-4′); 3.56 (3H; s; C(16)-COOCH_3_); 3.57 (1H; s; H-2); 3.70 (3H; s; C(11)-OCH_3_); 3.89 (9H; s; 3×P-Ph-OCH_3_); 5.07 (1H; br d; *J* = 10.1 Hz; H-15); 5.20 (1H; s; H-17); 5.71 (1H; ddd; *J* = 10.1, 4.9, 1.4 Hz; H-14); 6.19 (1H; d; *J* = 2.3 Hz; H-12); 6.29 (1H; dd; *J* = 8.2, 2.3 Hz; H-10); 7.06 (1H; d; *J* = 8.2 Hz; H-9); 7.27–7.32 (6H; m; 6×P-Ph: H_meta_); 7.62–7.71 (6H; m; 6×P-Ph: H_orto_); 8.89 (1H; s; C(16)-OH). ^13^C NMR (125.7 MHz; DMSO-*d*_6_) *δ* (ppm): 7.5 (C-18); 17.7 (C-3′); 20.8 (d; *J* = 53.7 Hz; C-4′); 30.4 (C-19); 33.6 (d; *J* = 18.2 Hz; C-2′); 38.0 (N(1)-CH_3_); 42.3 (C-20); 43.5 (C-6); 50.3 (C-3); 51.0 (C-5); 51.6 (C(16)-COOCH_3_); 52.0 (C-7); 55.0 (C(11)-OCH_3_); 55.8 (3×P-Ph-OCH_3_); 66.0 (C-21); 76.2 (C-17); 78.7 (C-16); 82.6 (C-2); 95.5 (C-12); 104.5 (C-10); 109.1 (d; J = 93.7 Hz; 3×P-Ph: C_ipszo_); 115.9 (d; *J* = 13.6 Hz; 6×P-Ph: C_meta_); 123.1 (C-9); 124.4 (C-14); 125.2 (C-8); 129.8 (C-15); 135.3 (d; *J* = 11.7 Hz; 6×P-Ph: C_orto_); 153.3 (C-13); 160.5 (C-11); 163.9 (d; *J* = 2.3 Hz; 3×P-Ph: C_para_); 171.3 (C-1′); 171.6 (C(16)-COOCH_3_). HRMS: M+ = 835.36778 (delta = −4.8 ppm; C_48_H_56_O_9_N_2_P). HR-ESI-MS-MS (CID = 40%; rel. int. %): 439(100); 421(5).

**Preparation of product 9c**

First, 50 mg (0.089 mmol) of 17-(*O*-4-bromobutanoyl)vindoline (**7**) and 168 mg (0.534 mmol, 6.0 eq.) of tris(4-fluorophenyl)phosphine (**5c**) were dissolved in 3 mL of dry acetonitrile. The reaction mixture was stirred under an argon atmosphere at reflux temperature. After 37 h, the reaction mixture was worked up. After evaporation, the crude product was washed with *tert*-butyl methyl ether and purified by preparative TLC (DCM:MeOH = 15:1). After purification, 23 mg (29%) of the target compound **9c** was isolated. M.p.: 132–134 °C. TLC (DCM:MeOH = 10:1); *R_f_* = 0.34. ^1^H NMR (499.9 MHz; DMSO-*d*_6_) *δ* (ppm): 0.42 (3H; t; *J* = 7.2 Hz; H_3_-18); 0.94 (1H; dq; *J* = 14.3, 7.2 Hz; H_x_-19); 1.46 (1H; dq; *J* = 14.3, 7.3 Hz; H_y_-19); 1.65–1.78 (2H; m; H_2_-3′); 2.17–2.25 (2H; m; H_2_-6); 2.41–2.62 (6H; m; N(1)-CH_3_, H_x_-5, H_2_-2′); 2.67 (1H; s; H-21); 2.80 (1H; br d; *J* = 16.2 Hz; H_x_-3); 3.25–3.39 (2H; m; H_y_-3, H_y_-5); 3.51–3.61 (6H; m; H-2, C(16)-COOCH_3_, H_2_-4′); 3.70 (3H; s; C(11)-OCH_3_); 5.08 (1H; br d; *J* = 10.2 Hz; H-15); 5.20 (1H; s; H-17); 5.73 (1H; br dd; *J* = 10.1, 4.8, Hz; H-14); 6.19 (1H; d; *J* = 2.2 Hz; H-12); 6.29 (1H; dd; *J* = 8.3, 2.2 Hz; H-10); 7.06 (1H; d; *J* = 8.3 Hz; H-9); 7.63–7.70 (6H; m; 6×P-Ph: H_meta_); 7.83–7.95 (6H; m; 6×P-Ph: H_orto_); 8.92 (1H; s; C(16)-OH). ^13^C NMR (125.7 MHz; DMSO-*d*_6_) *δ* (ppm): 7.5 (C-18); 17.5 (d; *J* = 2.7 Hz; C-3′) 20.2 (d; *J* = 51.7 Hz; C-4′); 30.4 (C-19); 33.5 (d; *J* = 18.6 Hz; C-2′); 38.0 (N(1)-CH_3_); 42.3 (C-20); 43.5 (C-6); 50.3 (C-3); 51.0 (C-5); 51.7 (C(16)-COOCH_3_); 52.0 (C-7); 55.0 (C(11)-OCH_3_); 65.9 (C-21); 76.2 (C-17); 78.7 (C-16); 82.6 (C-2); 95.5 (C-12); 104.5 (C-10); 114.2 (dd; *J* = 90.6, 3.2 Hz; 3×P-Ph: C_ipszo_); 117.9 (dd; *J* = 22.3, 14.0 Hz; 6×P-Ph: C_meta_); 123.1 (C-9); 124.4 (C-14); 125.2 (C-8); 129.8 (C-15); 136.9 (dd; *J* = 12.1, 10.0 Hz; 6×P-Ph: C_orto_); 153.3 (C-13); 160.5 (C-11); 166.0 (dd; *J* = 256.0, 2.4 Hz; 3×P-Ph: C_para_); 171.1 (C-1′); 171.6 (C(16)-COOCH_3_). HRMS: M+ = 799.30908 (delta = −3.5 ppm; C_45_H_47_O_6_N_2_F_3_P). HR-ESI-MS-MS (CID = 40%; rel. int. %): 403(100); 385(6).

**Preparation of product 9d**

First, 50 mg (0.089 mmol) of 17-(*O*-4-bromobutanoyl)vindoline (**7**) and 150 mg (0.534 mmol, 6.0 eq.) of tricyclohexylphosphine (**5d**) were dissolved in 3 mL of dry acetonitrile. The mixture was stirred at reflux temperature under an argon atmosphere for 10 h. Subsequently, the reaction mixture was evaporated and washed with *tert*-butyl methyl ether. The crude product was purified by preparative TLC (DCM:MeOH = 20:1), yielding 45 mg (60%) of product **9d**. M.p.: 106–107 °C. TLC (DCM:MeOH = 15:1); *R_f_* = 0.31. ^1^H NMR (399.8 MHz; CDCl_3_) *δ* (ppm): 0.50 (3H; t; *J* = 7.3 Hz; H_3_-18); 1.14 (1H; dq; *J* = 14.4, 7.3 Hz; H_x_-19); 1.23–1.38 (3H; m; 3×H_x_-4′); 1.40–1.65 (13H; m; H_y_-19, 3×H_x_-2′, 3×H_x_-3′, 3×H_x_-5′, 3×H_x_-6′); 1.71–2.13 (17H; m; 3×H_y_-2′, 3×H_y_-3′, 3×H_y_-4′, 3×H_y_-5′, 3×H_y_-6′, H_2_-3″); 2.27–2.39 (2H; m; H_2_-6); 2.47–2.77 (12H; m; N(1)-CH_3_, H_x_-5, H-21, 3×H-1′, H_2_-2″, H_2_-4″); 2.84 (1H; br d; *J* = 16.0 Hz; H_x_-3); 3.37–3.54 (2H; m; H_y_-3, H_y_-5); 3.75 (H-2); 3.79 (3H; s; C(11)-OCH_3_); 3.80 (3H; s; C(16)-COOCH_3_); 5.22 (1H; br d; *J* = 10.0 Hz; H-15); 5.45 (1H; s; H-17); 5.90 (1H; ddd; *J* = 10.0, 4.7, 1.4 Hz; H-14); 6.08 (1H; d; *J* = 2.2 Hz; H-12); 6.31 (1H; dd; *J* = 8.2, 2.2 Hz; H-10); 6.91 (1H; d; *J* = 8.2 Hz; H-9); 9.51 (1H; br s; C(16)-OH). ^13^C NMR (100.5 MHz; CDCl_3_) *δ* (ppm): 7.7 (C-18); 15.4 (d; *J* = 43.9 Hz; C-4″); 18.3 (d; *J* = 4.4 Hz; C-3″); 25.5 (d; *J* = 0.9 Hz; 3×C-4′); 26.5 (d; *J* = 12.0 Hz; 3×C-3′, 3×C-5′); 27.2 (d; *J* = 4.2 Hz; 3×C-2′, 3×C-6′); 30.0 (d; *J* = 40.4 Hz; 3×C-1′); 31.0 (C-19); 34.2 (d; *J* = 14.9 Hz; C-2″); 38.3 (N(1)-CH_3_); 42.9 (C-20); 44.0 (C-6); 51.1 (C-3); 52.0 (C-5); 52.3 (C(16)-COOCH_3_); 52.8 (C-7); 55.4 (C(11)-OCH_3_); 67.1 (C-21); 76.7 (C-17); 79.5 (C-16); 83.4 (C-2); 96.0 (C-12); 104.5 (C-10); 122.8 (C-9); 124.5 (C-14); 125.2 (C-8); 130.2 (C-15); 153.7 (C-13); 161.2 (C-11); 172.1 (C(16)-COOCH_3_); 172.4 (C-1′). HRMS: M+ = 763.47982 (delta = −1.5 ppm; C_45_H_68_O_6_N_2_P). HR-ESI-MS-MS (CID = 40%; rel. int. %): 367(100); 349(7); 285(4).

**Preparation of product 9e**

First, 60 mg (0.106 mmol) of 17-(*O*-4-bromobutanoyl)vindoline (**7**) and 140 mg (0.319 mmol, 3.0 eq.) of tris(4-methoxy-3,5-dimethylphenyl)phosphine (**5e**) were measured into a pressure-resistant vessel, then dissolved in 5 mL of dry acetonitrile. The mixture was stirred at 115 °C under an argon atmosphere for 14 h, then filtered. The solution was evaporated and the crude product was purified by preparative TLC (DCM:MeOH = 15:1), yielding 78 mg (74%) of the product **9e**. M.p.: 114–116 °C. TLC (DCM:MeOH = 15:1); *R_f_* = 0.48. ^1^H NMR (599.8 MHz; DMSO-*d*_6_) *δ* (ppm): 0.42 (3H; t; *J* = 7.4 Hz; H_3_-18); 0.95 (1H; dq; *J* = 14.2, 7.3 Hz; H_x_-19); 1.48 (1H; dq; *J* = 14.2, 7.4 Hz; H_y_-19); 1.57–1.74 (2H; m; H_2_-3′); 2.17–2.24 (2H; m; H_2_-6); 2.30 (18H; s; 6×P-Ar: CH_3_); 2.38–2.48 (1H; m; H_x_-2′); 2.48–2.62 (5H; m; N(1)-CH_3_, H_x_-5, H_y_-2′); 2.67 (1H; s; H-21); 2.80 (1H; br d; *J* = 16.5 Hz; H_x_-3); 3.24–3.44 (4H; m; H_y_-3, H_y_-5, H_2_-4′); 3.54 (3H; s; C(16)-COOCH_3_); 3.56 (1H; s; H-2); 3.70 (3H; s; C(11)-OCH_3_); 3.77 (9H; s; 3×P-Ar: OCH_3_); 5.08 (1H; br d; *J* = 10.1 Hz; H-15); 5.21 (1H; s; H-17); 5.72 (1H; ddd; *J* = 10.1, 4.8, 1.3 Hz; H-14); 6.18 (1H; d; *J* = 2.2 Hz; H-12); 6.29 (1H; dd; *J* = 8.3, 2.2 Hz; H-10); 7.06 (1H; d; *J* = 8.2 Hz; H-9); 7.49 (6H; d; *J* = 12.6 Hz; 6×P-Ar: H_orto_); 8.84 (1H; s; C(16)-OH). ^13^C NMR (150.8 MHz; DMSO-*d*_6_) *δ* (ppm): 7.5 (C-18); 15.9 (6×P-Ar: CH_3_); 17.7 (C-3′); 20.8 (d; *J* = 53.7 Hz; C-4′); 30.3 (C-19); 33.5 (d; *J* = 18.0 Hz; C-2′); 38.0 (N(1)-CH_3_); 42.3 (C-20); 43.5 (C-6); 50.3 (C-3); 51.0 (C-5); 51.5 (C(16)-COOCH_3_); 52.0 (C-7); 55.0 (C(11)-OCH_3_); 59.5 (3×P-Ar: OCH_3_); 66.0 (C-21); 76.2 (C-17); 78.7 (C-16); 82.6 (C-2); 95.5 (C-12); 104.5 (C-10); 113.0 (d; J = 89.0 Hz; 3×P-Ar: C_ipszo_); 123.1 (C-9); 124.4 (C-14); 125.2 (C-8); 129.8 (C-15); 133.1 (d; *J* = 13.8 Hz; 6×P-Ar: C_meta_); 133.7 (d; *J* = 11.3 Hz; 6×P-Ar: C_orto_); 153.3 (C-13); 160.5 (C-11); 161.7 (d; *J* = 3.4 Hz; 3×P-Ar: C_para_); 171.6 (C-1′); 171.7 (C(16)-COOCH_3_). HRMS: M+ = 919.46619 (delta = 0.5 ppm; C_54_H_68_N_2_O_9_P). HR-ESI-MS-MS (CID = 45%; rel. int. %): 523(100); 508(8); 505(5).

**Preparation of product 9f**

First, 80 mg (0.142 mmol) of 17-(*O*-4-bromobutanoyl)vindoline (**7**) and 125 mg (0.426 mmol, 3.0 eq.) of diphenyl(2-methoxyphenyl)phosphine (**5f**) were measured into a pressure-resistant vessel and dissolved in 5 mL of dry acetonitrile. The reaction was carried out under an argon atmosphere and stirred at 90 °C for 24 h. After completion, the mixture was evaporated and purified by preparative TLC (DCM:MeOH = 12:1). After purification, 86 mg (70%) of product **9f** was isolated. M.p.: 130–132 °C. TLC (DCM:MeOH = 12:1); *R_f_* = 0.59. ^1^H NMR (599.8 MHz; DMSO-*d*_6_) *δ* (ppm): 0.41 (3H; t; *J* = 7.3 Hz; H_3_-18); 0.94 (1H; dq; *J* = 14.2, 7.2 Hz; H_x_-19); 1.45 (1H; dq; *J* = 14.2, 7.4 Hz; H_y_-19); 1.62–1.75 (2H; m; H_2_-3′); 2.17–2.24 (2H; m; H_2_-6); 2.43–2.61 (6H; m; N(1)-CH_3_, H_x_-5, H_2_-2′); 2.66 (1H; s; H-21); 2.80 (1H; br d; *J* = 16.5 Hz; H_x_-3); 3.28 (1H; dt; *J* = 9.6, 6.3 Hz; H_y_-5); 3.31–3.39 (1H; m; H_y_-3); 3.40–3.48 (2H; m; H_2_-4′); 3.55 (3H; s; C(16)-COOCH_3_); 3.56 (1H; s; H-2); 3.70 (3H; s; C(2″)-OCH_3_); 3.70 (3H; s; C(11)-OCH_3_); 5.06 (1H; br d; *J* = 10.0 Hz; H-15); 5.20 (1H; s; H-17); 5.72 (1H; ddd; *J* = 10.0, 4.8, 1.3 Hz; H-14); 6.19 (1H; d; *J* = 2.2 Hz; H-12); 6.29 (1H; dd; *J* = 8.2, 2.3 Hz; H-10); 7.06 (1H; d; *J* = 8.2 Hz; H-9); 7.33 (1H; tdd; *J* = 7.6, 2.9, 0.8 Hz; H-5″); 7.38–7.44 (2H; m; H-3″, H-6″); 7.70–7.78 (8H; m; 4×P-Ph: H_orto_, 4×P-Ph: H_meta_); 7.83–7.90 (2H; m; 2×P-Ph: H_para_); 7.92 (1H; ~tt; *J* = 7.9, 1.2 Hz; H-4″); 8.87 (1H; s; C(16)-OH). ^13^C NMR (150.8 MHz; DMSO-*d*_6_) *δ* (ppm): 7.6 (C-18); 18.4 (br s; C-3′) 20.9 (d; *J* = 52.0 Hz; C-4′); 30.5 (C-19); 33.8 (d; *J* = 18.6 Hz; C-2′); 38.1 (N(1)-CH_3_); 42.4 (C-20); 43.6 (C-6); 50.4 (C-3); 51.1 (C-5); 51.7 (C(16)-COOCH_3_); 52.1 (C-7); 55.1 (C(11)-OCH_3_); 56.3 (C(2″)-OCH_3_); 66.1 (C-21); 76.3 (C-17); 78.8 (C-16); 82.7 (C-2); 95.6 (C-12); 104.6 (C-10); 105.3 (d; *J* = 89.0 Hz; C-1″); 113.5 (d; *J* = 6.9 Hz; C-3″); 118.5 (2×d; *J* = 87.9 Hz; 2×P-Ph: C_ipszo_); 122.3 (d; *J* = 12.7 Hz; C-5″); 123.2 (C-9); 124.5 (C-14); 125.3 (C-8); 129.9 (C-15); 130.0 (d; 12.7; 4×P-Ph: C_meta_); 133.0 (2×d; *J* = 10.4 Hz; 4×P-Ph: C_orto_); 134.5 (C-6″); 134.9 (d; *J* = 8.1 Hz; 2×P-Ph: C_para_); 138.0 (C-4″); 153.4 (C-13); 160.6 (C-11); 161.8 (C-2″); 171.4 (C-1′); 171.7 (C(16)-COOCH_3_). HRMS: M+H = 775.35000 (delta = −0.9 ppm; C_46_H_52_O_7_N_2_P). HR-ESI-MS-MS (CID = 45%; rel. int. %): 379(100); 361(5); 277(2).

**Preparation of product 9g**

First, 80 mg (0.142 mmol) of 17-(*O*-4-bromobutanoyl)vindoline (**7**) was dissolved in 5 mL of dry acetonitrile and 224 mg (0.852 mmol, 6.0 eq.) of diphenyl-2-pyridylphosphine (**5g**) was added. The mixture was stirred at 95 °C under an argon atmosphere for 17 h. After the reaction, the mixture was evaporated and purified by preparative TLC (DCM:MeOH = 10:1), yielding 55 mg of the product. Analytical examinations indicated that the compound was not sufficiently pure, so further purification was performed by preparative TLC (DCM:MeOH = 10:1). Overall, 28 mg (24%) of pure product **9g** was obtained. M.p.: 98–102 °C. TLC (DCM:MeOH = 10:1); *R_f_* = 0.42. ^1^H NMR (599.8 MHz; DMSO-*d*_6_) *δ* (ppm): 0.41 (3H; t; *J* = 7.3 Hz; H_3_-18); 0.94 (1H; dq; *J* = 14.1, 7.1 Hz; H_x_-19); 1.46 (1H; dq; *J* = 13.9, 7.2 Hz; H_y_-19); 1.81 (2H; ~hex; *J* = 7.5 Hz; H_2_-3′); 2.21 (2H; ~t; *J* = 7.2 Hz; H_2_-6); 2.43–2.62 (6H; m; N(1)-CH_3_, H_x_-5, H_2_-2′); 2.66 (1H; s; H-21); 2.79 (1H; br d; *J* = 16.4 Hz; H_x_-3); 3.26–3.31 (1H; m; H_y_-5); 3.31–3.39 (1H; m; H_y_-3); 3.52–3.62 (6H; m; H-2, C(16)-COOCH_3_, H_2_-4′); 3.70 (3H; s; C(11)-OCH_3_); 5.07 (1H; br d; *J* = 10.1 Hz; H-15); 5.20 (1H; s; H-17); 5.73 (1H; br dd; *J* = 9.8, 4.7 Hz; H-14); 6.19 (1H; d; *J* = 1.7 Hz; H-12); 6.29 (1H; dd; *J* = 8.2, 1.8 Hz; H-10); 7.06 (1H; d; *J* = 8.2 Hz; H-9); 7.74–7.81 (4H; m; 4×P-Ph: H_meta_); 7.81–7.98 (8H; m; H-4″, H-6″, 4×P-Ph: H_orto_, 2×P-Ph: H_para_); 8.17–8.23 (1H; m; H-5″); 8.87 (1H; s; C(16)-OH); 9.02 (1H; d; *J* = 4.0 Hz; H-3″). ^13^C NMR (150.8 MHz; DMSO-*d*_6_) *δ* (ppm): 7.6 (C-18); 17.8 (d; *J* = 3.2 Hz; C-3′); 19.7 (d; *J* = 50.3 Hz; C-4′); 30.5 (C-19); 33.7 (d; *J* = 17.5 Hz; C-2′); 38.1 (N(1)-CH_3_); 42.4 (C-20); 43.6 (C-6); 50.4 (C-3); 51.1 (C-5); 51.7 (C(16)-COOCH_3_); 52.1 (C-7); 55.1 (C(11)-OCH_3_); 66.1 (C-21); 76.3 (C-17); 78.8 (C-16); 82.7 (C-2); 95.6 (C-12); 104.6 (C-10); 117.4 (2×d; *J* = 85.3; 2×P-Ph: C_ipszo_); 123.2 (C-9); 124.5 (C-14); 125.3 (C-8); 128.5 (d; *J* = 3.2 Hz; C-4″); 129.9 (C-15); 130.3 (2×d; *J* = 12.7 Hz; 4×P-Ph: C_meta_); 131.2 (d; *J* = 24.1 Hz; C-6″); 133.8 (d; *J* = 9.8 Hz; 4×P-Ph: C_orto_); 135.1 (br s; 2×P-Ph: C_para_); 138.4 (d; *J* = 10.3 Hz; C-5″); 144.3 (d; *J* = 116.6 Hz; C-1″); 152.1 (d; *J* = 19.6 Hz; C-3″); 153.4 (C-13); 160.6 (C-11); 171.3 (C-1′); 171.7 (C(16)-COOCH_3_). HRMS: M+ = 746.33534 (delta = −0.01 ppm; C_44_H_49_O_6_N_3_P). HR-ESI-MS-MS (CID = 45%; rel. int. %): 350(100); 332(6); 271(2).

**Preparation of product 10a**

First, 51 mg (0.089 mmol) of 17-(*O*-5-bromopentanoyl)vindoline (**8**) and 162 mg (0.534 mmol, 6.0 eq.) of tri-*p*-tolylphosphine (**5a**) were dissolved in 3 mL of dry acetonitrile. The mixture was stirred at 90 °C under an argon atmosphere for 30 h. The reaction mixture was evaporated under reduced pressure, washed multiple times with *tert*-butyl methyl ether, and filtered through a glass filter. The crude product was purified by preparative TLC (DCM:MeOH = 12:1), yielding 60 mg (77%) of hybrid molecule **10a**. M.p.: 117–119 °C. TLC (DCM:MeOH = 10:1); *R_f_* = 0.37. ^1^H NMR (499.9 MHz; DMSO-*d*_6_) *δ* (ppm): 0.39 (3H; t; *J* = 7.3 Hz; H_3_-18); 0.94 (1H; dq; *J* = 14.2, 7.3 Hz; H_x_-19); 1.37–1.61 (3H; m; H_y_-19, H_2_-4′); 1.68 (2H; qui; *J* = 7.3 Hz; H_2_-3′); 2.16–2.38 (4H; m; H_2_-6, H_2_-2′); 2.44 (9H; s; 3×P-Ph-CH_3_); 2.53–2.61 (4H; m; N(1)-CH_3_, H_x_-5); 2.64 (1H; s; H-21); 2.79 (1H; br d; *J* = 16.3 Hz; H_x_-3); 3.23–3.42 (2H; m; H_y_-3, H_y_-5); 3.43–3.52 (2H; m; H_2_-5′); 3.54 (1H; s; H-2); 3.57 (3H; s; C(16)-COOCH_3_); 3.71 (3H; s; C(11)-OCH_3_); 4.97 (1H; br d; *J* = 10.2 Hz; H-15); 5.17 (1H; s; H-17); 5.75 (1H; ddd; *J* = 10.0, 4.8, 1.4 Hz; H-14); 6.20 (1H; d; *J* = 2.2 Hz; H-12); 6.29 (1H; dd; *J* = 8.2, 2.2 Hz; H-10); 7.06 (1H; d; *J* = 8.2 Hz; H-9); 7.53–7.59 (6H; m; 6×P-Ph: H_meta_); 7.60–7.67 (6H; m; 6×P-Ph: H_orto_); 8.76 (1H; s; C(16)-OH). ^13^C NMR (125.7 MHz; DMSO-*d*_6_) *δ* (ppm): 7.5 (C-18); 20.2 (d; *J* = 51.8 Hz; C-5′); 21.1 (d; *J* = 1.4 Hz; 3×P-Ph-CH_3_); 21.2 (d; *J* = 3.5 Hz; C-4′); 25.1 (d; *J* = 17.9 Hz; C-3′); 30.4 (C-19); 32.6 (C-2′); 37.9 (N(1)-CH_3_); 42.3 (C-20); 43.6 (C-6); 50.4 (C-3); 51.1 (C-5); 51.6 (C(16)-COOCH_3_); 52.0 (C-7); 55.0 (C(11)-OCH_3_); 66.1 (C-21); 75.8 (C-17); 78.6 (C-16); 82.7 (C-2); 95.5 (C-12); 104.5 (C-10); 115.3 (d; *J* = 88.2 Hz; 3×P-Ph: C_ipszo_); 123.1 (C-9); 124.4 (C-14); 125.3 (C-8); 129.7 (C-15); 130.7 (d; *J* = 12.8 Hz; 6×P-Ph: C_meta_); 133.3 (d; *J* = 10.5 Hz; 6×P-Ph: C_orto_); 145.5 (d; *J* = 3.0 Hz; 3×P-Ph: C_para_); 153.3 (C-13); 160.5 (C-11); 171.6 (C(16)-COOCH_3_); 172.2 (C-1′). HRMS: M+ = 801.40043 (delta = −2.8 ppm; C_49_H_58_O_6_N_2_P). HR-ESI-MS-MS (CID = 40%; rel. int. %): 405 (100); 387(7).

**Preparation of product 10b**

First, 51 mg (0.089 mmol) of 17-(*O*-5-bromopentanoyl)vindoline (**8**) and 189 mg (0.534 mmol, 6.0 eq.) of tris(4-methoxyphenyl)phosphine (**5b**) were measured into a pressure-resistant vessel, then dissolved in 3 mL of dry acetonitrile. The mixture was refluxed under an argon atmosphere for 30 h. After the reaction, the solution was evaporated under reduced pressure. The crude product was washed with *tert*-butyl methyl ether and filtered through a glass filter. Finally, it was purified by preparative TLC (DCM:MeOH = 12:1), yielding 64 mg (78%) of product **10b**. M.p.: 115–117 °C. TLC (DCM:MeOH = 10:1); *R_f_* = 0.35. ^1^H NMR (799.7 MHz; DMSO-*d*_6_) *δ* (ppm): 0.39 (3H; t; *J* = 7.3 Hz; H_3_-18); 0.94 (1H; dq; *J* = 14.2, 7.3 Hz; H_x_-19); 1.42 (1H; dq; *J* = 14.2, 7.3 Hz; H_y_-19); 1.46–1.58 (2H; m; H_2_-4′); 1.67 (2H; qui; *J* = 7.4 Hz; H_2_-3′); 2.20 (2H; dd; *J* = 8.9, 6.4 Hz; H_2_-6); 2.24–2.34 (2H; m; H_2_-2′); 2.54–2.59 (4H; m; N(1)-CH_3_, H_x_-5); 2.64 (1H; s; H-21); 2.79 (1H; br d; *J* = 16.7 Hz; H_x_-3); 3.25–3.29 (1H; m; H_y_-5); 3.35–3.44 (3H; m; H_y_-3, H_2_-5′); 3.55 (1H; s; H-2); 3.58 (3H; s; C(16)-COOCH_3_); 3.71 (3H; s; C(11)-OCH_3_); 3.88 (9H; s; 3×P-Ph-OCH_3_); 4.96 (1H; dt; *J* = 10.0, 2.0 Hz; H-15); 5.17 (1H; s; H-17); 5.76 (1H; ddd; *J* = 10.0, 4.9, 1.7 Hz; H-14); 6.19 (1H; d; *J* = 2.3 Hz; H-12); 6.29 (1H; dd; *J* = 8.2, 2.3 Hz; H-10); 7.06 (1H; d; *J* = 8.2 Hz; H-9); 7.26–7.30 (6H; m; 6×P-Ph: H_meta_); 7.63–7.68 (6H; m; 6×P-Ph: H_orto_); 8.77 (1H; s; C(16)-OH). ^13^C NMR (201.1MHz; DMSO-*d*_6_) *δ* (ppm): 7.4 (C-18); 20.8 (d; *J* = 53.2 Hz; C-5′); 21.2 (d; *J* = 3.7 Hz; C-4′); 25.1 (d; *J* = 17.9 Hz; C-3′); 30.4 (C-19); 32.7 (C-2′); 37.9 (N(1)-CH_3_); 42.3 (C-20); 43.6 (C-6); 50.4 (C-3); 51.1 (C-5); 51.6 (C(16)-COOCH_3_); 52.0 (C-7); 55.0 (C(11)-OCH_3_); 55.8 (3×P-Ph-OCH_3_); 66.1 (C-21); 75.8 (C-17); 78.7 (C-16); 82.7 (C-2); 95.5 (C-12); 104.5 (C-10); 109.4 (d; *J* = 93.4 Hz; 3×P-Ph: C_ipszo_); 115.8 (d; *J* = 13.5 Hz; 6×P-Ph: C_meta_); 123.1 (C-9); 124.4 (C-14); 125.3 (C-8); 129.7 (C-15); 135.3 (d; *J* = 11.7 Hz; 6×P-Ph: C_orto_); 153.4 (C-13); 160.4 (C-11); 163.8 (d; *J* = 2.9 Hz; 3×P-Ph: C_para_); 171.6 (C(16)-COOCH_3_); 172.2 (C-1′). HRMS: M+ = 849.38440 (delta = −3.6 ppm; C_49_H_58_O_9_N_2_P). HR-ESI-MS-MS (CID = 40%; rel. int. %): 453(100); 435(7).

**Preparation of product 10c**

First, 51 mg (0.089 mmol) of 17-(*O*-5-bromopentanoyl)vindoline (**8**) and 168 mg (0.534 mmol, 6.0 eq.) of tris(4-fluorophenyl)phosphine (**5c**) were dissolved in 3 mL of dry acetonitrile in a pressure-resistant vessel. The mixture was stirred at reflux temperature under an argon atmosphere. After 23 h, 56 mg (0.178 mmol, 2.0 eq.) of tris(4-fluorophenyl)phosphine (**5c**) was added, followed by another 56 mg (0.178 mmol, 2.0 eq.) after 40 h. Finally, after a total of 47 h of stirring, the reaction mixture was worked up. First, it was evaporated, then washed with *tert*-butyl methyl ether. The filtered crystals were purified by preparative TLC (DCM:MeOH = 12:1). After purification, 35 mg (44%) of the desired **10c** product was obtained. M.p.: 141–143 °C. TLC (DCM:MeOH = 10:1); *R_f_* = 0.38. ^1^H NMR (499.9 MHz; DMSO-*d*_6_) *δ* (ppm): 0.39 (3H; t; *J* = 7.2 Hz; H_3_-18); 0.94 (1H; dq; *J* = 14.2, 7.2 Hz; H_x_-19); 1.42 (1H; dq; *J* = 14.2, 7.3 Hz; H_y_-19); 1.48–1.60 (2H; m; H_2_-4′); 1.68 (2H; qui; *J* = 7.4 Hz; H_2_-3′); 2.17–2.23 (2H; m; H_2_-6); 2.24–2.37 (2H; m; H_2_-2′); 2.52–2.62 (4H; m; N(1)-CH_3_, H_x_-5); 2.64 (1H; s; H-21); 2.79 (1H; br d; *J* = 16.2 Hz; H_x_-3); 3.23–3.43 (2H; m; H_y_-3, H_y_-5); 3.52–3.65 (6H; m; H-2, C(16)-COOCH_3_, H_2_-5′); 3.71 (3H; s; C(11)-OCH_3_); 4.96 (1H; br d; *J* = 10.0 Hz; H-15); 5.17 (1H; s; H-17); 5.76 (1H; ddd; *J* = 10.0, 4.7, 1.2 Hz; H-14); 6.19 (1H; d; *J* = 2.2 Hz; H-12); 6.29 (1H; dd; *J* = 8.2, 2.2 Hz; H-10); 7.06 (1H; d; *J* = 8.2 Hz; H-9); 7.61–7.68 (6H; m; 6×P-Ph: H_meta_); 7.85–7.94 (6H; m; 6×P-Ph: H_orto_); 8.76 (1H; s; C(16)-OH). ^13^C NMR (125.7 MHz; DMSO-*d*_6_) *δ* (ppm): 7.4 (C-18); 20.2 (d; *J* = 50.3 Hz; C-5′); 21.0 (d; *J* = 4.0 Hz; C-4′) 25.1 (d; *J* = 18.4 Hz; C-3′); 30.4 (C-19); 32.6 (C-2′); 37.9 (N(1)-CH_3_); 42.3 (C-20); 43.6 (C-6); 50.4 (C-3); 51.1 (C-5); 51.6 (C(16)-COOCH_3_); 52.0 (C-7); 55.0 (C(11)-OCH_3_); 66.1 (C-21); 75.8 (C-17); 78.6 (C-16); 82.7 (C-2); 95.5 (C-12); 104.5 (C-10); 114.5 (dd; *J* = 90.2, 3.0 Hz; 3×P-Ph: C_ipszo_); 117.8 (dd; *J* = 22.0, 13.8 Hz; 6×P-Ph: C_meta_); 123.1 (C-9); 124.4 (C-14); 125.3 (C-8); 129.7 (C-15); 136.9 (dd; *J* = 12.0, 9.9 Hz; 6×P-Ph: C_orto_); 153.4 (C-13); 160.5 (C-11); 165.9 (dd; *J* = 255.8, 3.4 Hz; 3×P-Ph: C_para_); 171.6 (C(16)-COOCH_3_); 172.2 (C-1′). HRMS: M+ = 813.32385 (delta = −4.5 ppm; C_46_H_49_O_6_N_2_F_3_P). HR-ESI-MS-MS (CID = 40%; rel. int. %): 417(100); 399(8).

**Preparation of product 10d**

First, 51 mg (0.089 mmol) of 17-(*O*-5-bromopentanoyl)vindoline (**8**) and 150 mg (0.534 mmol, 6.0 eq.) of tricyclohexylphosphine (**5d**) were dissolved in 3 mL of dry acetonitrile in a pressure-resistant vessel. The reaction mixture was refluxed under an argon atmosphere for 50 h. Subsequently, the reaction mixture was evaporated under reduced pressure, and the crude product was washed with *tert*-butyl methyl ether. Finally, the crude product was filtered and purified by preparative TLC (DCM:MeOH = 15:1), yielding 56 mg (74%) of hybrid compound **10d**. M.p.: 126–128 °C. TLC (DCM:MeOH = 15:1); *R_f_* = 0.31. ^1^H NMR (799.7 MHz; DMSO-*d*_6_) *δ* (ppm): 0.42 (3H; t; *J* = 7.3 Hz; H_3_-18); 0.98 (1H; dq; *J* = 14.3, 7.3 Hz; H_x_-19); 1.25–1.40 (9H; m; 3×H_x_-3′, 3×H_x_-4′, 3×H_x_-5′); 1.43–1.52 (7H; m; H_y_-19, 3×H_x_-2′, 3×H_x_-6′); 1.53–1.93 (19H; m; 3×H_y_-2′, 3×H_y_-3′, 3×H_y_-4′, 3×H_y_-5′, 3×H_y_-6′, H_2_-3″, H_2_-4″); 2.19–2.23 (2H; m; H_2_-6); 2.23–2.33 (3H; m; H_x_-2″, H_2_-5″); 2.33–2.40 (1H; m; H_y_-2″); 2.46–2.54 (3H; m; 3×H-1′); 2.55–2.60 (4H; m; N(1)-CH_3_, H_x_-5); 2.67 (1H; s; H-21); 2.81 (1H; br d; *J* = 16.5 Hz; H_x_-3); 3.25–3.30 (1H; m; H_y_-5); 3.42 (1H; br dd; *J* = 16.3, 5.0 Hz; H_y_-3); 3.57 (H-2); 3.66 (3H; s; C(16)-COOCH_3_); 3.71 (3H; s; C(11)-OCH_3_); 5.06 (1H; br d; *J* = 10.2 Hz; H-15); 5.21 (1H; s; H-17); 5.83 (1H; ddd; *J* = 10.2, 4.9, 1.6 Hz; H-14); 6.18 (1H; d; *J* = 2.2 Hz; H-12); 6.29 (1H; dd; *J* = 8.2, 2.2 Hz; H-10); 7.06 (1H; d; *J* = 8.2 Hz; H-9); 8.78 (1H; br s; C(16)-OH). ^13^C NMR (201.1 MHz; DMSO-*d*_6_) *δ* (ppm): 7.5 (C-18); 13.9 (d; *J* = 43.0 Hz; C-5″); 20.9 (d; *J* = 4.5 Hz; C-4″); 24.8 (3×C-4′); 25.7–25.9 (3×C-2′, 3×C-3′, 3×C-5′, 3×C-6′, C-3″); 28.4 (d; *J* = 41.2 Hz; 3×C-1′); 30.4 (C-19); 32.5 (C-2″); 37.9 (N(1)-CH_3_); 42.4 (C-20); 43.6 (C-6); 50.4 (C-3); 51.1 (C-5); 51.7 (C(16)-COOCH_3_); 52.0 (C-7); 55.0 (C(11)-OCH_3_); 66.0 (C-21); 75.8 (C-17); 78.6 (C-16); 82.7 (C-2); 95.4 (C-12); 104.4 (C-10); 123.1 (C-9); 124.5 (C-14); 125.3 (C-8); 129.8 (C-15); 153.4 (C-13); 160.4 (C-11); 171.6 (C(16)-COOCH_3_); 172.3 (C-1′). HRMS: M+ = 777.49393 (delta = −3.4 ppm; C_46_H_70_O_6_N_2_P). HR-ESI-MS-MS (CID = 40%; rel. int. %): 381(100); 363(8); 299(3).

**Preparation of product 12**

First, 75 mg (0.132 mmol) of 17-(*O*-4-(chloromethyl)benzoyl)vindoline (**11**) and 172 mg (0.656 mmol, 5.0 eq.) of triphenylphosphine (**1**) were dissolved in 4.5 mL of dry acetonitrile. The reaction mixture was refluxed under an argon atmosphere for 11 h. Due to solvent loss, an additional 2 mL of dry acetonitrile was added to the solution. After the reaction was complete, the mixture was evaporated under reduced pressure. Finally, the crude product was purified by preparative TLC (DCM:MeOH = 8:1), yielding 47 mg (43%) of hybrid molecule **12**. M.p.: 196–198 °C. TLC (DCM:MeOH = 8:1); *R_f_* = 0.33. ^1^H NMR (499.9 MHz; DMSO-*d*_6_): *δ* (ppm) 0.43 (3H; t; *J* = 7.3 Hz; H_3_-18); 1.04 (1H; dq; *J* = 14.1, 7.3 Hz; H_x_-19); 1.57 (1H; dq; *J* = 14.1, 7.3 Hz; H_y_-19); 2.21–2.29 (2H; m; H_2_-6); 2.55–2.64 (4H; N(1)-CH_3_, H_x_-5); 2.69 (1H; s; H-21); 2.82 (1H; br d; *J* = 16.8 Hz; H_x_-3); 3.27–3.37 (1H; m; H_y_-5); 3.45 (1H; br dd; *J* = 16.2, 4.3 Hz; H_y_-3); 3.53 (3H; s; C(16)-COOCH_3_); 3.62 (1H; s; H-2); 3.72 (3H; s; C(11)-OCH_3_); 5.03 (1H; br d; *J* = 10.1 Hz; H-15); 5.29 (2H; d; *J* = 16.0 Hz; H_2_-8′); 5.39 (1H; s; H-17); 5.80 (1H; br dd; *J* = 10.3, 4.4 Hz; H-14); 6.22 (1H; d; *J* = 2.1 Hz; H-12); 6.31 (1H; dd; *J* = 8.2, 2.1 Hz; H-10); 7.06–7.15 (3H; m; H-9, H-4′, H-6′); 7.65–7.78 (14H; m; H-3′, H-7′, 6×P-Ph: H_orto_, 6×P-Ph: H_meta_); 7.89–7.95 (3H; m; 3×P-Ph: H_para_); 8.93 (1H; s; C(16)-OH). ^13^C NMR (125.7 MHz; DMSO-*d*_6_): *δ* (ppm) 7.5 (C-18); 28.0 (d; *J* = 46.7 Hz; C-8′); 30.5 (C-19); 38.0 (N(1)-CH_3_); 42.6 (C-20); 43.5 (C-6); 50.4 (C-3); 51.2 (C-5); 51.7 (C(16)-COOCH_3_); 52.0 (C-7); 55.0 (C(11)-OCH_3_); 66.2 (C-21); 77.0 (C-17); 78.7 (C-16); 82.6 (C-2); 95.6 (C-12); 104.6 (C-10); 117.5 (d; *J* = 85.7 Hz; 3×P-Ph: C_ipszo_); 123.1 (C-9); 124.7 (C-14); 125.3 (C-8); 129.4 (C-3′, C-7′); 129.5 (C-15); 129.6 (d; *J* = 3.4 Hz; C-2′); 130.1 (d; *J* = 12.5 Hz; 6×P-Ph: C_meta_); 131.1 (d; *J* = 5.2 Hz; C-4′, C-6′); 133.6 (d; *J* = 8.5 Hz; C-5′); 133.9 (d; *J* = 9.9 Hz; 6×P-Ph: C_orto_); 135.1 (3× P-Ph: C_para_); 153.4 (C-13); 160.5 (C-11); 164.9 (C-1′); 171.6 (C(16)-COOCH_3_). HRMS: M+H = 793.34007 (delta = −0.04 ppm; C_49_H_50_O_6_N_2_P). HR-ESI-MS-MS (CID = 40%; rel. int. %): 397(100).

**Preparation of product 16**

First, 120 mg (0.219 mmol) of 10-chloroacetamidovindoline (**15**) was dissolved in 7 mL of dry acetonitrile. Then, 145 mg (0.553 mmol, 2.5 eq.) of triphenylphosphine (**1**) was added. The mixture was stirred at room temperature for 22 h, followed by an additional 4 h at 50 °C under an argon atmosphere. The reaction mixture was then evaporated and purified by preparative TLC (DCM:MeOH = 10:1). Finally, 80 mg (45%) of product **16** was isolated. M.p.: 202–203 °C. TLC (DCM:MeOH = 10:1); *R_f_* = 0.36. ^1^H NMR (599.8 MHz; DMSO-*d*_6_) *δ* (ppm): 0.32 (3H; t; *J* = 7.3 Hz; H_3_-18); 0.86 (1H; dq; *J* = 14.0, 7.1 Hz; H_x_-19); 1.43 (1H; dq; *J* = 14.3, 7.2 Hz; H_y_-19); 1.93 (3H; s; C(17)-OCOCH_3_); 2.01–2.10 (1H; m; H_x_-6); 2.13–2.24 (1H; m; H_y_-6); 2.47–2.56 (2H; m; H_x_-5, H-21); 2.58 (3H; s; N(1)-CH_3_); 2.82 (1H; br d; *J* = 16.3 Hz; H_x_-3); 3.27 (1H; td; *J* = 9.2, 3.7 Hz; H_y_-5); 3.40 (1H; br dd; *J* = 16.6, 4.6 Hz; H_y_-3); 3.52 (1H; s; H-2); 3.64 (3H; s; C(16)-COOCH_3_); 3.77 (3H; s; C(11)-OCH_3_); 5.07 (1H; br d; *J* = 10.1 Hz; H-15); 5.11–5.24 (3H; m; H-17, H_2_-2′); 5.82 (1H; br dd; *J* = 10.2, 4.8 Hz; H-14); 6.37 (1H; s; H-12); 7.09 (1H; s; H-9); 7.72–7.78 (6H; m; 6× P-Ph: H_meta_); 7.78–7.85 (6H; m; 6×P-Ph: H_orto_); 7.88 (3H; br t; *J* = 7.3 Hz; 3×P-Ph: H_para_); 8.77 (1H; s; C(16)-OH); 9.68 (1H; s; C(10)-NH-C(1′)). ^13^C NMR (150.8 MHz; DMSO-*d*_6_) *δ* (ppm): 7.5 (C-18); 20.7 (C(17)-OCOCH_3_); 30.4 (C-19); 31.8 (d; *J* = 57.0 Hz; C-2′); 38.4 (N(1)-CH_3_); 42.4 (C-20); 43.5 (C-6); 50.3 (C-3); 51.0 (C-5); 51.7 (C(16)-COOCH_3_); 52.3 (C-7); 55.9 (C(11)-OCH_3_); 65.9 (C-21); 75.8 (C-17); 78.7 (C-16); 82.6 (C-2); 93.7 (C-12); 117.4 (C-9, C-10); 118.8 (br; 3×P-Ph: C_ipszo_); 123.4 (C-8); 124.5 (C-14); 129.8 (C-15); 130.0 (d; *J* = 12.7 Hz; 6×P-Ph: C_meta_); 133.8 (d; *J* = 10.6 Hz; 6× P-Ph: C_orto_); 134.9 (br; 3× P-Ph: C_para_); 148.8 (C-13); 149.8 (C-11); 170.0 (C(17)-OCOCH_3_); 171.5 (C(16)-COOCH_3_). ESI-HRMS: calcd for C_45_H_49_O_7_N_3_P [M+H]+: 774.33026; found: 774.32933; delta = −1.2 ppm.

#### 3.2.3. Synthesis of 17-(O-3,3,3-Triphenylpropanoyl)vindoline (13)

First, 257 mg (0.856 mmol, 1.2 equiv.) of 3,3,3-triphenylpropanoic acid was measured into a pressure-resistant vessel and dissolved in 5 mL of dry DCM. Then, 3.72 mL (51.3 mmol, 60 eq.) of thionyl chloride and 3 drops of DMF were added. The mixture was stirred at 55 °C under an argon atmosphere for 3 h. Finally, the mixture was evaporated and used in the next reaction without purification. First, solution A was prepared: the 3,3,3-triphenylpropanoyl chloride was dissolved in 3 mL of dry DCM. Next, solution B was prepared: 299 mg (0.721 mmol) of 17-deacetylvindoline (**6**) was dissolved in 5 mL of dry DCM and 0.24 mL (1.708 mmol, 2.4 eq.) of triethylamine was added. After preparing the solutions, solution A was added dropwise to solution B at 0 °C, followed by stirring at room temperature for 14 h. Since the reaction did not reach full conversion, the stirring was continued for an additional 1 h at 45 °C. Once the reaction was complete, 5 mL of DCM and 7 mL of water were added to the mixture and stirred for another 30 min. After phase separation, the water phase was extracted with 3 × 15 mL DCM. The organic phases were combined and dried over MgSO_4_. Finally, the solution was evaporated and washed with *tert*-butyl methyl ether. The crude product was first purified by column chromatography (DCM:MeOH = 30:1) and then further purified by preparative TLC (DCM:MeOH = 20:1). Ultimately, 181 mg (36%) of product **13** was isolated. M.p.: 99–101 °C. TLC (DCM:MeOH = 10:1); *R_f_* = 0.25. ^1^H NMR (599.8 MHz; DMSO-*d*_6_) *δ* (ppm): 0.27 (3H; t; *J* = 7.3 Hz; H_3_-18); 0.65 (1H; dq; *J* = 14.1, 7.0 Hz; H_x_-19); 0.82 (1H; dq; *J* = 13.9, 7.2 Hz; H_y_-19); 2.06–2.20 (2H; m; H_2_-6); 2.50–2.55 (1H; m; H_x_-5); 2.56 (3H; s; N(1)-CH_3_); 2.59 (1H; s; H-21); 2.71 (1H; br d; *J* = 16.4 Hz; H_x_-3); 3.23 (1H; td; *J* = 9.1, 4.4 Hz; H_y_-5); 3.29–3.36 (2H; m; H_y_-3; H_x_-2′); 3.50 (1H; s; H-2); 3.67 (3H; s; C(11)-OCH_3_); 3.70 (3H; s; C(16)-COOCH_3_); 4.07 (1H; d; *J* = 17.1 Hz; H_y_-2′); 4.37 (1H; br d; *J* = 10.6 Hz; H-15); 4.96 (1H; s; H-17); 5.64 (1H; br dd; *J* = 10.4, 5.1 Hz; H-14); 6.12 (1H; d; *J* = 1.8 Hz; H-12); 6.22 (1H; dd; *J* = 8.2, 1.8 Hz; H-10); 6.96 (1H; d; *J* = 7.6 Hz; H-9); 7.13–7.28 (15H; m; 6×C(3′)-Ph: H_orto_, 6×C(3′)-Ph: H_meta_, 3×C(3′)-Ph: H_para_); 8.68 (1H; s; C(16)-OH). ^13^C NMR (150.8 MHz; DMSO-*d*_6_) *δ* (ppm): 7.2 (C-18); 29.6 (C-19); 37.9 (N(1)-CH_3_); 42.0 (C-20); 43.6 (C-6); 44.9 (C-2′); 50.1 (C-3); 50.6 (C-5); 51.8 (C(16)-COOCH_3_); 52.1 (C-7); 54.7 (C-3′); 54.9 (C(11)-OCH_3_); 65.6 (C-21); 75.4 (C-17); 78.8 (C-16); 82.7 (C-2); 95.2 (C-12); 104.2 (C-10); 122.9 (C-9); 123.7 (C-14); 124.9 (C-8); 125.8 (3×C(3′)-Ph: C_para_); 127.5 (6×C(3′)-Ph: C_meta_); 128.8 (6×C(3′)-Ph: C_orto_); 129.9 (C-15); 146.4 (3×C(3′)-Ph: C_ipszo_); 153.2 (C-13); 160.4 (C-11); 169.4 (C-1′); 171.4 (C(16)-COOCH_3_). ESI-HRMS: calcd for C_44_H_47_O_6_N_2_ [M+H]+: 699.34286; found: 699.34203; delta = −1.2 ppm.

### 3.3. Biological Evaluation

#### 3.3.1. NCI60 Screening

A detailed description of the NCI screening procedures [43,44,45,46,47], including the one-dose and five-dose tests, can be found in the Supplementary Materials, on the website of NCI [48], and in our previous work [40,42].

#### 3.3.2. Determination of Antiproliferative Activities on Adherent Cell Lines

The antiproliferative effects of the prepared derivatives were evaluated in vitro using the MTT assay against a panel of human adherent cancer cell lines, including breast (MDA-MB-231, MCF-7), cervical (SiHa, HeLa), and ovarian (A2780) cancer cells [49]. The noncancerous fibroblast cell line NIH/3T3 was included to obtain data concerning the tumor selectivity of the test substances. All cell lines were sourced from the European Collection of Cell Cultures (Salisbury, UK), except for SiHa (American Tissue Culture Collection (Manassas, VA, USA). All cell lines were maintained in Eagle’s Minimal Essential Medium (EMEM) supplemented with 10% fetal bovine serum (FBS), 1% non-essential amino acids, and a mixture of penicillin, streptomycin, and amphotericin B. The cells were maintained in a humidified atmosphere containing 5% CO_2_ at 37 °C. The media and the cell culture supplements were procured from Capricorn Scientific Ltd. (Ebsdorfergrund, Germany). Cells were seeded into 96-well microplates at 5000/well and, the next day, treated with vindoline derivatives at two concentrations (10 or 30 μM) for 72 h under cell culturing conditions. After the incubation period, 20 µL of 5 mg/mL MTT solution [(3-(4,5-dimethylthiazol-2-yl)-2,5-diphenyltetrazolium bromide] was added for four hours. The culture medium was removed, the precipitated formazan was dissolved in dimethyl sulfoxide (DMSO), and the absorbance of this solution was then measured at 545 nm. Untreated cells served as a control group and cisplatin was included as a reference compound across similar concentration ranges. The 10 mM stock solutions of the tested compounds were prepared using dimethyl sulfoxide (DMSO), and the highest concentration of DMSO in the medium (0.3%) did not significantly affect cell proliferation. When higher than 50% cell growth inhibition was observed, the assay was repeated using a wide concentration range (0.1–30 μM). The *IC*_50_ values were calculated using GraphPad Prism software 10.3.1 (GraphPad Software, San Diego, CA, USA). Two independent experiments were performed with at least five replicates per condition.

#### 3.3.3. Cell Cycle Analysis

A2780 cells were seeded onto 12-well plates at densities of 200,000 cells per well and the plates were then incubated overnight under standard cell culture conditions. Then, the media were removed and the cells in each well were washed with phosphate-buffered saline (PBS) and treated with 1000 µL of fresh medium containing 1.5 or 3.0 µM of compound **9g** for 24 or 48 h. After the incubation, the cells were washed with PBS and harvested using trypsinization. The cell suspensions were centrifuged at 1100 rpm for 5 min at room temperature. The cells were rewashed and fixed in 500 µL of cold 70% ethanol for 30 min. Following the fixation, cells were centrifuged at 1300 rpm for 5 min at 4 °C and the pellets were resuspended in 300 µL staining solution (containing 10 µg/mL propidium iodide, 0.1% Triton-X, 0.1% sodium citrate, and 10 µg/mL RNase-A in water). The samples were stored in the dark at room temperature for 30 min. The stained cells were then analyzed using a flow cytometer (CytoFLEX, Beckman Coulter, Brea, CA, USA), recorded by CytExpert Software 2.5.0.77 (Beckman Coulter), ensuring that at least 20,000 events per sample were evaluated. The percentages of cells in different phases of the cell cycle (subG1, G1, S, and G2/M) were determined using ModFit LT 3.3.11 software (Verity Software House, Topsham, ME, USA); the subG1 fraction indicated the apoptotic cell population, as referenced by Vermes et al. [50]. Untreated cells were consistently used as a control throughout the experiments; the entire cell cycle analysis experiment was performed independently three times, and each time, three parallel samples for each condition were used to ensure reproducibility and reliability of results.

#### 3.3.4. Migration Assay

The effect of compound **9g** on the migration of A2780 cells was assessed using a wound-healing assay. A2780 cells were trypsinized and seeded into specialized wound assay chambers (ibidi GmbH, Martinsried, Germany) at a density of 80,000 cells/insert and the plate was incubated under standard conditions to allow proper attachment. After incubation, the inserts were carefully removed and the wells were washed twice with PBS to eliminate non-adherent cells and debris. The cells were subsequently treated with compound **15** at 0.5 or 1.0 µM in fresh medium containing 2% FBS. The migration of cells toward the wound site was visualized using a phase-contrast inverted microscope (Nikon Instruments Europe, Amstelveen, The Netherlands) and images were captured with a CCD camera (QImaging MicroPublisher Color RTV5.0 Teledyne Photometrics, Tucson, AZ, USA). The percentage of cell migration was calculated at different time intervals (0, 24, and 48 h) by measuring the cell-free area in treated samples against untreated controls using ImageJ software 1.54d (National Institutes of Health, Bethesda, MD, USA). The experiments were conducted twice with at least three parallel samples.

#### 3.3.5. CellTiter-Glo Luminescent Cell Viability Assay on Non-Tumor CHO Cells

Conjugates **9b** and **9e** were dissolved in DMSO in 10 mM stock solutions and stored frozen until use. CHO cells were cultured in complete Dulbecco’s Modified Eagle’s Medium (DMEM, low glucose (1 g/L) supplemented with 10% fetal bovine serum, 1% Gibco GlutaMAX-I (100×) solution, 1% Gibco MEM non-essential amino acid solution (100×), and 0.1% penicillin–streptomycin mixture). The CellTiter-Glo Luminescent Cell Viability Assay (Promega Corporation, Madison, WI, USA) was performed as previously described [42,51,52]. Cells were grown under standard cell culture conditions (37 °C, 5% CO_2_) and passaged at 70–80% confluency. According to the manufacturer’s protocol, CHO cells were seeded to opaque 96-well culture plates (5000 cells/100 µL complete DMEM/well) and the side rows and columns of the plate were filled with sterile phosphate-buffer saline to avoid artifacts. Following overnight incubation (37 °C, 5% CO_2_), the culture medium was aspirated from the cells and adherent cells were treated with increasing concentrations of drug solutions (10, 1.0, and 0.10 µM; 100 µL/well) diluted from the stock solution in sterile complete DMEM. Untreated cells served as a control, and complete DMEM as a luminescent background control. Following a 48 h incubation (37 °C, 5% CO_2_), assay plates were equilibrated to room temperature and 100 µL of room temperature CellTiter-Glo reagent was added to each well. Plates were placed on an orbital shaker for 2 min and incubated for 10 min at room temperature without shaking. The luminescent signal was measured for each assay well with an EnSpire AlphaLisa microplate reader (Perkin Elmer, Inc., Waltham, MA, USA). Relative light unit (RLU) values were normalized and values of the treated wells were compared to the untreated control, which was considered 100% viability. Statistical analysis and estimation of *IC*_50_ values were performed in GraphPad Prism 8.0.1 (GraphPad, La Jolla, CA, USA). Estimated *IC*_50_ values were calculated using non-linear regression by fitting a sigmoidal dose-response curve to the data points (log(inhibitor) vs. normalized response, variable slope, and least squares fit).

#### 3.3.6. Statistical Analysis

Statistical analysis (ANOVA) and estimation of *IC*_50_ values were performed in GraphPad Prism 8.0.1 (GraphPad, La Jolla, CA, USA). Estimated *IC*_50_ values were calculated using non-linear regression by fitting a sigmoidal dose-response curve to the data points (log(inhibitor) vs. normalized response, variable slope, least squares fit).

## 4. Conclusions

This study is an extension of our previous work [35,36]. It has recently been revealed that coupling the *Vinca* alkaloid vindoline, which is ineffective by itself, with TPP leads to phosphonium salts with encouraging antitumor activity. The phosphonium-type ionic building block is beneficial because it allows drugs to pass through the cell membrane. Thus, the compounds coupled to it can exert their activities in the mitochondria. Continuing the work, the question of how the anticancer effect changes when differently substituted TPP derivatives are used instead of the simple TPP arose. Furthermore, we aimed to examine the selectivity of the most active cytotoxic agents using non-tumor cells. We synthesized 13 vindoline–TPP hybrids by simple transformations. In these, we varied the length and rigidity of the linker, incorporated variously substituted TPP derivatives, and formed a hybrid not only at position 17 of vindoline but also at position 10. Furthermore, we prepared a derivative (**13**) that contained a trityl group instead of the ionic triphenylphosphonium unit. The latter was found to have no cytotoxic effect, which was also true for the derivative **16** linked at position 10. Among the hybrids, the TPP salts substituted with methyl and methoxy groups (**9a**, **9b**, **9e**, **10a**, and **10b**) proved the most effective, outperforming the two previously prepared reference molecules (**3** and **4**). Among them, the tris(4-methoxy-3,5-dimethylphenyl)phosphine derivative **9e** was the most potent, showing a *GI*_50_ value of 20.0 nM in the case of leukemia on the RPMI-8226 cell line. Compound **9g** exerted outstanding antiproliferative activity against human adherent cancer cell lines with considerable tumor selectivity. This compound also elicited profound cell cycle disturbance, cumulation of hypodiploid cells, and inhibition of the migration of A2780 ovarian cancer cells. The CellTiter-Glo Luminescent Cell Viability Assay (Promega Corporation, Madison, WI, USA) of compounds **9b** and **9e** on non-tumor CHO cells revealed significant selectivity for cancer cells. Finally, we would like to emphasize that this study may significantly impact the design of novel *Vinca* alkaloid-based anticancer agents.

## Figures and Tables

**Table 1:** *GI*_50_ values (µM) of compounds **3** and **9a**–**f** against 60 human cancer cell lines in vitro. Values where *GI*_50_ < 1 µM are highlighted in bold. n.d. means not determined.

		3		9a		9b		9c		9d		9e		9f	
Panel	Cell Line	GI_50_	Mean	GI_50_	Mean	GI_50_	Mean	GI_50_	Mean	GI_50_	Mean	GI_50_	Mean	GI_50_	Mean
Leukemia	CCRF-CEM	**0.487**	**0.36**	**0.359**	**0.26**	**0.887**	**0.55**	12.70	7.25	1.690	1.20	**0.114**	**0.11**	**0.256**	**0.22**
	HL-60(TB)	**0.124**		**0.162**		**0.256**		1.770		**0.322**		**0.039**		**0.170**	
	K-562	**0.360**		**0.291**		**0.556**		9.830		1.320		**0.195**		**0.125**	
	MOLT-4	**0.614**		**0.294**		**0.933**		9.690		2.160		**0.164**		**0.419**	
	RPMI-8226	**0.219**		**0.186**		**0.275**		2.050		**0.383**		**0.020**		**0.106**	
	SR	**0.346**		**0.281**		**0.389**		7.450		1.350		**0.144**		**0.221**	
Non-small Cell Lung Cancer	A549/ATCC	1.350	1.11	**0.334**	**0.41**	**0.731**	1.09	21.50	19.58	3.460	3.67	**0.275**	**0.23**	**0.945**	1.05
	EKVX	**0.470**		**0.534**		1.060		19.40		3.090		n.d.		1.220	
	HOP-62	1.370		**0.470**		1.660		20.50		3.680		**0.173**		2.160	
	HOP-92	**0.066**		**0.187**		**0.344**		5.440		1.010		**0.201**		**0.172**	
	NCI-H226	2.040		**0.521**		1.430		24.20		4.060		**0.230**		1.210	
	NCI-H23	**0.971**		**0.303**		**0.671**		15.80		2.310		**0.183**		**0.413**	
	NCI-H322M	2.330		**0.785**		2.860		>48.5		5.790		**0.342**		2.230	
	NCI-H460	1.220		**0.327**		**0.666**		17.00		2.770		**0.229**		**0.774**	
	NCI-H522	**0.216**		**0.258**		**0.391**		3.880		6.880		**0.198**		**0.339**	
Colon Cancer	COLO 205	**0.161**	1.69	**0.176**	**0.51**	**0.194**	**0.98**	4.980	16.80	**0.430**	3.82	**0.091**	**0.24**	**0.179**	1.61
	HCC-2998	**0.436**		**0.415**		**0.498**		9.530		1.700		**0.195**		**0.325**	
	HCT-116	**0.965**		**0.307**		**0.712**		14.80		2.850		**0.223**		**0.383**	
	HCT-15	8.500		1.620		3.640		>48.5		15.30		**0.760**		9.070	
	HT29	**0.266**		**0.219**		**0.371**		12.50		1.880		**0.151**		**0.356**	
	KM12	**0.297**		**0.376**		**0.628**		8.960		1.630		**0.047**		**0.331**	
	SW-620	1.200		**0.434**		**0.844**		18.30		2.950		**0.245**		**0.637**	
CNS Cancer	SF-268	**0.975**	**0.76**	**0.343**	**0.36**	1.210	1.02	15.00	20.21	3.230	2.71	**0.231**	**0.18**	**0.372**	**0.57**
	SF-295	1.450		**0.521**		1.920		>48.5		4.410		**0.213**		1.830	
	SF-539	**0.662**		**0.338**		1.230		16.80		2.940		**0.307**		**0.434**	
	SNB-19	**0.334**		**0.306**		**0.391**		11.40		2.220		**0.059**		**0.313**	
	SNB-75	**0.750**		n.d.		n.d.		n.d.		n.d.		**0.195**		**0.197**	
	U251	**0.371**		**0.291**		**0.360**		9.360		**0.746**		**0.054**		**0.248**	
Melanoma	LOX IMVI	**0.858**	**0.45**	**0.333**	**0.35**	**0.614**	**0.76**	16.60	8.13	2.880	1.70	**0.145**	**0.18**	**0.429**	**0.49**
	MALME-3M	**0.283**		**0.220**		**0.624**		6.850		1.460		**0.288**		**0.295**	
	M14	**0.790**		**0.456**		1.120		9.100		1.920		**0.299**		1.060	
	MDA-MB-435	**0.278**		**0.331**		**0.480**		8.140		1.830		**0.231**		**0.299**	
	SK-MEL-2	**0.322**		n.d.		n.d.		n.d.		n.d.		**0.060**		**0.322**	
	SK-MEL-28	**0.261**		**0.361**		**0.453**		5.340		1.080		**0.272**		**0.506**	
	SK-MEL-5	**0.766**		**0.532**		1.550		8.260		1.760		**0.138**		**0.777**	
	UACC-257	**0.240**		**0.272**		**0.646**		4.670		1.260		**0.038**		**0.406**	
	UACC-62	**0.222**		**0.275**		**0.603**		6.050		1.410		**0.160**		**0.286**	
Ovarian Cancer	IGROV1	**0.237**	7.78	**0.383**	1.57	**0.533**	3.46	7.090	19.04	1.390	7.12	**0.193**	**0.94**	**0.510**	4.20
	OVCAR-3	**0.369**		**0.374**		**0.624**		6.470		1.740		**0.147**		**0.297**	
	OVCAR-4	**0.469**		**0.370**		**0.760**		16.20		2.390		**0.271**		**0.540**	
	OVCAR-5	1.200		**0.331**		1.770		20.80		3.190		**0.339**		**0.735**	
	OVCAR-8	1.270		**0.372**		1.413		18.40		2.970		**0.300**		**0.790**	
	NCI/ADR-RES	>50.0		8.870		18.10		>48.5		35.60		5.110		26.10	
	SK-OV-3	**0.894**		**0.290**		1.023		15.80		2.590		**0.232**		**0.411**	
Renal Cancer	786-0	2.740	3.52	1.060	1.05	1.930	3.01	>48.5	34.21	4.450	7.93	**0.239**	**0.44**	1.980	3.08
	A498	1.710		1.630		2.344		>48.5		12.40		**0.314**		2.650	
	ACHN	4.530		1.610		**0.458**		>48.5		15.20		**0.319**		4.580	
	CAKI-1	5.570		1.870		13.70		>48.5		16.40		**0.313**		1.900	
	RXF 393	**0.705**		**0.279**		**0.482**		8.460		1.470		n.d.		**0.247**	
	SN12C	**0.590**		**0.291**		**0.672**		14.80		2.890		**0.159**		**0.325**	
	TK-10	1.480		**0.600**		1.514		22.20		2.730		**0.277**		1.940	
	UO-31	10.80		n.d.		n.d.		n.d.		n.d.		1.480		11.00	
Prostate Cancer	PC-3	**0.204**	**0.64**	**0.310**	**0.38**	**0.470**	**0.71**	12.60	18.15	1.710	2.73	**0.192**	**0.25**	**0.268**	**0.34**
	DU-145	1.070		**0.457**		**0.955**		23.70		3.750		**0.300**		**0.418**	
Breast Cancer	MCF7	**0.306**	**0.45**	**0.281**	**0.27**	**0.383**	**0.64**	8.260	8.04	1.190	1.54	**0.044**	**0.14**	**0.288**	**0.31**
	MDA-MB231ATCC	**0.406**		n.d.		n.d.		n.d.		n.d.		**0.293**		**0.397**	
	HS 578T	1.260		**0.344**		1.180		11.80		2.450		**0.185**		**0.473**	
	BT-549	**0.380**		**0.247**		1.050		8.130		2.440		**0.170**		**0.262**	
	T-47D	**0.192**		**0.314**		**0.353**		10.500		1.320		**0.105**		**0.302**	
	MDA-MB-468	**0.126**		**0.165**		**0.241**		1.500		**0.313**		**0.027**		**0.129**	
Mean		1.985		0.605		1.449		17.010		3.799		0.309		1.439	

**Table 2:** *GI*_50_ values (µM) of compounds **4**, **10a**–**d**, and **12** against 60 human cancer cell lines in vitro. Values where *GI*_50_ < 1 µM are highlighted in bold. n.d. means not determined.

		4		10a		10b		10c		10d		12	
Panel	Cell Line	GI_50_	Mean	GI_50_	Mean	GI_50_	Mean	GI_50_	Mean	GI_50_	Mean	GI_50_	Mean
Leukemia	CCRF-CEM	**0.697**	**0.60**	**0.768**	**0.48**	n.d.	**0.56**	13.60	9.86	2.550	1.22	1.300	**0.77**
	HL-60(TB)	n.d.		**0.221**		**0.234**		2.110		**0.293**		**0.297**	
	K-562	n.d.		**0.654**		**0.675**		11.30		**0.983**		**0.448**	
	MOLT-4	n.d.		**0.611**		1.180		14.70		1.720		1.470	
	RPMI-8226	**0.497**		**0.283**		**0.299**		2.970		**0.412**		**0.325**	
	SR	n.d.		**0.360**		**0.428**		14.50		1.390		n.d.	
Non-small Cell Lung Cancer	A549/ATCC	1.020	1.44	**0.819**	1.07	1.650	1.36	33.80	24.32	3.130	2.95	2.090	2.05
	EKVX	**0.586**		1.050		**0.185**		21.00		3.130		2.030	
	HOP-62	2.600		1.370		2.420		25.50		3.900		2.670	
	HOP-92	**0.268**		**0.490**		**0.520**		6.320		1.020		**0.404**	
	NCI-H226	1.930		1.430		2.030		27.30		4.410		1.540	
	NCI-H23	1.220		**0.855**		1.190		20.10		2.340		2.810	
	NCI-H322M	3.000		2.670		2.790		50.00		5.300		4.760	
	NCI-H460	2.110		**0.600**		1.150		29.40		2.710		1.660	
	NCI-H522	**0.229**		**0.306**		**0.341**		5.440		**0.642**		**0.491**	
Colon Cancer	COLO 205	**0.242**	3.19	**0.200**	**0.72**	**0.214**	1.88	4.630	26.90	**0.377**	3.80	**0.313**	4.69
	HCC-2998	**0.367**		**0.520**		**0.617**		12.50		1.310		**0.991**	
	HCT-116	1.060		**0.452**		1.300		25.90		2.940		1.590	
	HCT-15	18.60		2.170		8.790		>100		16.10		27.40	
	HT29	**0.404**		**0.358**		**0.511**		15.40		1.430		**0.541**	
	KM12	**0.442**		**0.596**		**0.675**		13.40		1.720		**0.514**	
	SW-620	1.180		**0.761**		1.060		16.50		2.750		1.500	
CNS Cancer	SF-268	**0.634**	**0.94**	1.170	1.09	1.470	1.17	17.60	18.76	3.170	2.60	1.280	1.32
	SF-295	2.750		1.620		1.970		30.60		4.400		2.920	
	SF-539	1.300		1.220		1.520		21.90		2.850		1.260	
	SNB-19	**0.397**		**0.960**		**0.484**		13.00		1.920		**0.725**	
	SNB-75	**0.221**		n.d.		n.d.		n.d.		n.d.		1.110	
	U251	**0.312**		**0.470**		**0.392**		10.70		**0.640**		**0.644**	
Melanoma	LOX IMVI	1.170	**0.75**	**0.797**	**0.92**	1.110	**0.98**	16.80	12.52	2.900	1.96	1.820	1.03
	MALME-3M	**0.273**		1.170		1.130		13.50		1.630		**0.652**	
	M14	1.790		1.110		1.200		14.70		2.200		1.350	
	MDA-MB-435	**0.357**		**0.678**		**0.521**		12.90		2.620		**0.483**	
	SK-MEL-2	**0.279**		n.d.		n.d.		n.d.		n.d.		1.270	
	SK-MEL-28	**0.532**		**0.485**		**0.486**		8.280		1.380		**0.520**	
	SK-MEL-5	1.710		1.570		1.600		17.10		1.880		1.210	
	UACC-257	**0.215**		**0.817**		**0.792**		5.590		1.570		**0.918**	
	UACC-62	**0.403**		**0.705**		**0.963**		11.30		1.530		1.030	
Ovarian Cancer	IGROV1	**0.553**	15.08	**0.479**	2.54	**0.548**	4.11	10.80	32.46	1.360	11.42	**0.885**	15.79
	OVCAR-3	**0.398**		**0.604**		**0.634**		9.300		1.930		**0.704**	
	OVCAR-4	**0.326**		1.090		1.570		25.10		2.720		1.050	
	OVCAR-5	1.820		1.440		2.130		29.10		3.190		1.990	
	OVCAR-8	1.740		1.130		1.990		29.40		2.930		3.180	
	NCI/ADR-RES	>100		12.00		20.50		>100		65.50		>100	
	SK-OV-3	**0.725**		1.040		1.400		23.50		2.280		2.700	
Renal Cancer	786-0	2.900	4.87	1.640	2.34	2.590	4.34	34.10	56.74	5.430	8.36	3.580	8.30
	A498	2.840		2.170		2.930		>100		13.30		16.40	
	ACHN	14.60		2.540		6.280		>100		16.00		18.50	
	CAKI-1	2.820		7.460		15.20		>100		16.70		3.260	
	RXF 393	**0.308**		**0.468**		**0.524**		13.30		1.240		1.140	
	SN12C	**0.467**		**0.619**		1.180		28.60		2.770		**0.708**	
	TK-10	2.820		1.490		1.670		21.20		3.080		2.840	
	UO-31	12.20		n.d.		n.d.		n.d.		n.d.		20.00	
Prostate Cancer	PC-3	**0.369**	**0.57**	**0.450**	**0.80**	**0.562**	8.28	16.40	25.90	1.460	2.56	**0.657**	1.13
	DU-145	**0.763**		1.150		16.00		35.40		3.650		1.610	
Breast Cancer	MCF7	**0.322**	**0.50**	**0.325**	**0.58**	**0.105**	**0.70**	11.50	11.08	1.050	1.48	**0.442**	**0.61**
	MDA-MB231ATCC	**0.370**		n.d.		n.d.		n.d.		n.d.		**0.686**	
	HS 578T	1.230		1.200		1.510		16.90		2.610		1.270	
	BT-549	**0.583**		**0.763**		1.250		12.90		2.450		**0.538**	
	T-47D	**0.303**		**0.370**		**0.383**		11.50		**0.920**		**0.525**	
	MDA-MB-468	**0.170**		**0.264**		**0.264**		2.610		**0.378**		**0.186**	
Mean		3.525		1.232		2.202		24.856		4.289		4.325	

**Table 3:** The antiproliferative properties of the prepared analogs.

Cell Line	Conc. (μM)	Growth Inhibition (%) ± SEM		
		9g	13	16
HeLa	10 30	61.45 ± 2.35 68.50 ± 2.36	39.86 ± 2.57 40.15 ± 1.12	21.67 ± 0.84 29.96 ± 0.45
SiHa	10 30	62.46 ± 0.87 83.76 ± 3.07	>10 * >10	14.96 ± 0.34 36.78 ± 3.30
MCF-7	10 30	81.94 ± 0.84 89.92 ± 0.72	- 20.82 ± 1.54	31.15 ± 3.10 47.13 ± 0.81
MDA-MB-231	10 30	72.04 ± 0.35 87.71 ± 1.74	>10 >10	12.64 ± 0.03 13.46 ± 0.38
A2780	10 30	79.37 ± 2.63 85.47 ± 2.25	>10 11.90 ± 0.85	19.42 ± 0.64 29.48 ± 0.56
NIH/3T3 (fibroblast)	10 30	34.63 ± 1.11 77.24 ± 2.35	26.12 ± 1.55 22.14 ± 0.67	24.95 ± 0.78 27.48 ± 0.90

*: Growth inhibition values less than 10% are considered negligible and not presented numerically.

**Table 4:** The calculated *IC*_50_ values and tumor selectivity ratios of compound **9g** and cisplatin.

Cell Line	IC_50_ Value (μM) ± SEM	
	Selectivity Ratio	
	9g	Cisplatin
HeLa	6.48 ± 0.12 0.45	13.60 ± 0.44 3.23
SiHa	2.42 ± 0.05 0.17	15.50 ± 0.93 3.68
MCF-7	2.56 ± 0.10 0.18	2.60 ± 0.17 0.62
MDA-MB-231	3.35 ± 0.12 0.23	19.13 ± 0.12 4.54
A2780	1.50 ± 0.20 0.10	1.40 ± 0.11 0.33
NIH/3T3 (fibroblast)	14.37 ± 0.02	2.70 ± 0.30

## Data Availability

Data is contained within the article and Supplementary Materials.

## Supplementary Materials

The following supporting information can be downloaded at: https://www.mdpi.com/article/10.3390/ijms26083775/s1.
