# Supplementary material for: Synthesis and In Vitro Evaluation of the Anticancer Effect of Novel Phosphonium Vindoline Derivatives
Source: Int J Mol Sci. 2025 Apr 16;26(8):3775. doi: 10.3390/ijms26083775 (PMC12028158; doi:10.3390/ijms26083775)
Supplement: Supplementary file 1 [file ijms-26-03775-s001.zip › ijms-3555145-supplementary.pdf]

Supplementary Materials

Synthesis and In Vitro Evaluation of the Anticancer Effect of Novel Phosphonium Vindoline Derivatives

Mónika Halmai [1], Viktória Donkó-Tóth [1], Péter Keglevich [1], Károly Kánai [1], Márton Weber [2], Miklós Dékány [2], Ejlal A. Abdallah [3], Noémi Bózsity [3], István Zupkó [3], Andrea Nehr-Majoros [4,5,6], Éva Szőke [4,5,6], Zsuzsanna Helyes [4,5,6,7] and László Hazai [1,*]

[1] Department of Organic Chemistry and Technology, Faculty of Chemical Technology and Biotechnology, Budapest University of Technology and Economics, Műegyetem rkp. 3, H-1111 Budapest, Hungary
[2] Spectroscopic Research Department, Gedeon Richter Plc., Gyömrői út 19-21, H-1103 Budapest, Hungary
[3] Institute of Pharmacodynamics and Biopharmacy, University of Szeged, Eötvös u. 6, H-6720 Szeged, Hungary
[4] Department of Pharmacology and Pharmacotherapy, Medical School & Centre for Neuroscience, University of Pécs, Szigeti út 12, H-7624 Pécs, Hungary
[5] National Laboratory for Drug Research and Development, Magyar Tudósok krt. 2, H-1117 Budapest, Hungary
[6] HUN-REN PTE Chronic Pain Research Group, Szigeti út 12, H-7624 Pécs, Hungary
[7] PharmInVivo Ltd., Szondi Gy. u. 10, H-7629 Pécs, Hungary
* Correspondence: hazai.laszlo@vbk.bme.hu

S1. ^{1}H NMR, ^{13}C NMR and HRMS spectra of all the newly synthesized compounds (9a–g, 10a–d, 11–13 and 16)

S1.1. Product 9a

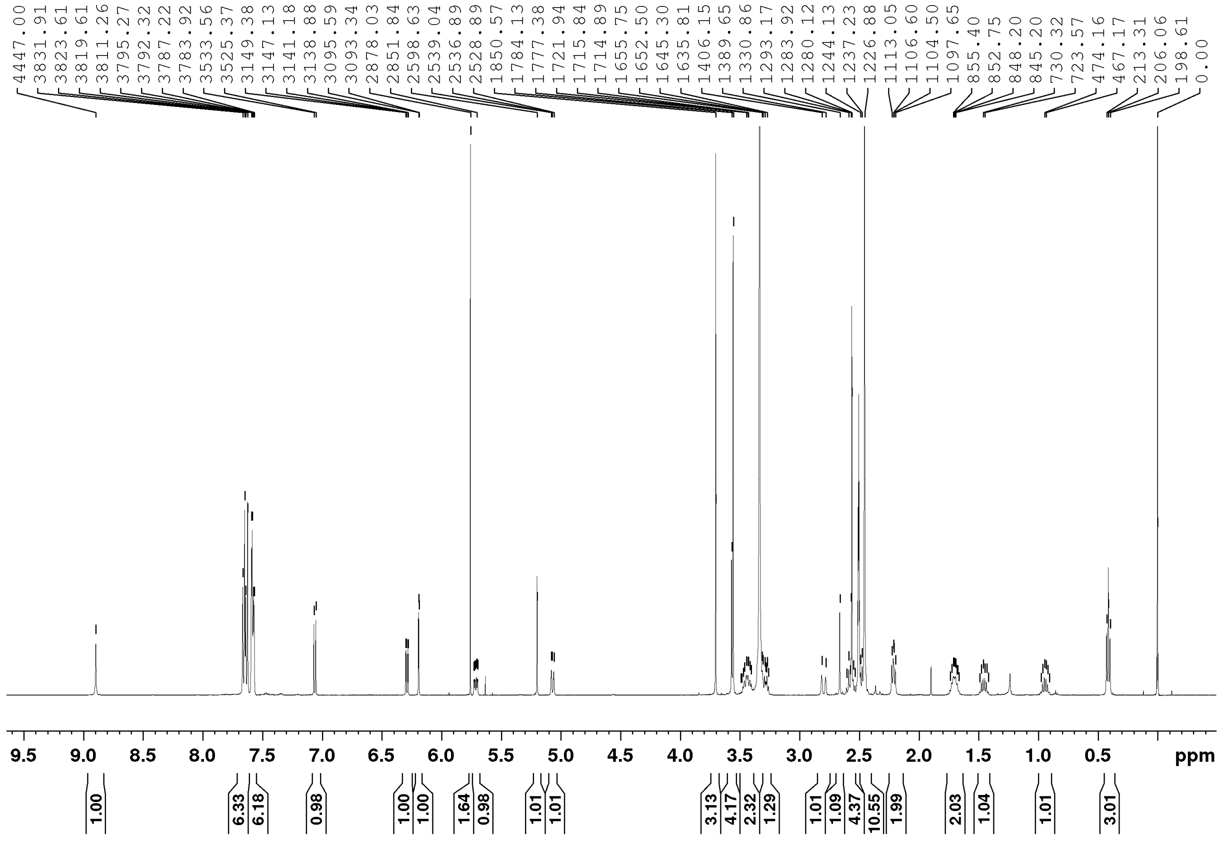

Figure S1. ^{1}H NMR spectrum of compound **9a**.

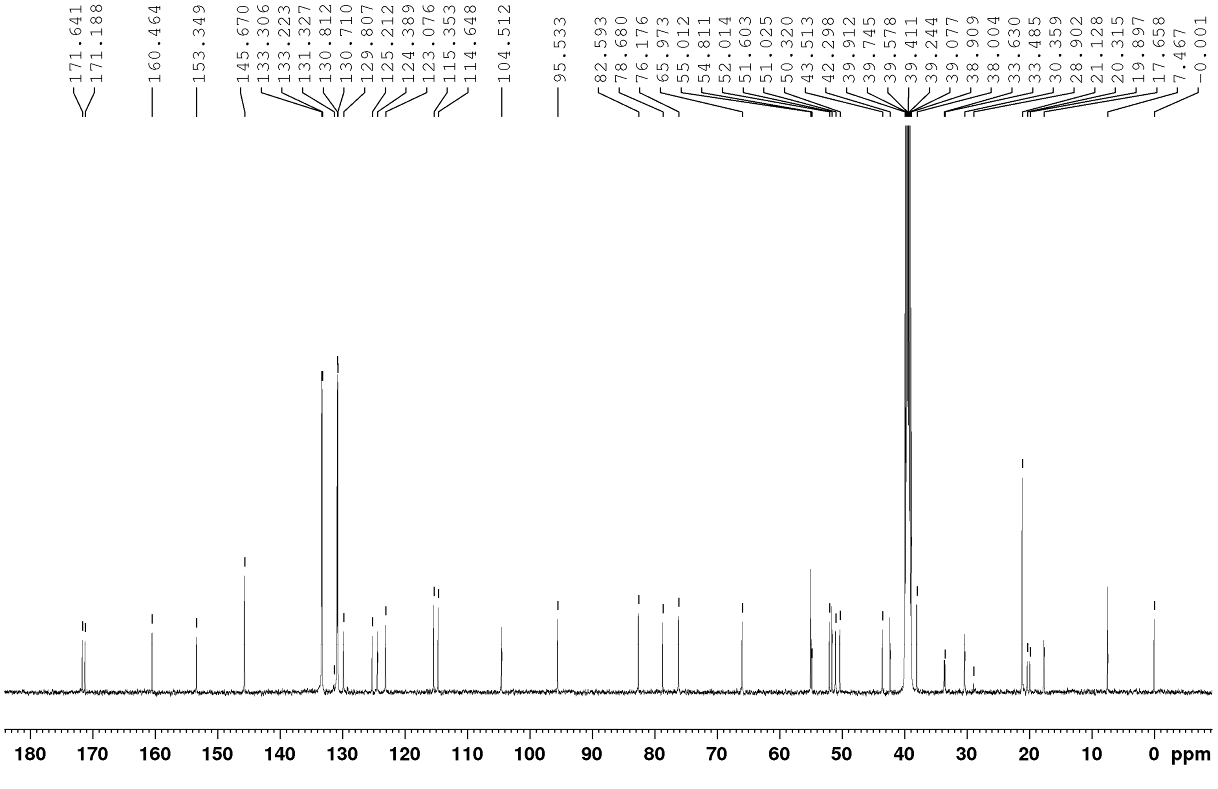

Figure S2. ^{13}C NMR spectrum of compound **9a**.

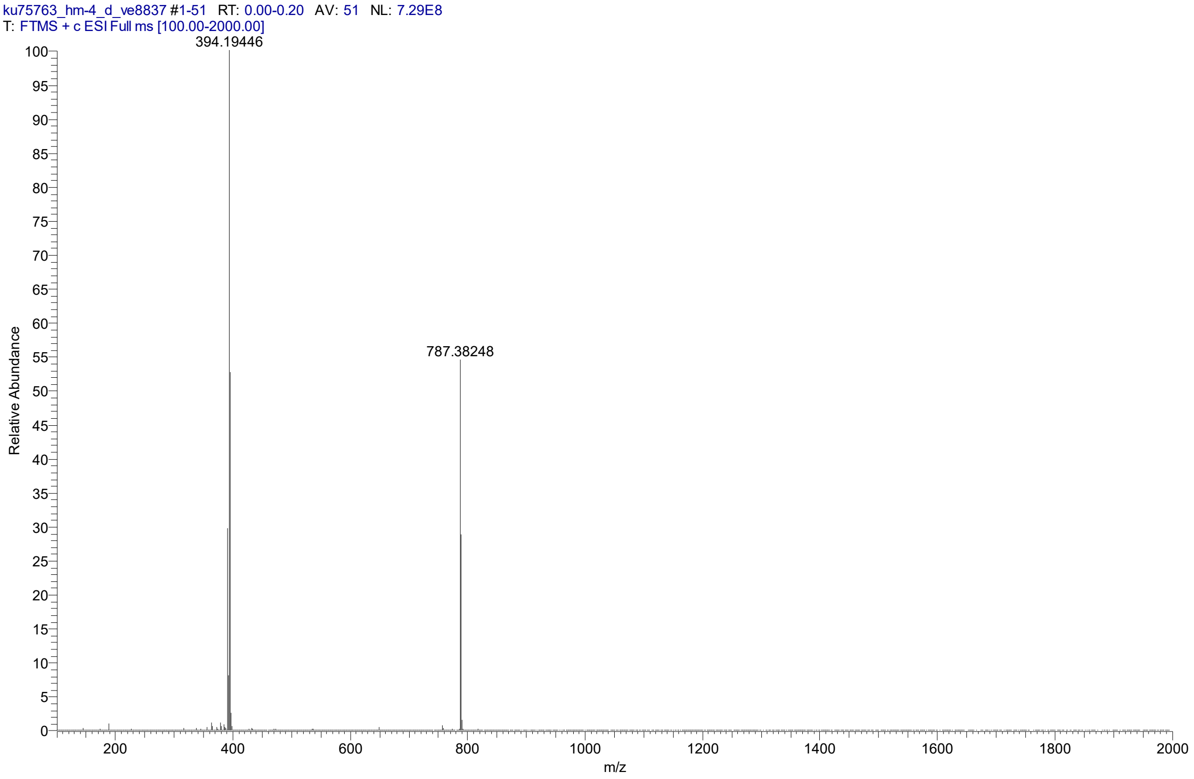

Figure S3. HRMS spectrum of compound **9a**.

S1.2. Product 9b

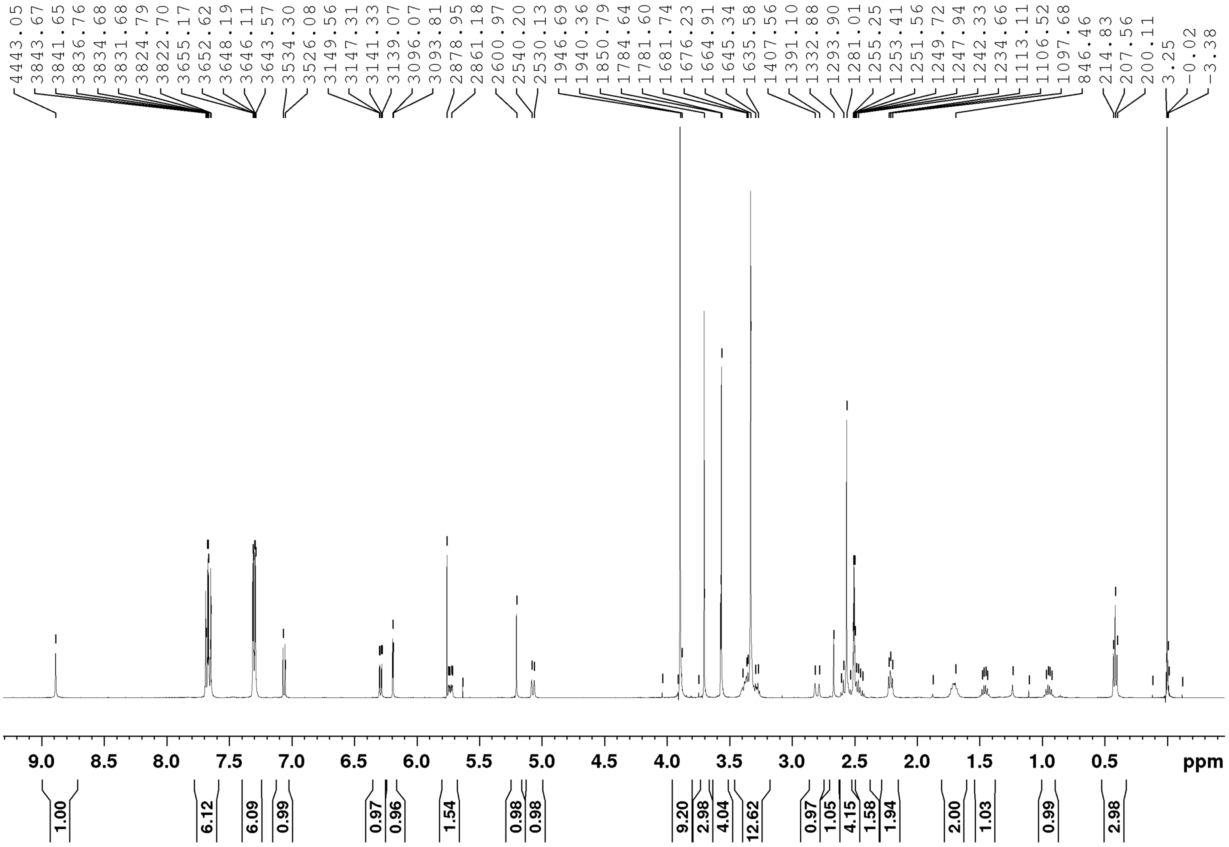

Figure S4. ^{1}H NMR spectrum of compound **9b**.

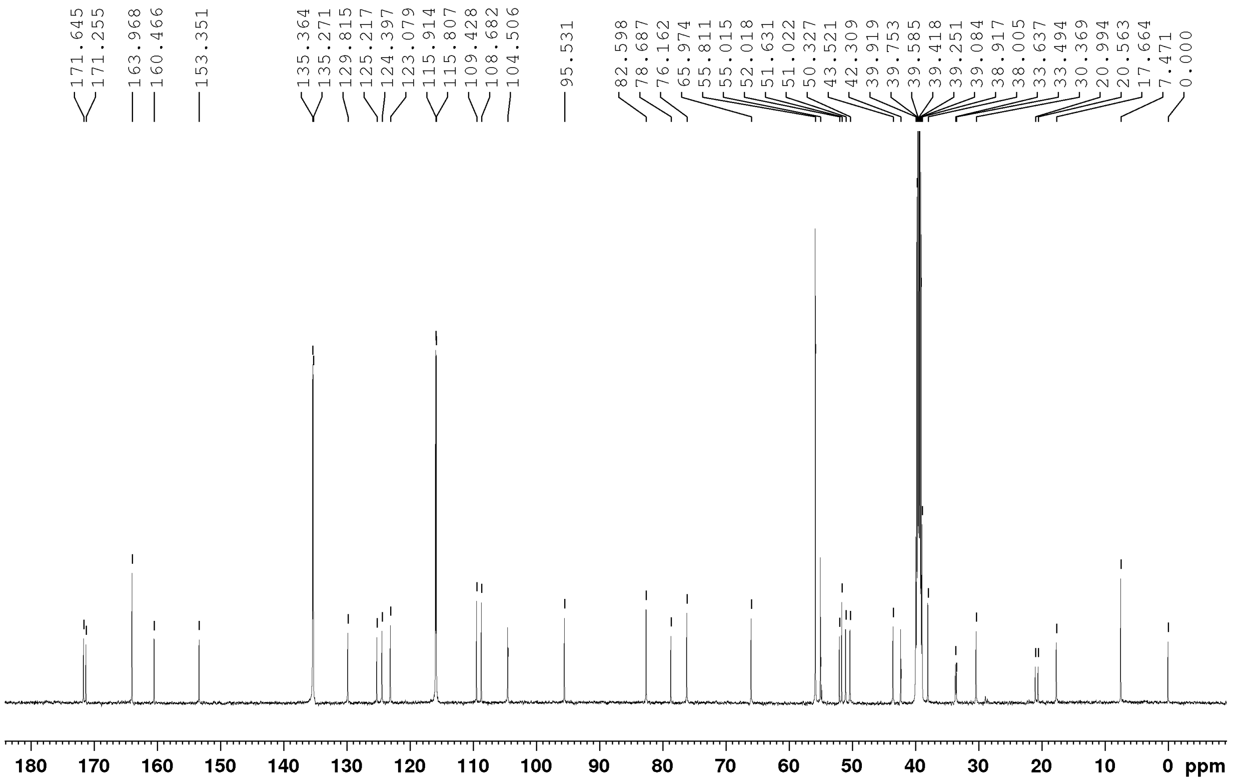

Figure S5. ^{13}C NMR spectrum of compound **9b**.

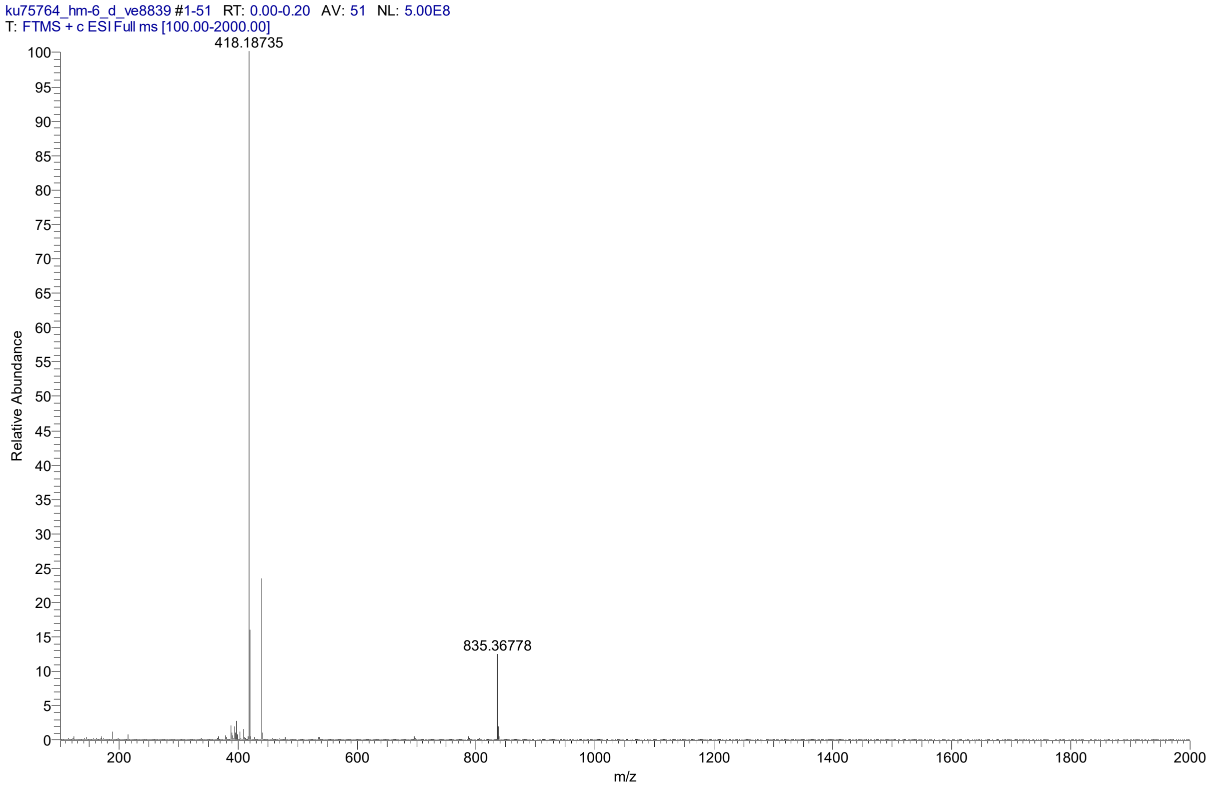

Figure S6. HRMS spectrum of compound **9b**.

S1.3. Product 9c

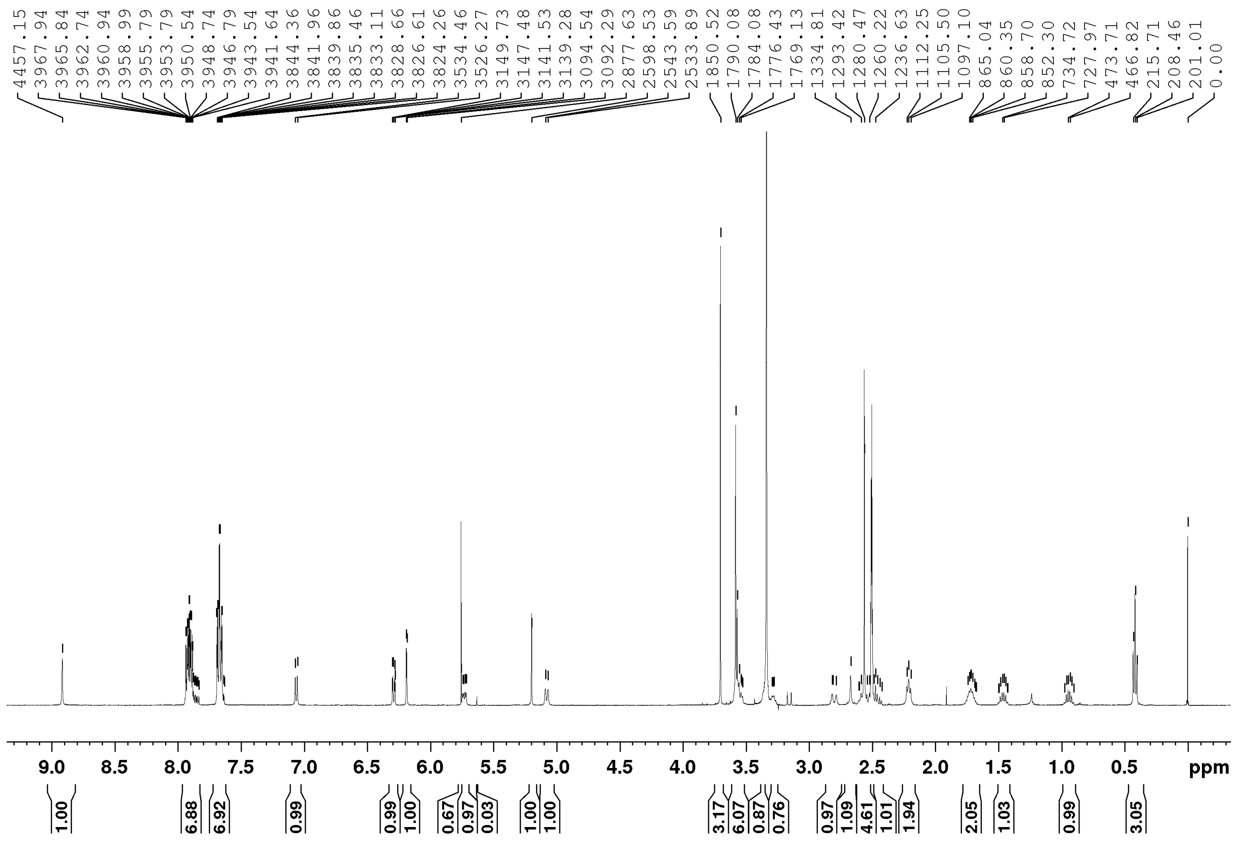

Figure S7. ^{1}H NMR spectrum of compound **9c**.

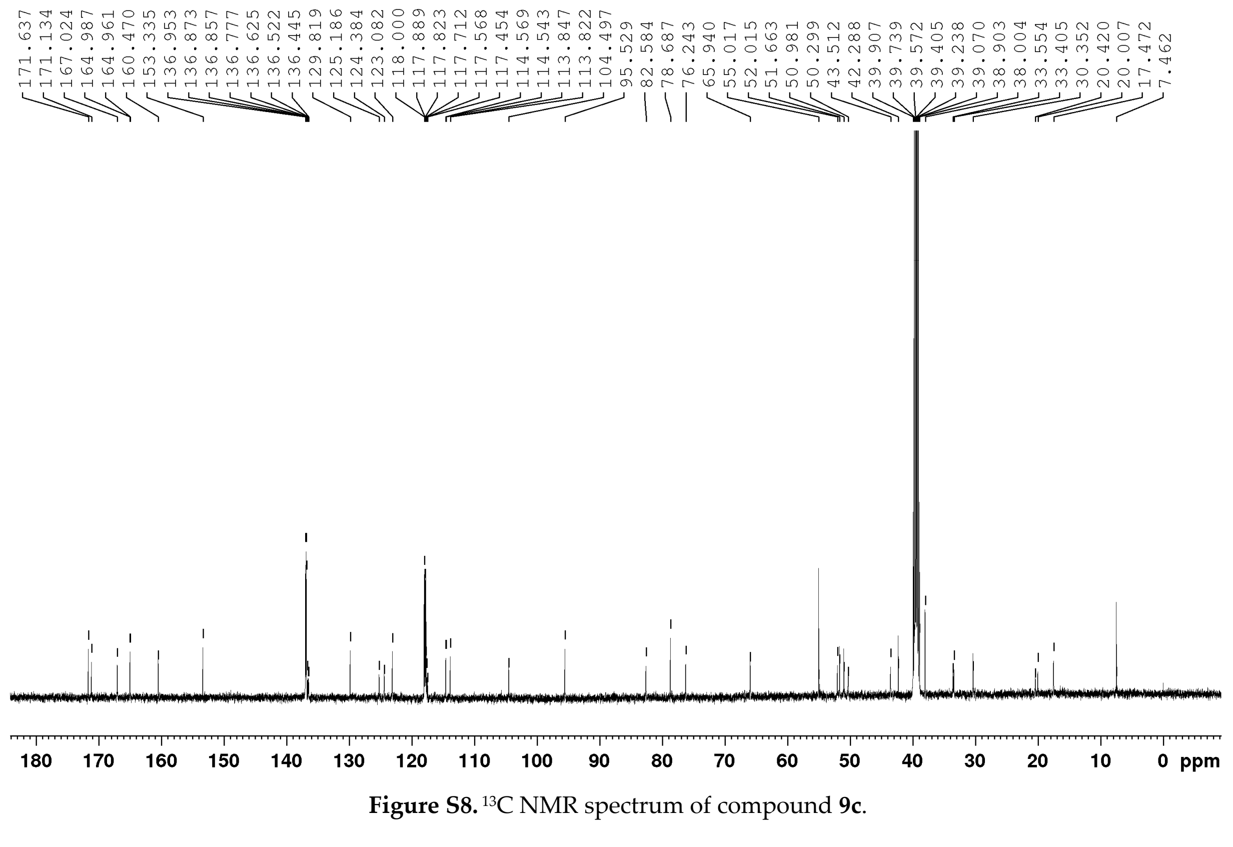

Figure S8. ^{13}C NMR spectrum of compound **9c**.

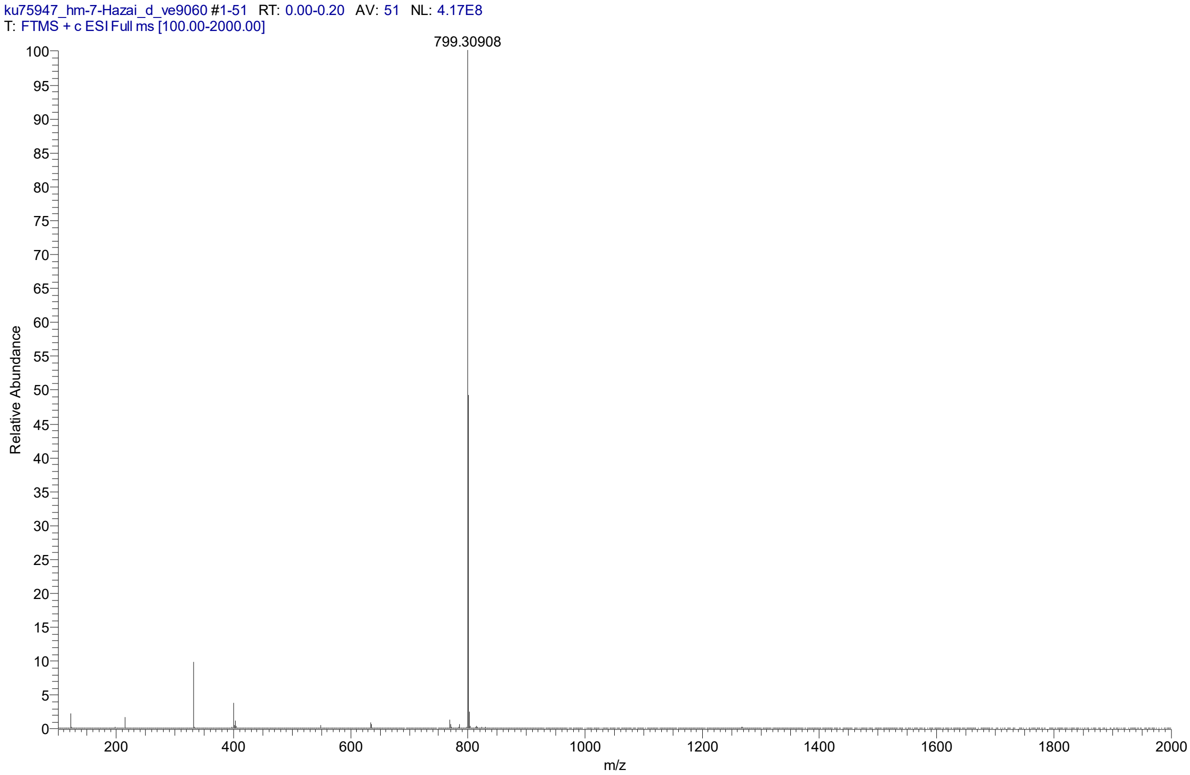

Figure S9. HRMS spectrum of compound **9c**.

S1.4. Product 9d

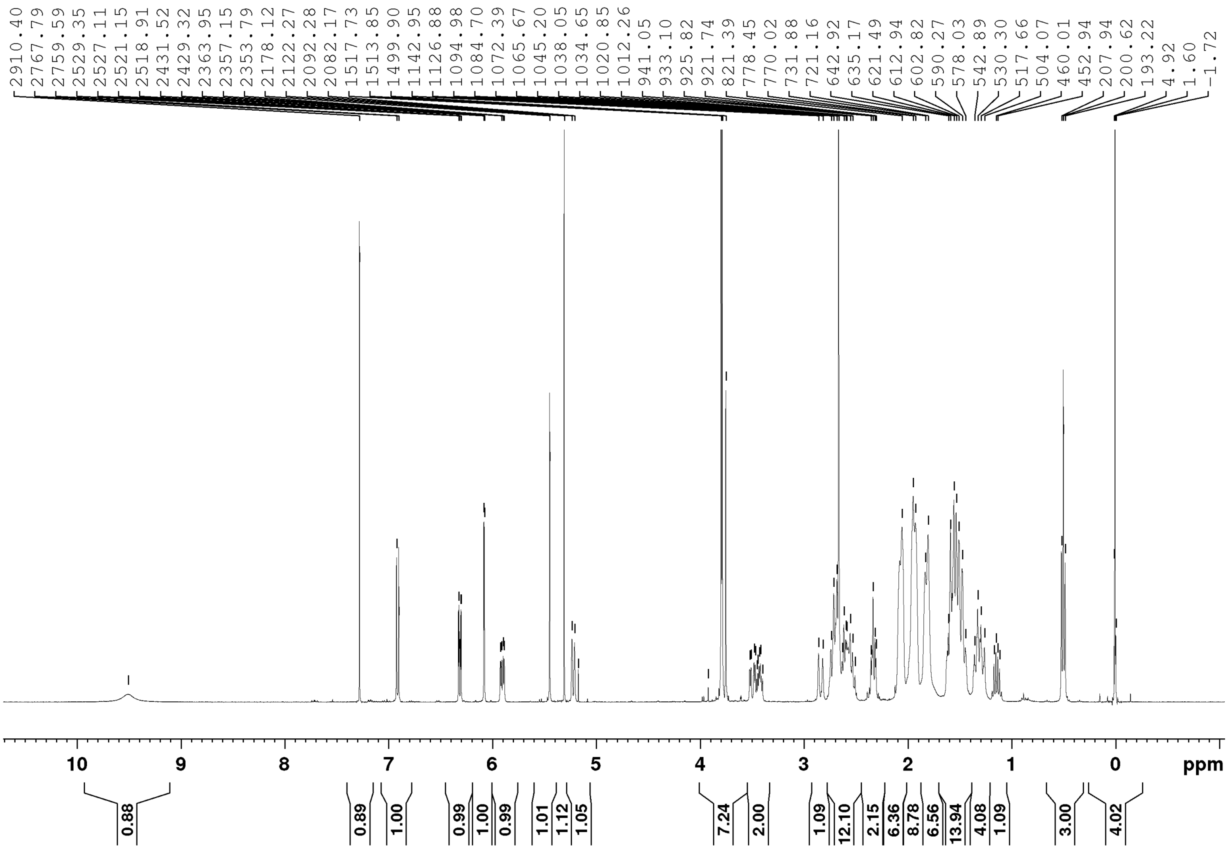

Figure S10. ^{1}H NMR spectrum of compound **9d**.

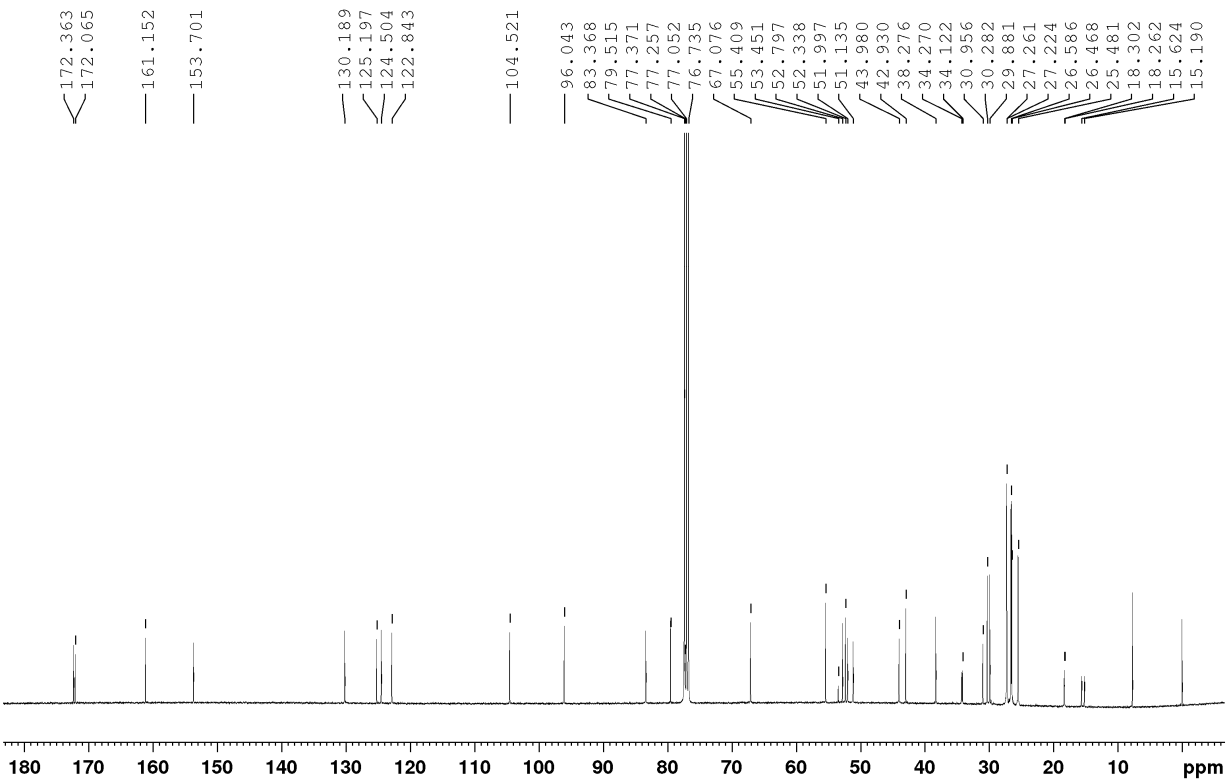

Figure S11. ^{13}C NMR spectrum of compound **9d**.

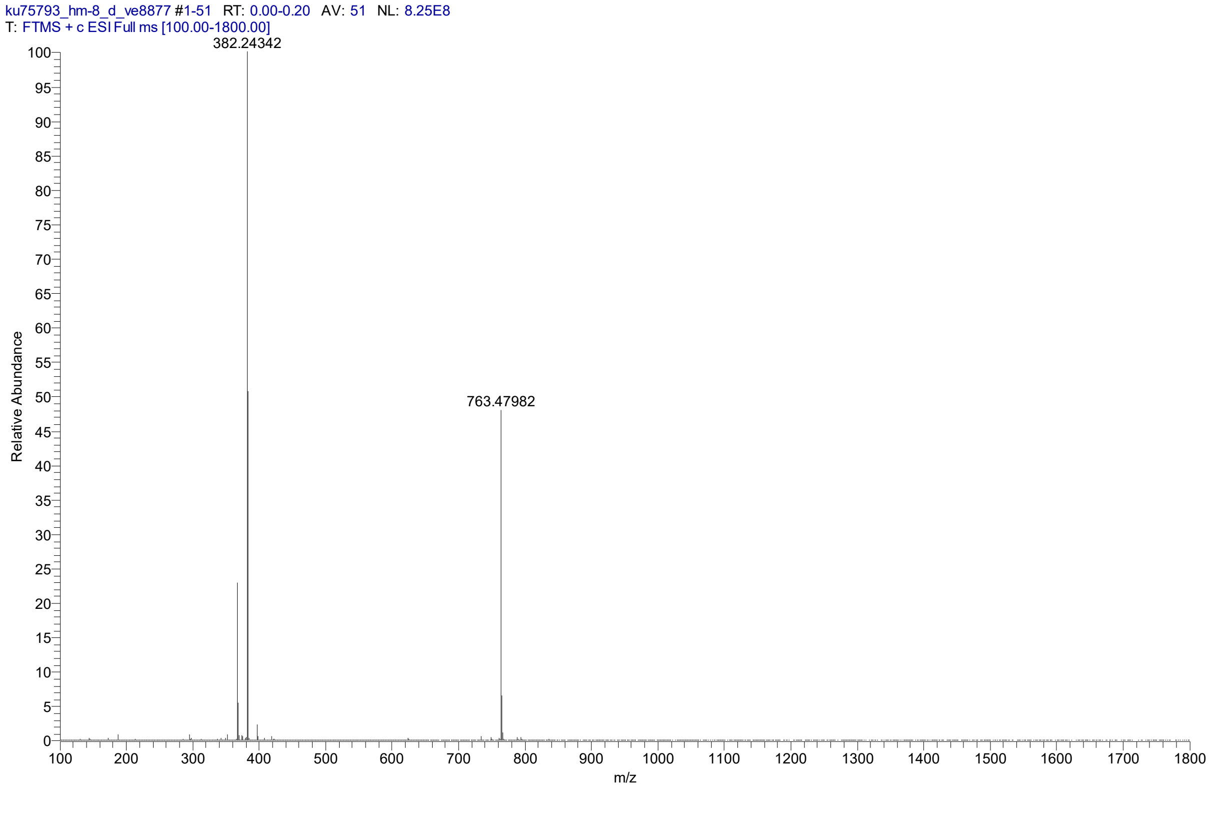

Figure S12. HRMS spectrum of compound **9d**.

S1.5. Product 9e

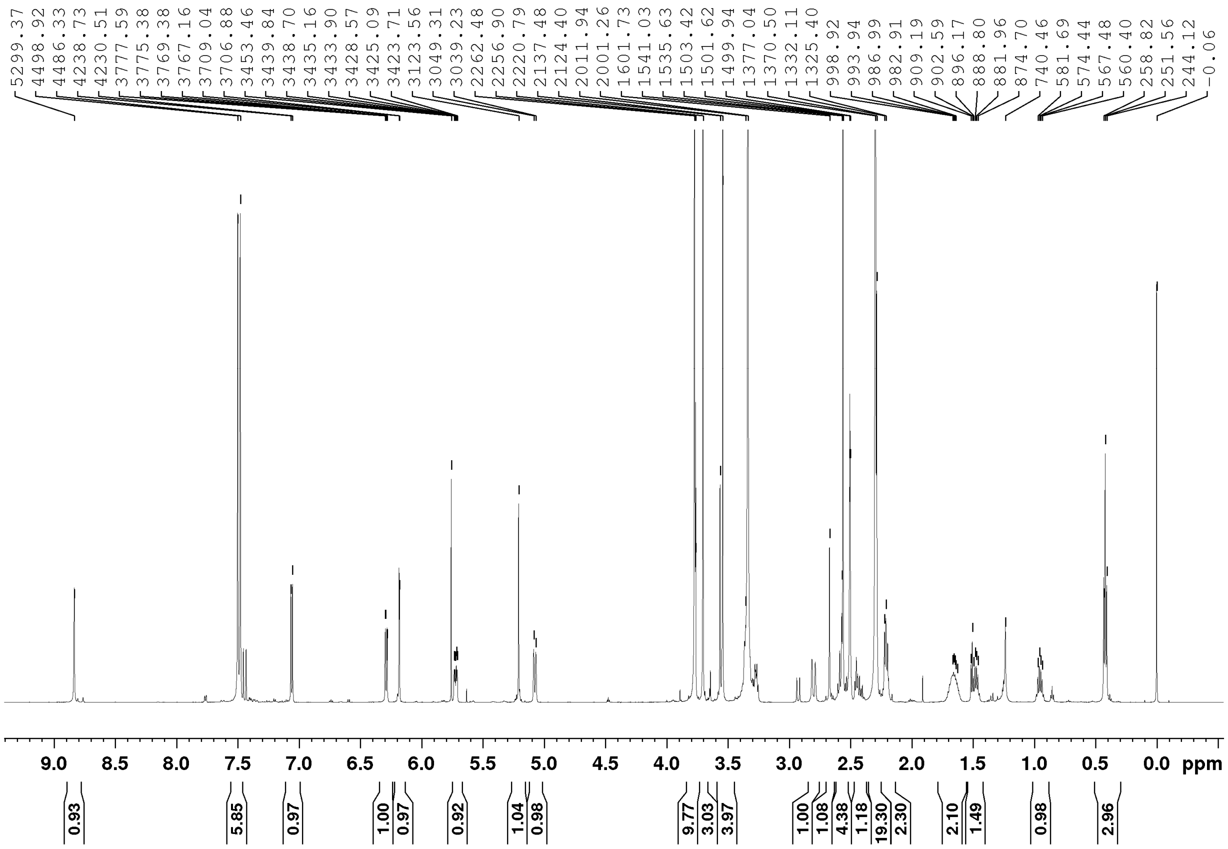

Figure S13. ^{1}H NMR spectrum of compound **9e**.

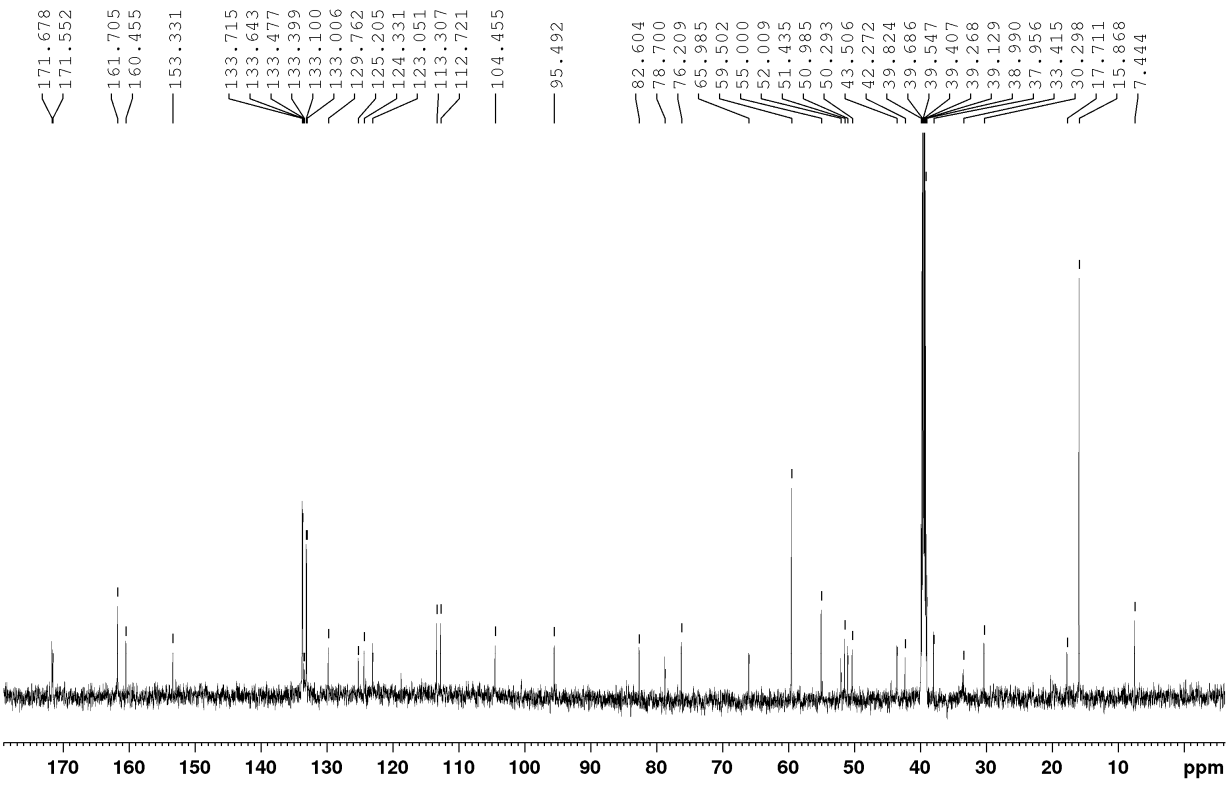

Figure S14. ^{13}C NMR spectrum of compound **9e**.

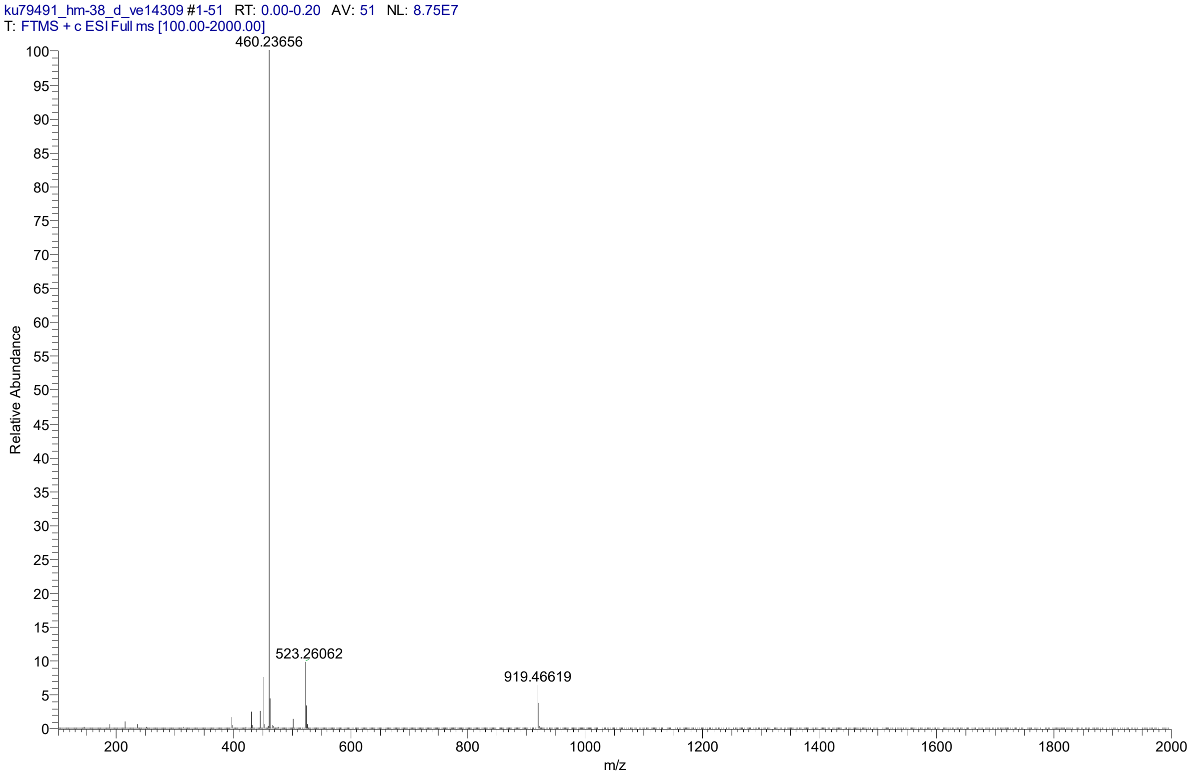

Figure S15. HRMS spectrum of compound **9e**.

S1.6. Product 9f

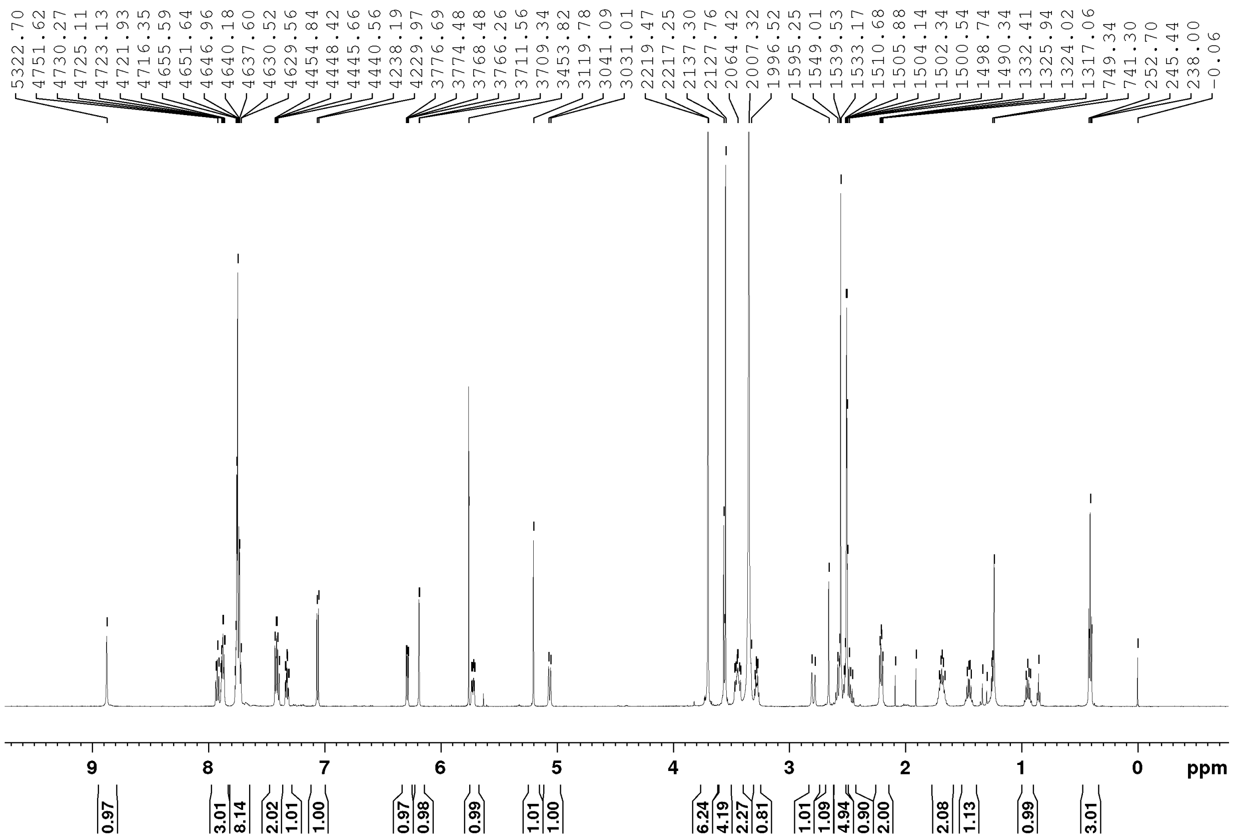

Figure S16. ^{1}H NMR spectrum of compound **9f**.

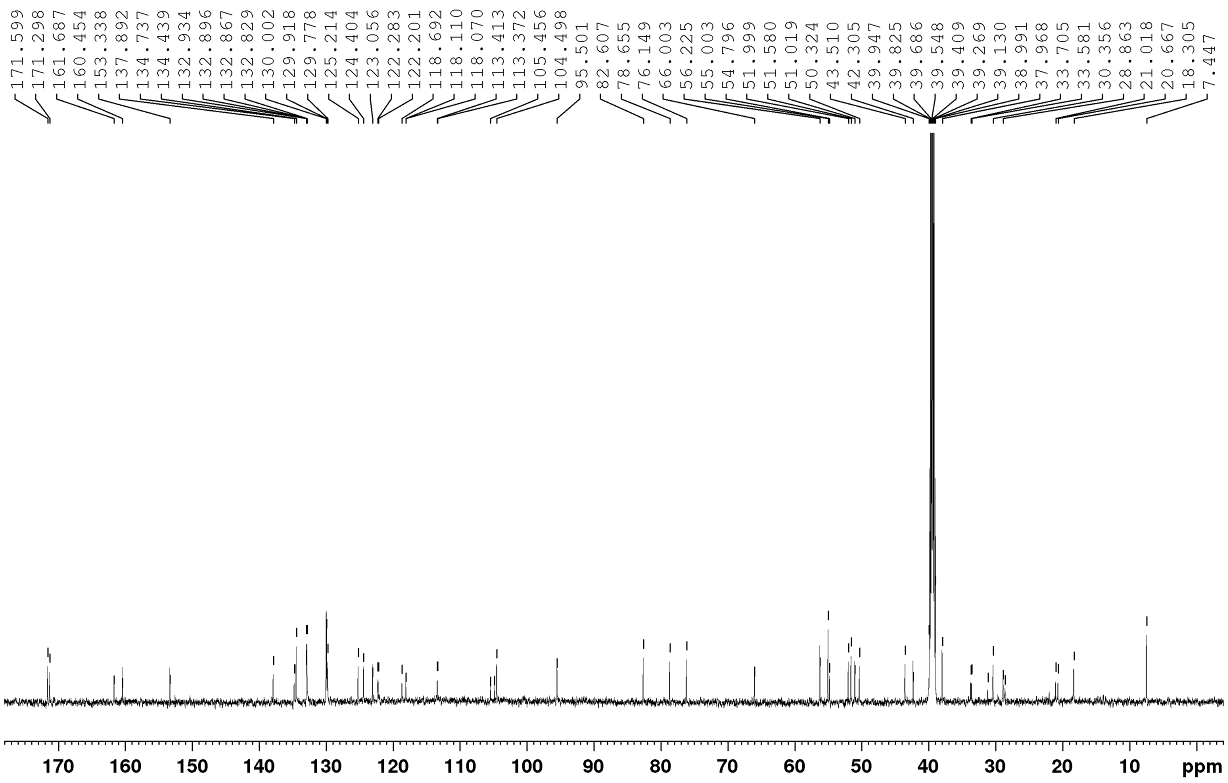

Figure S17. ^{13}C NMR spectrum of compound **9f**.

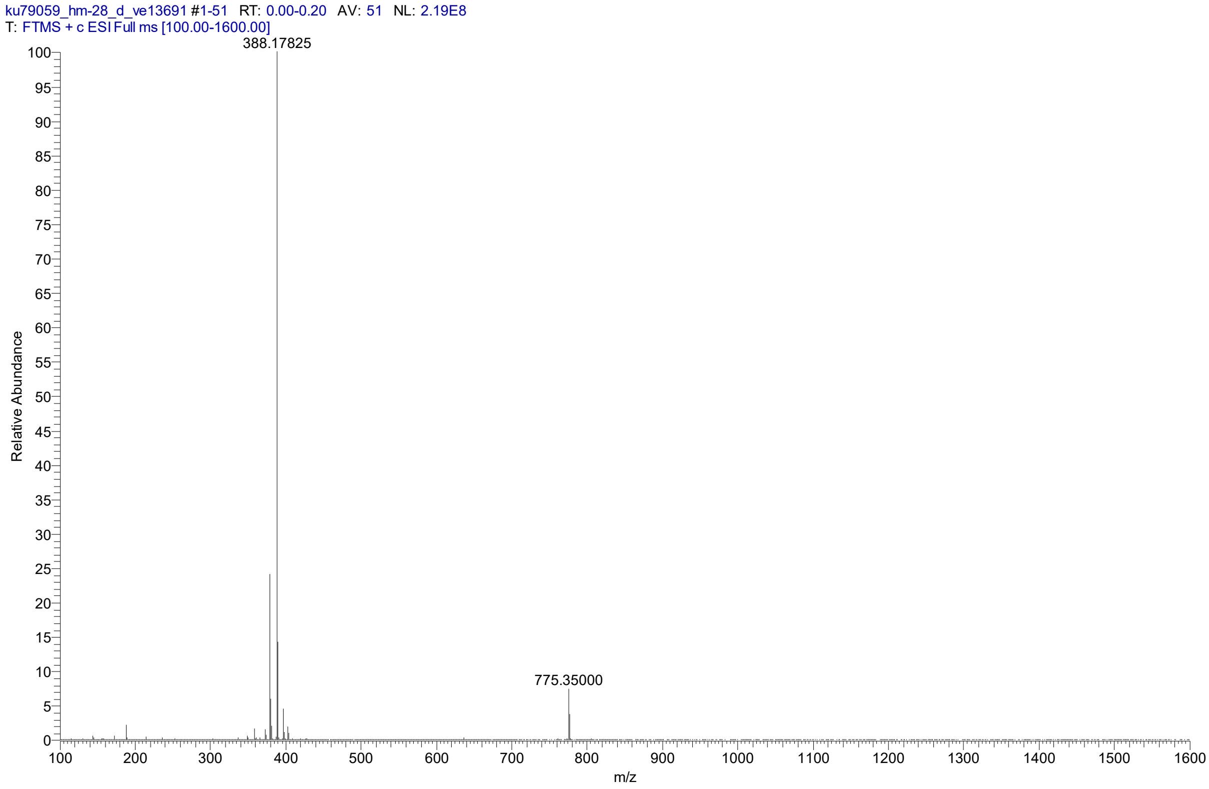

Figure S18. HRMS spectrum of compound **9f**.

S1.7. Product 9g

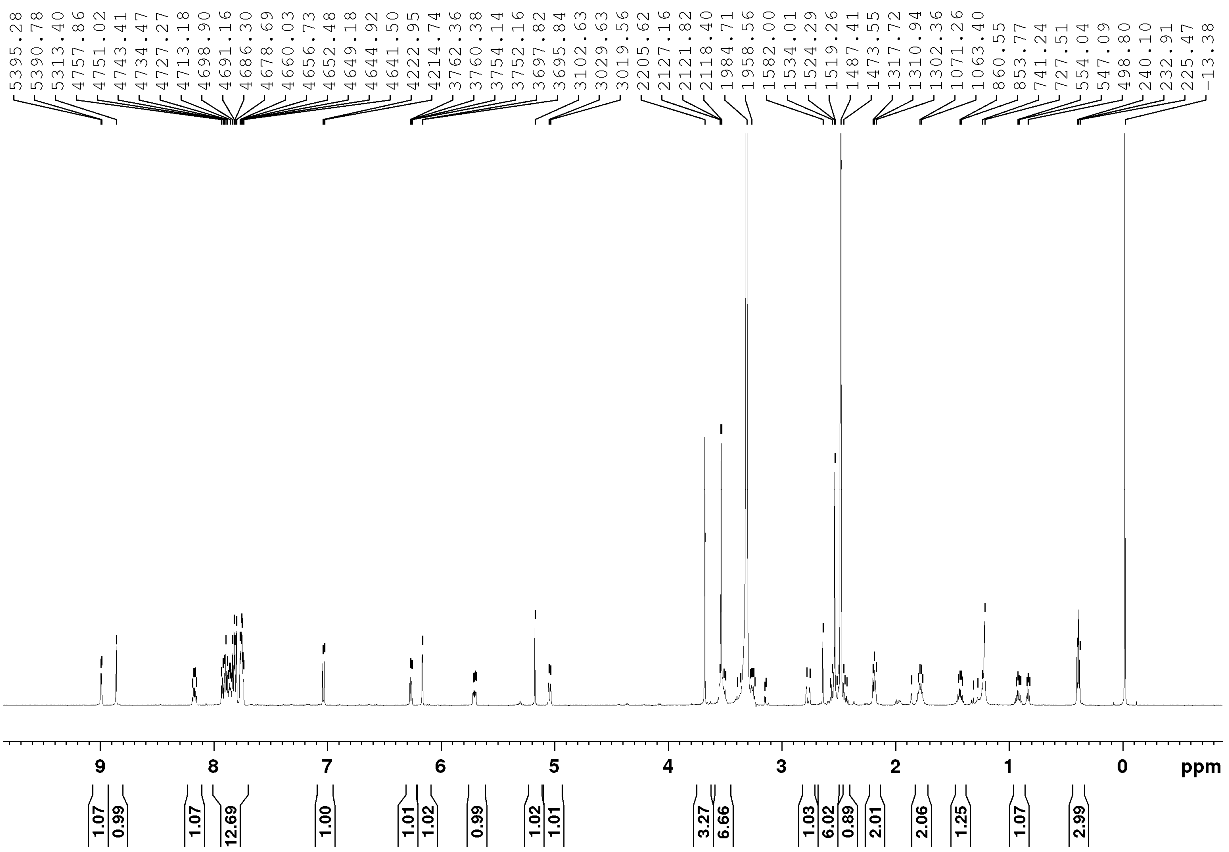

Figure S19. ^{1}H NMR spectrum of compound **9g**.

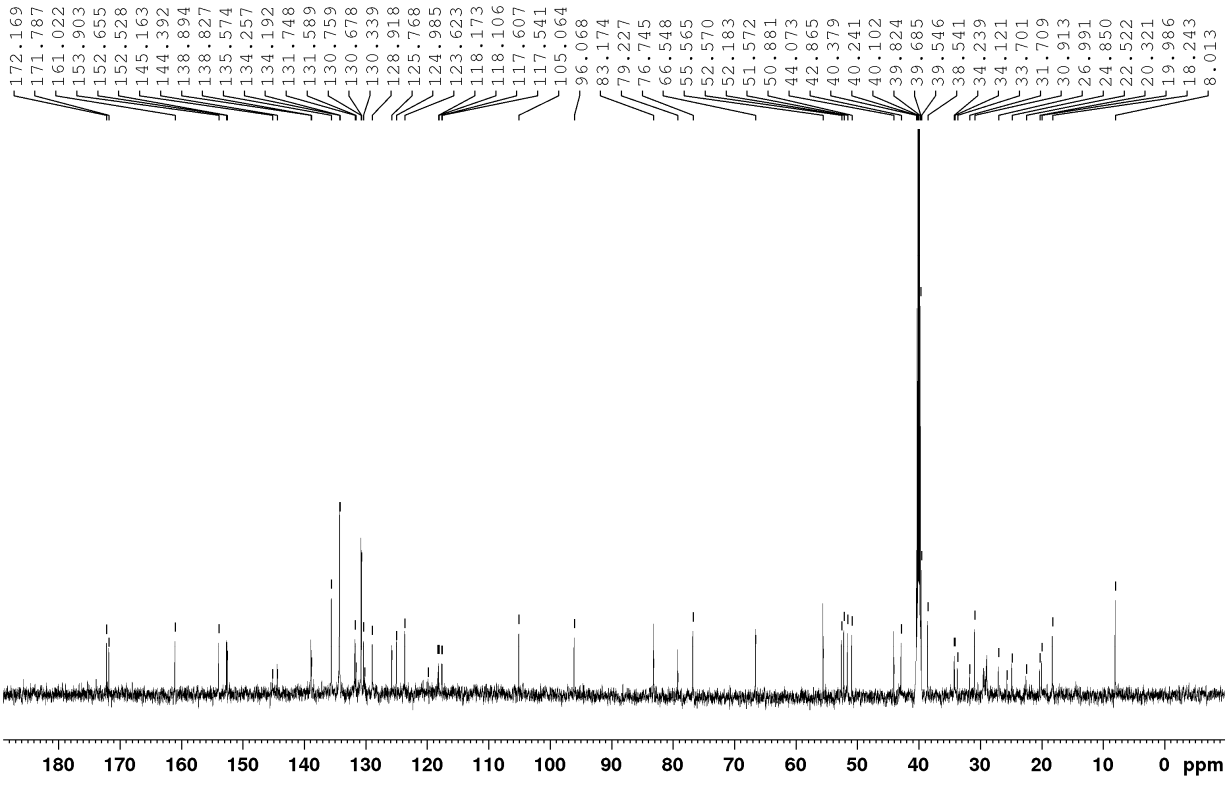

Figure S20. ^{13}C NMR spectrum of compound **9g**.

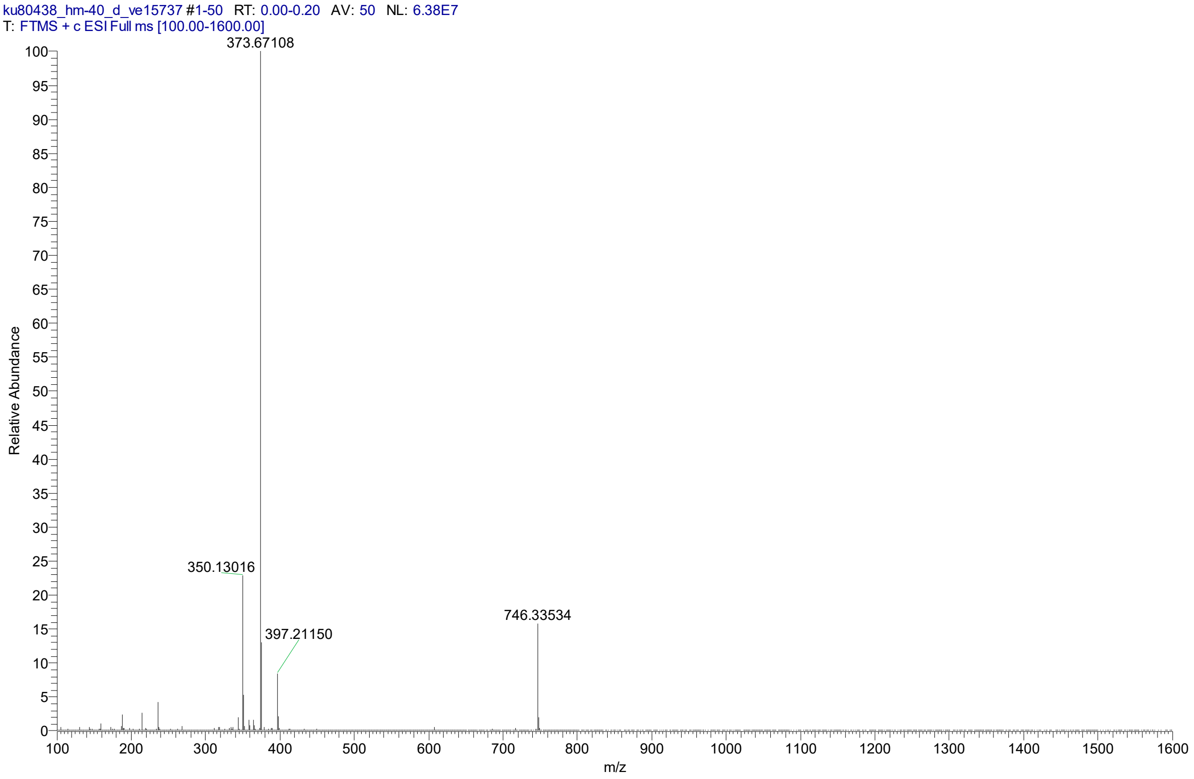

Figure S21. HRMS spectrum of compound **9g**.

S1.8. Product 10a

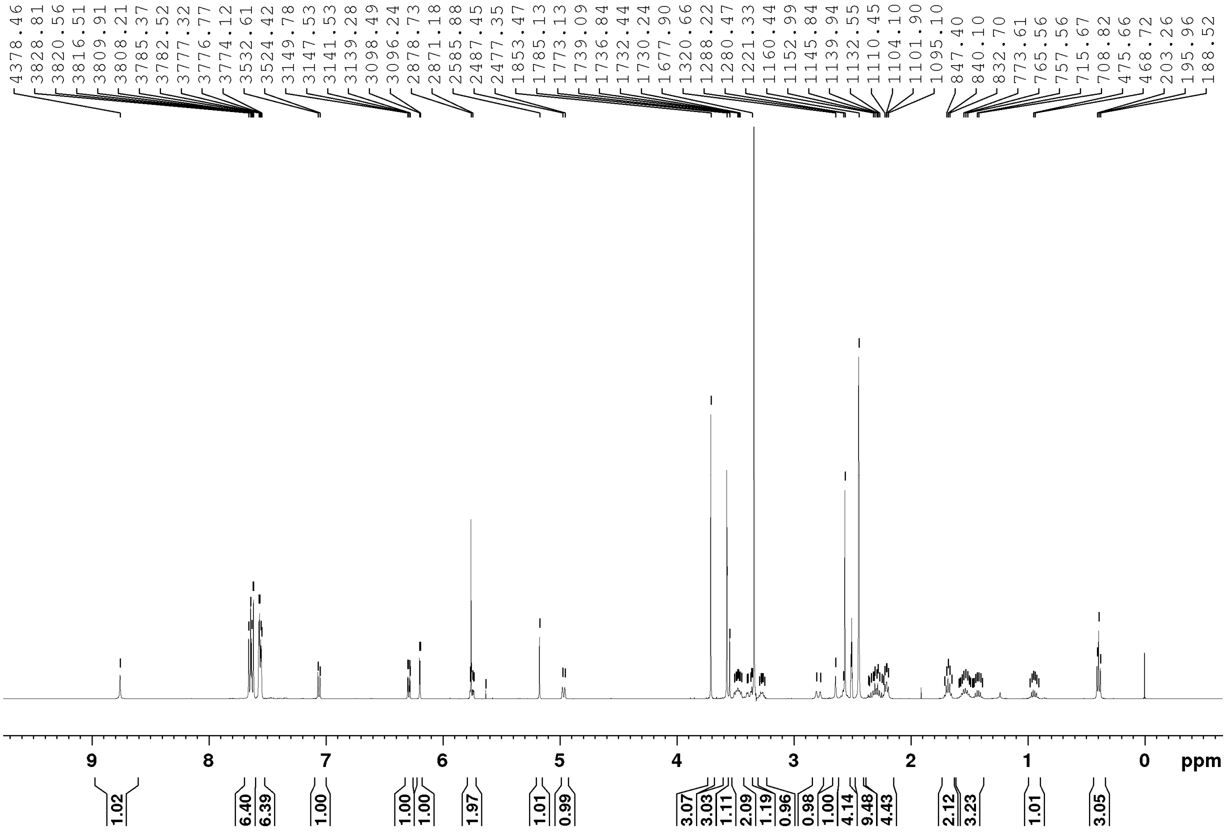

Figure S22. ^{1}H NMR spectrum of compound **10a**.

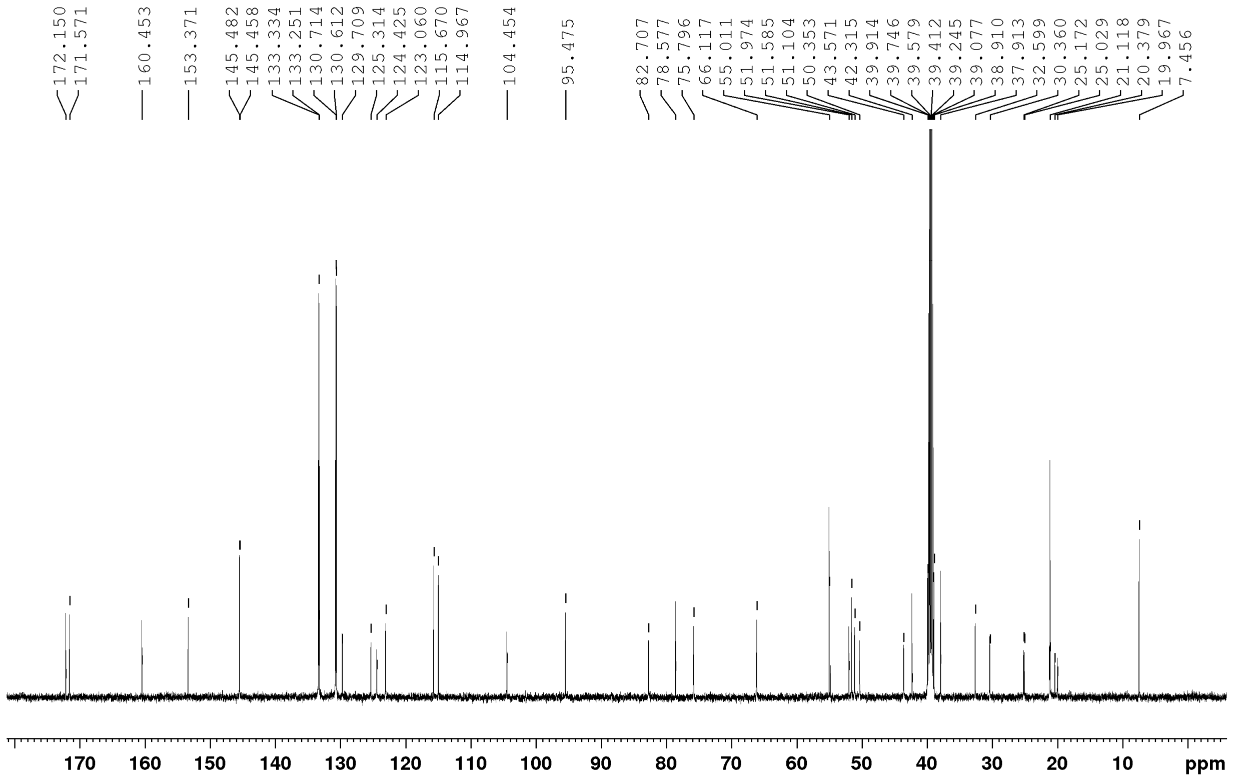

Figure S23. ^{13}C NMR spectrum of compound **10a**.

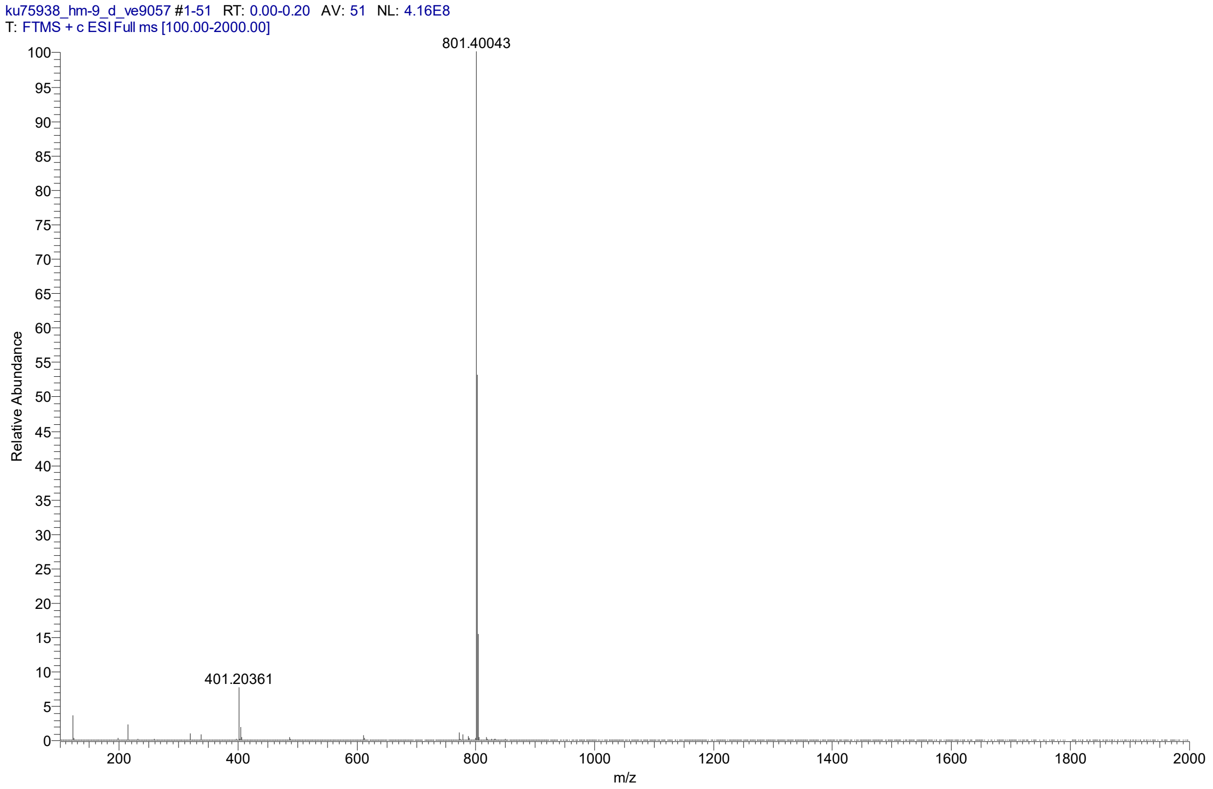

Figure S24. HRMS spectrum of compound **10a**.

S1.9. Product 10b

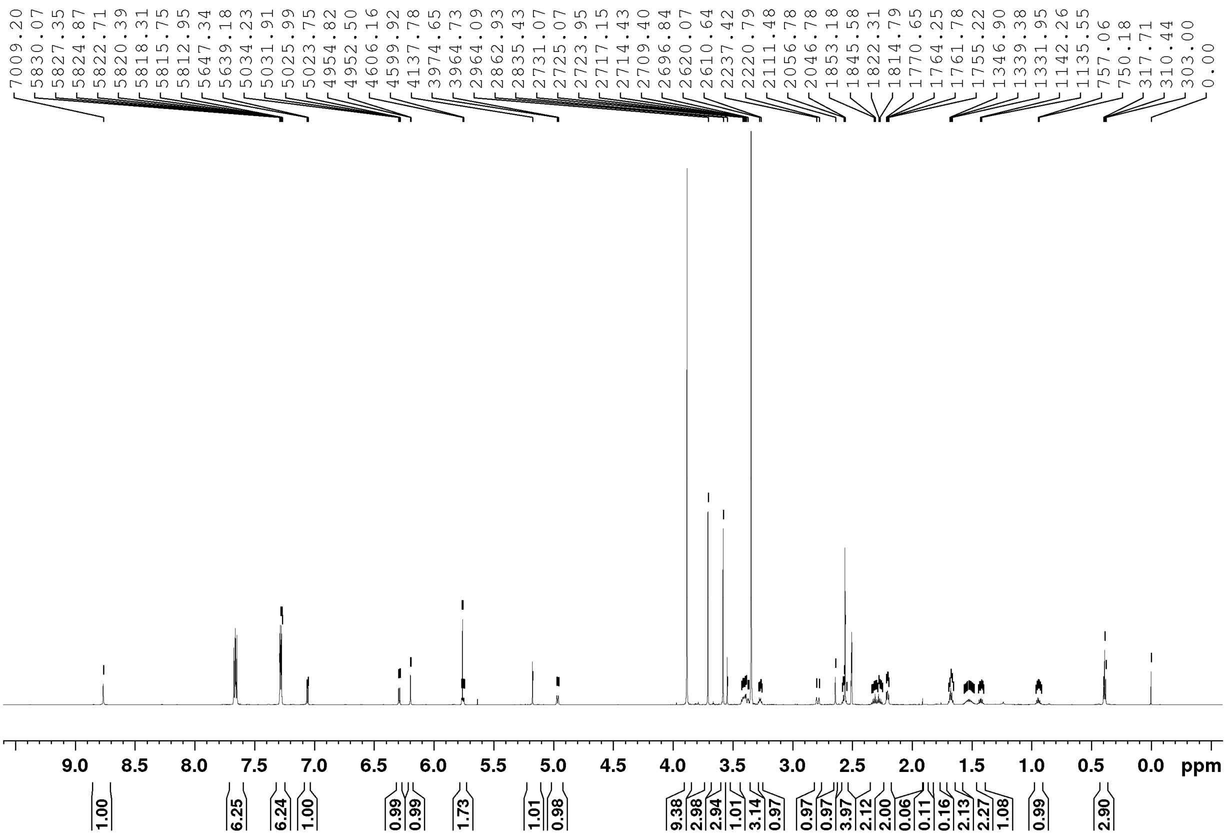

Figure S25. ^{1}H NMR spectrum of compound **10b**.

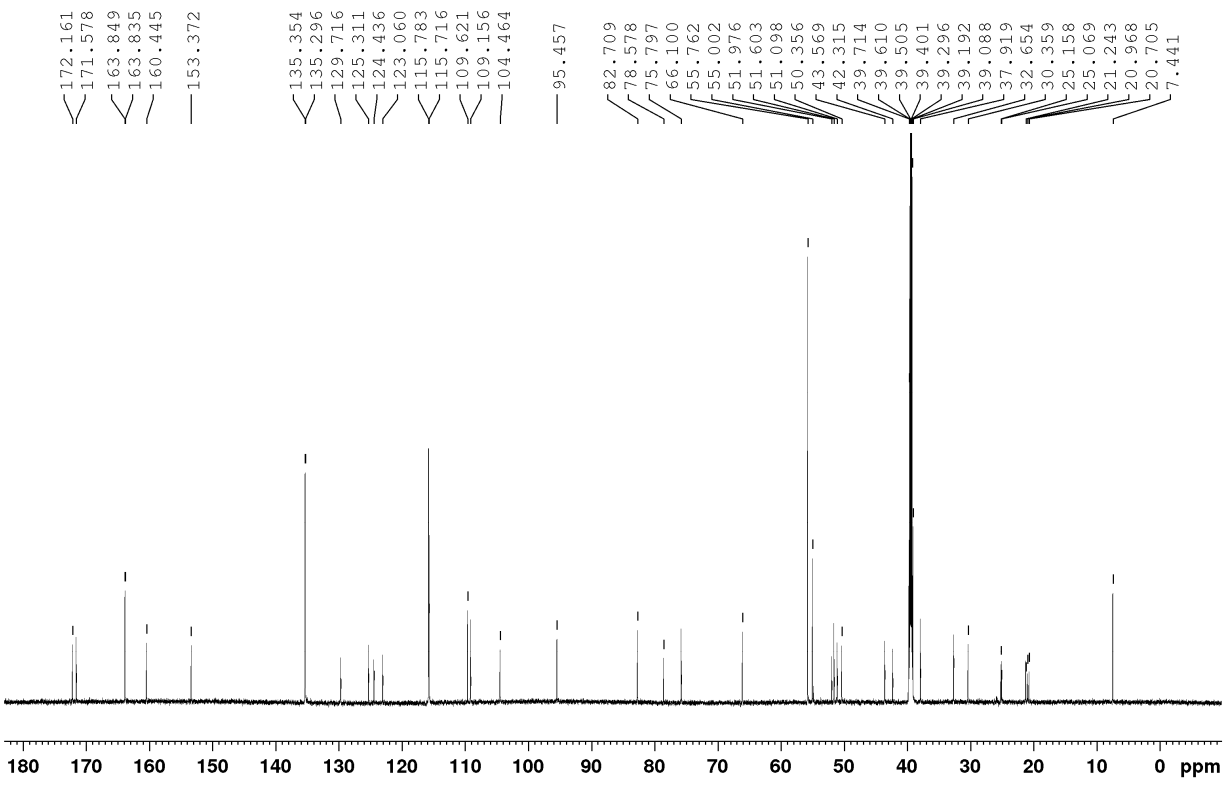

Figure S26. ^{13}C NMR spectrum of compound **10b**.

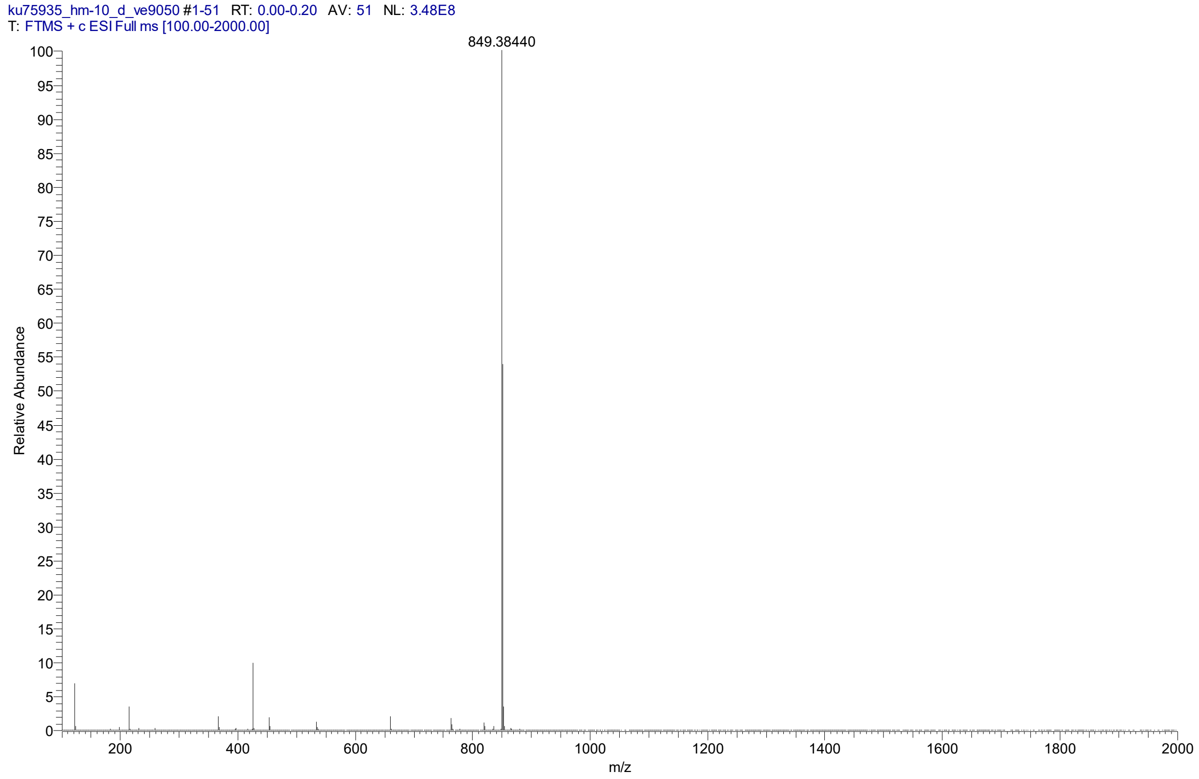

Figure S27. HRMS spectrum of compound **10b**.

S1.10. Product 10c

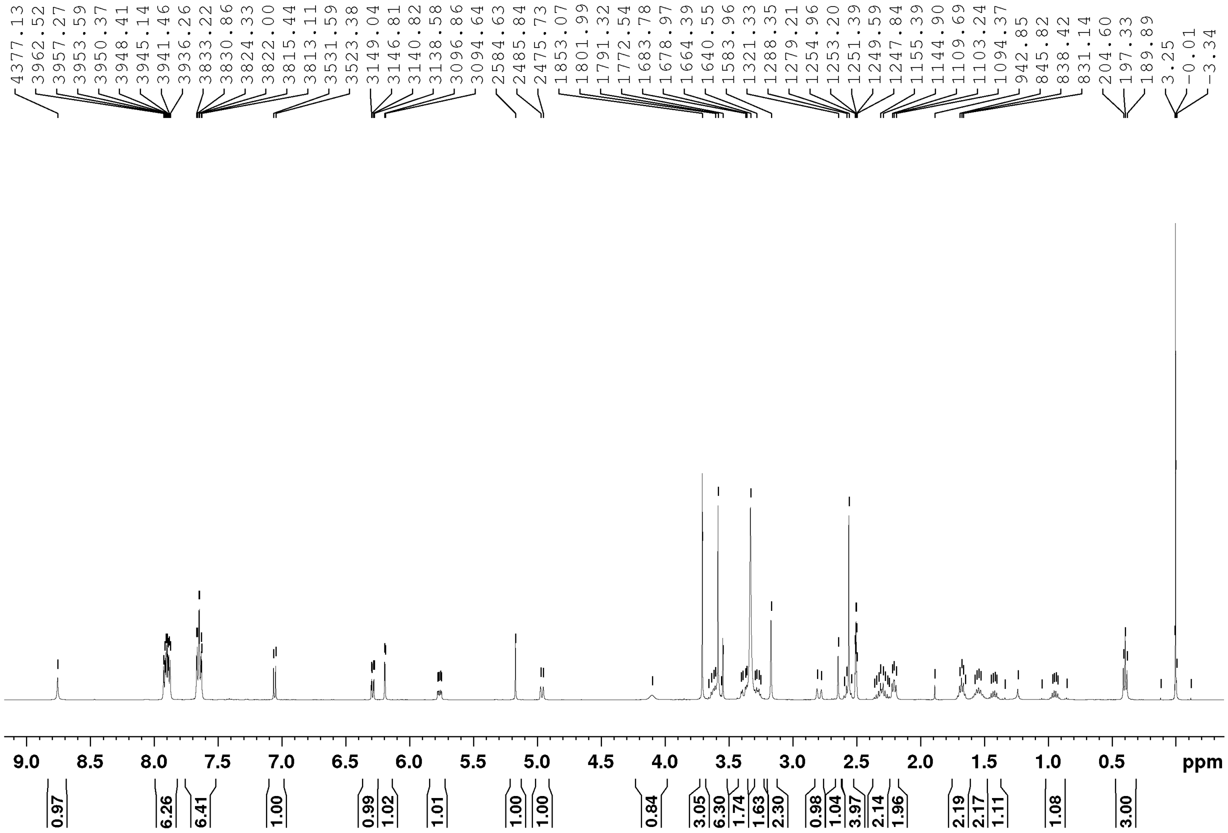

Figure S28. ^{1}H NMR spectrum of compound **10c**.

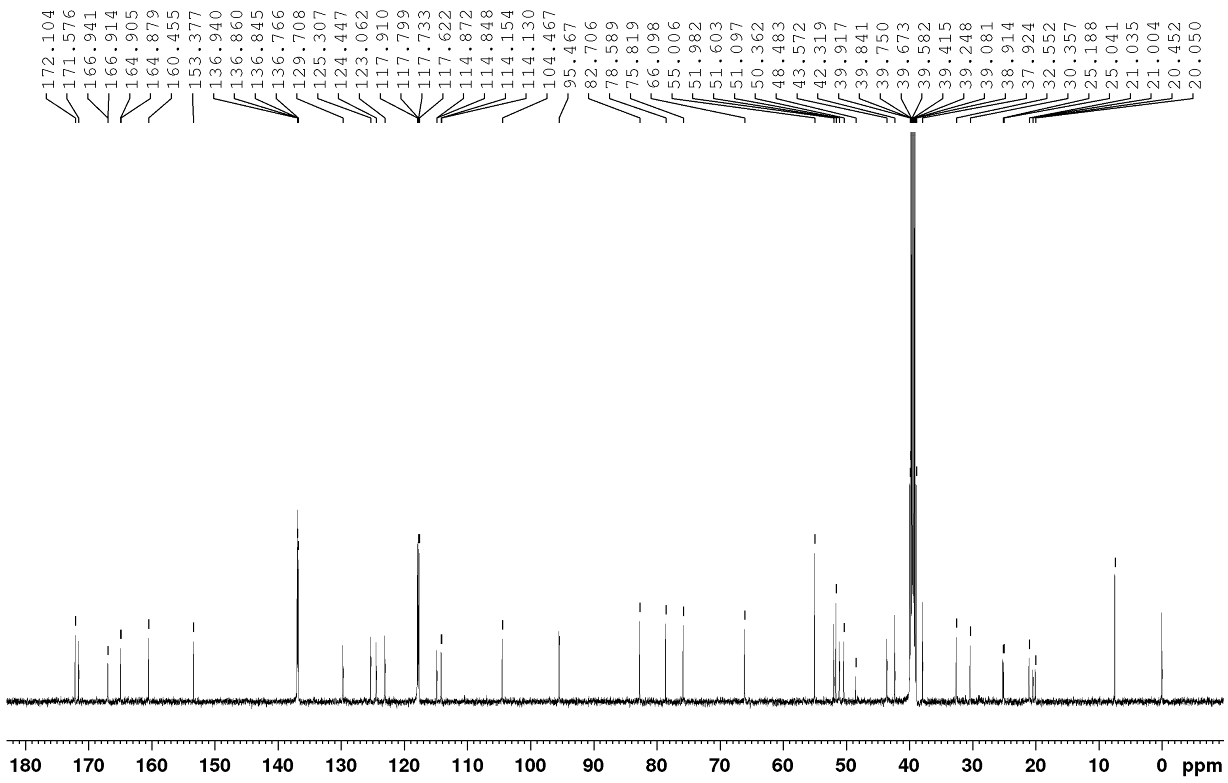

Figure S29. ^{13}C NMR spectrum of compound **10c**.

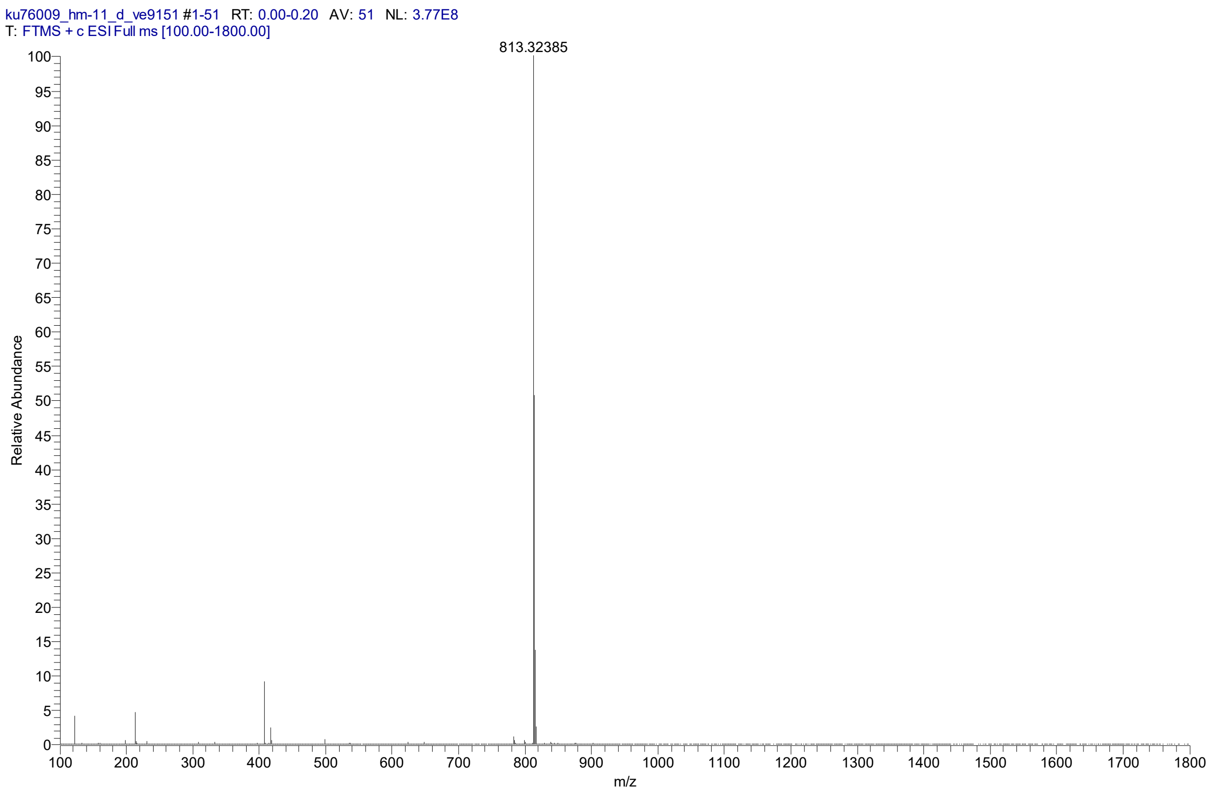

Figure S30. HRMS spectrum of compound **10c**.

S1.11. Product 10d

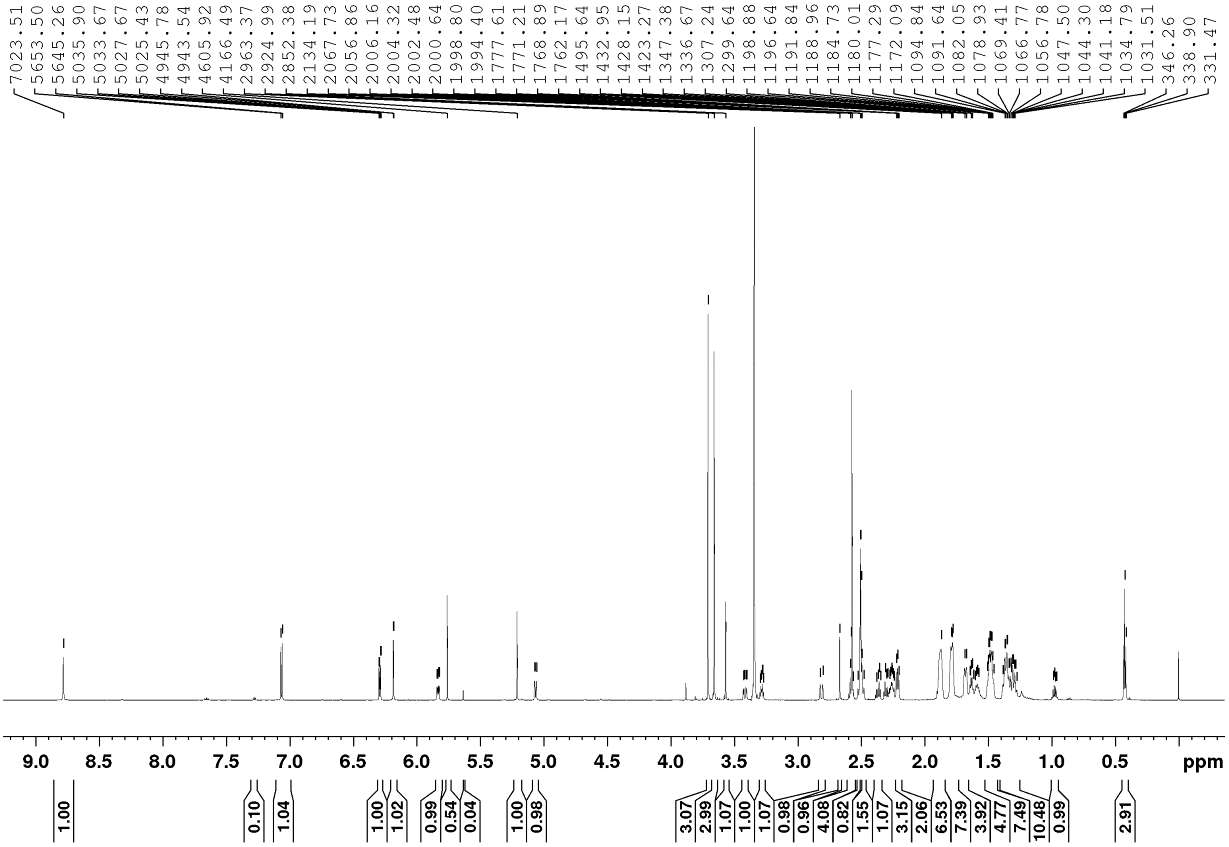

Figure S31. ^{1}H NMR spectrum of compound **10d**.

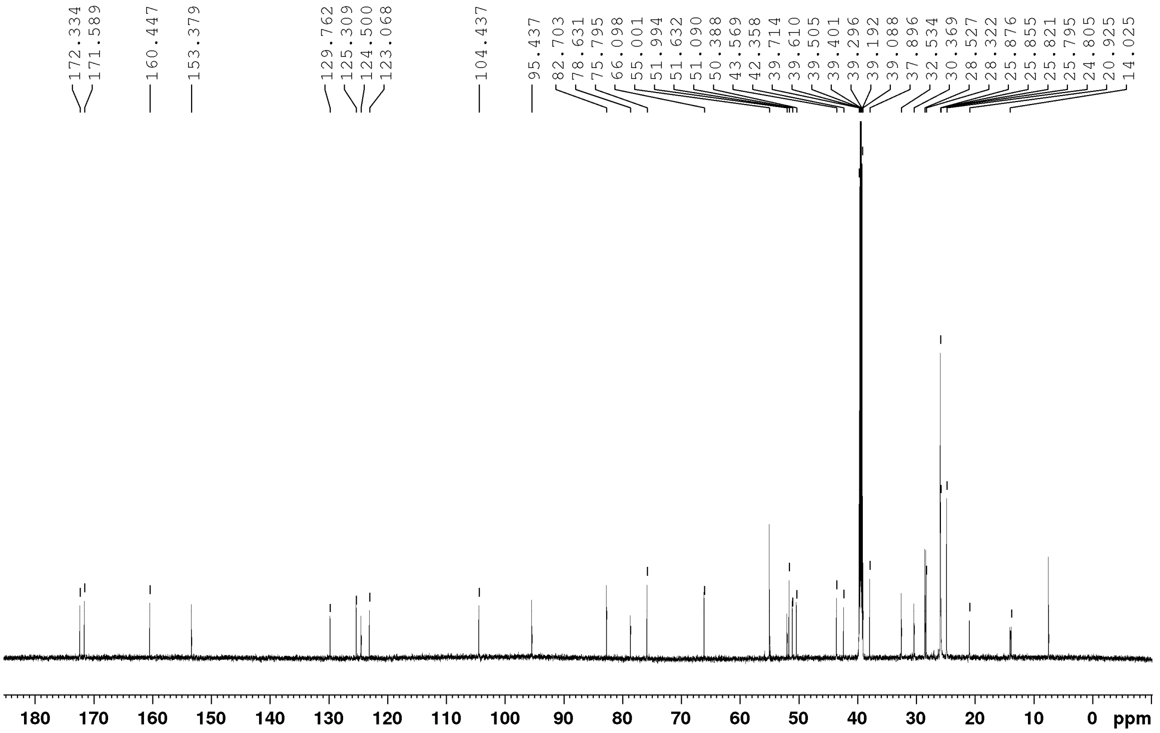

Figure S32. ^{13}C NMR spectrum of compound **10d**.

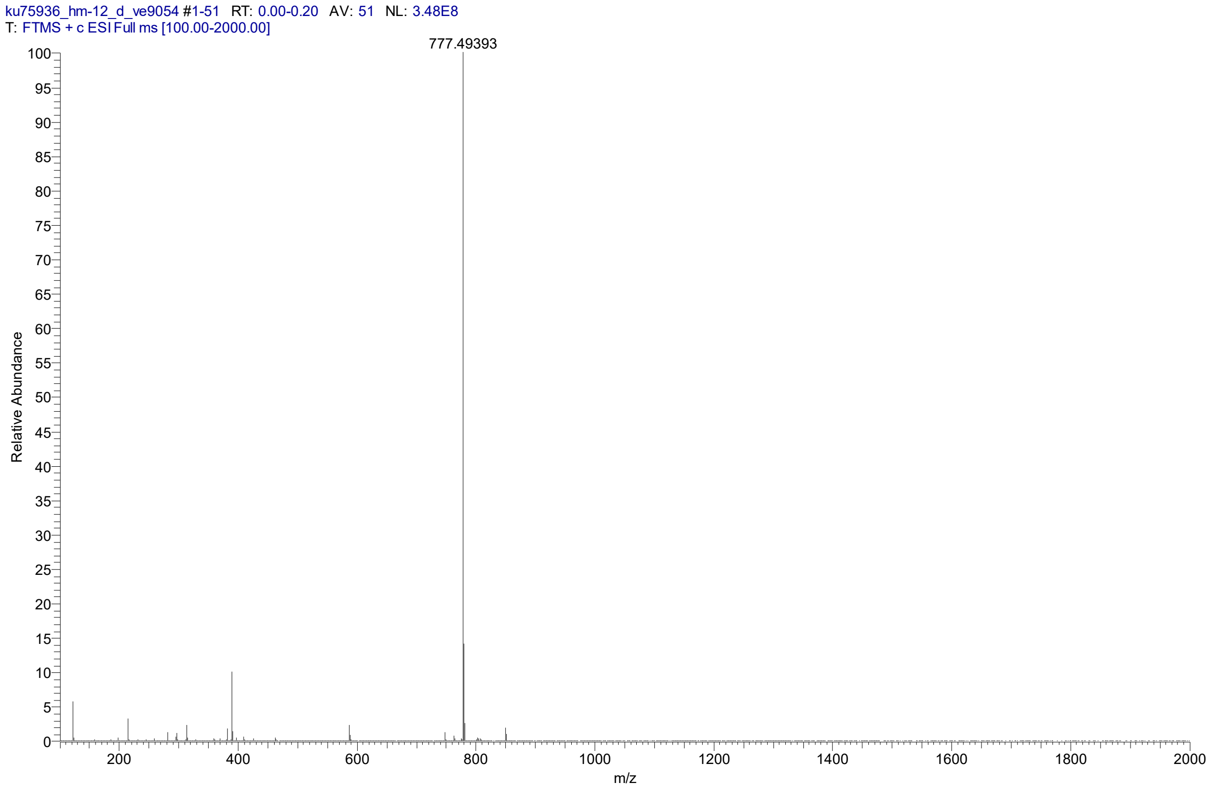

Figure S33. HRMS spectrum of compound **10d**.

S1.12. Product 11

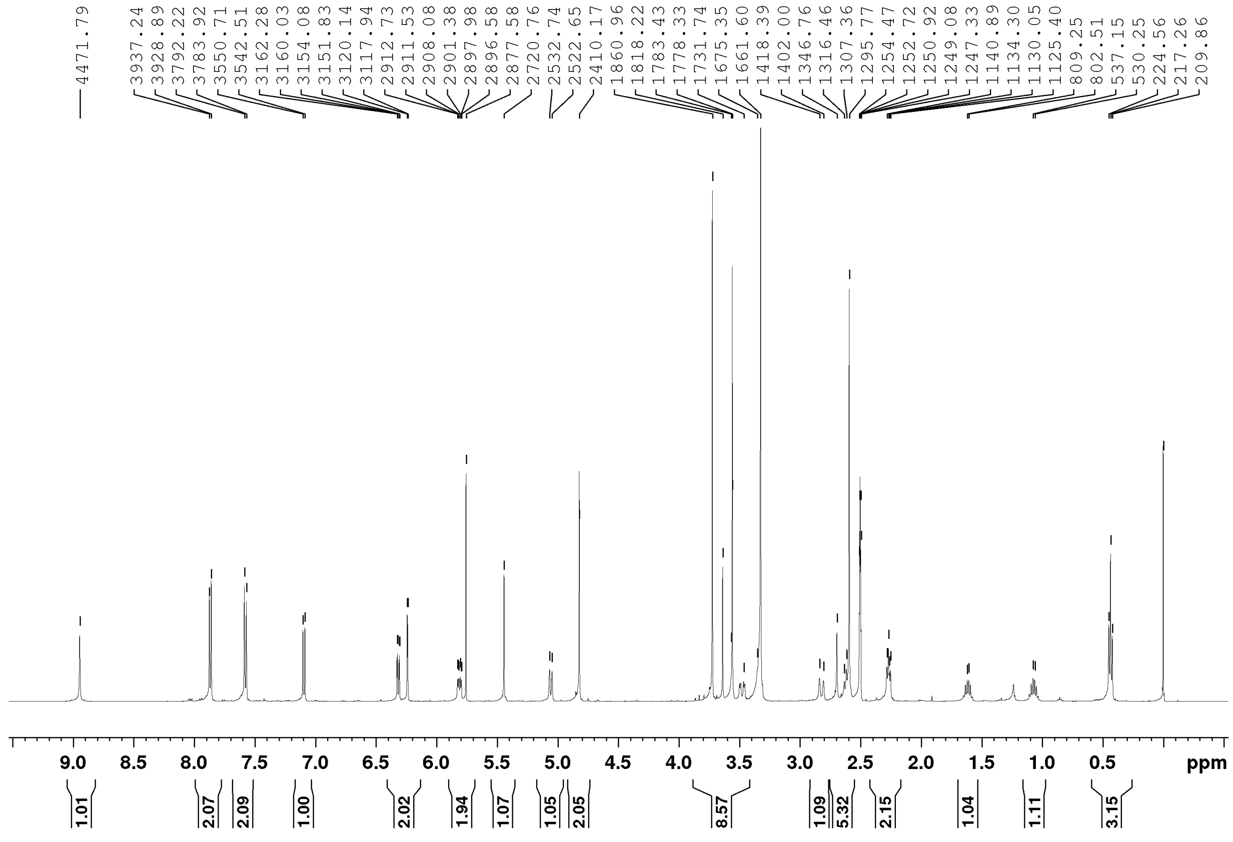

Figure S34. ^{1}H NMR spectrum of compound **11**.

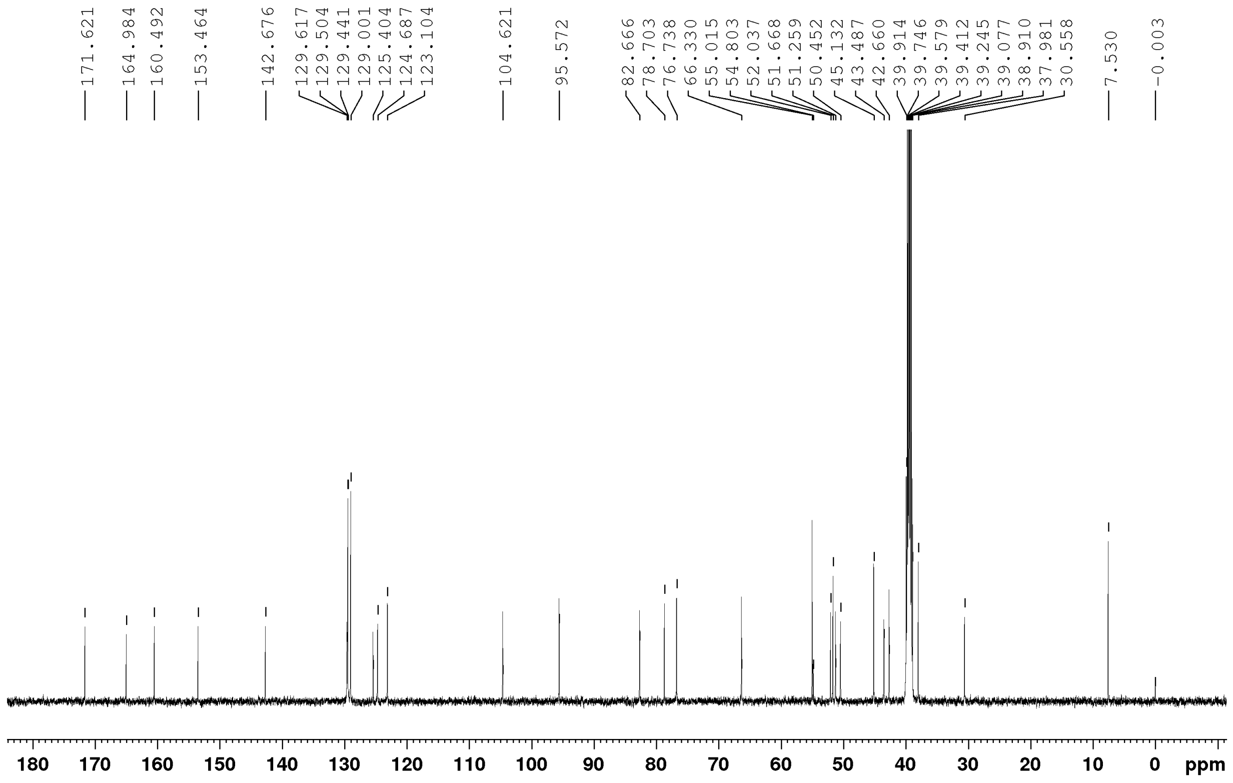

Figure S35. ^{13}C NMR spectrum of compound **11**.

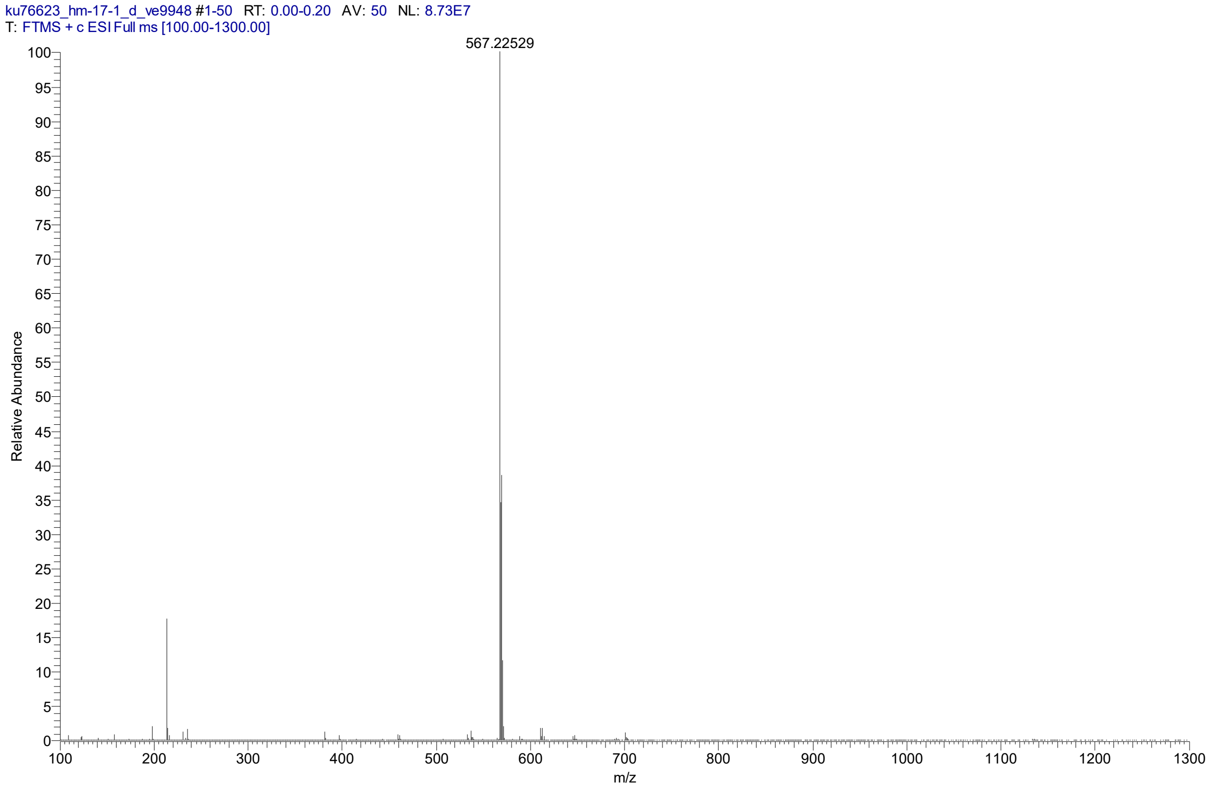

Figure S36. HRMS spectrum of compound **11**.

S1.13. Product 12

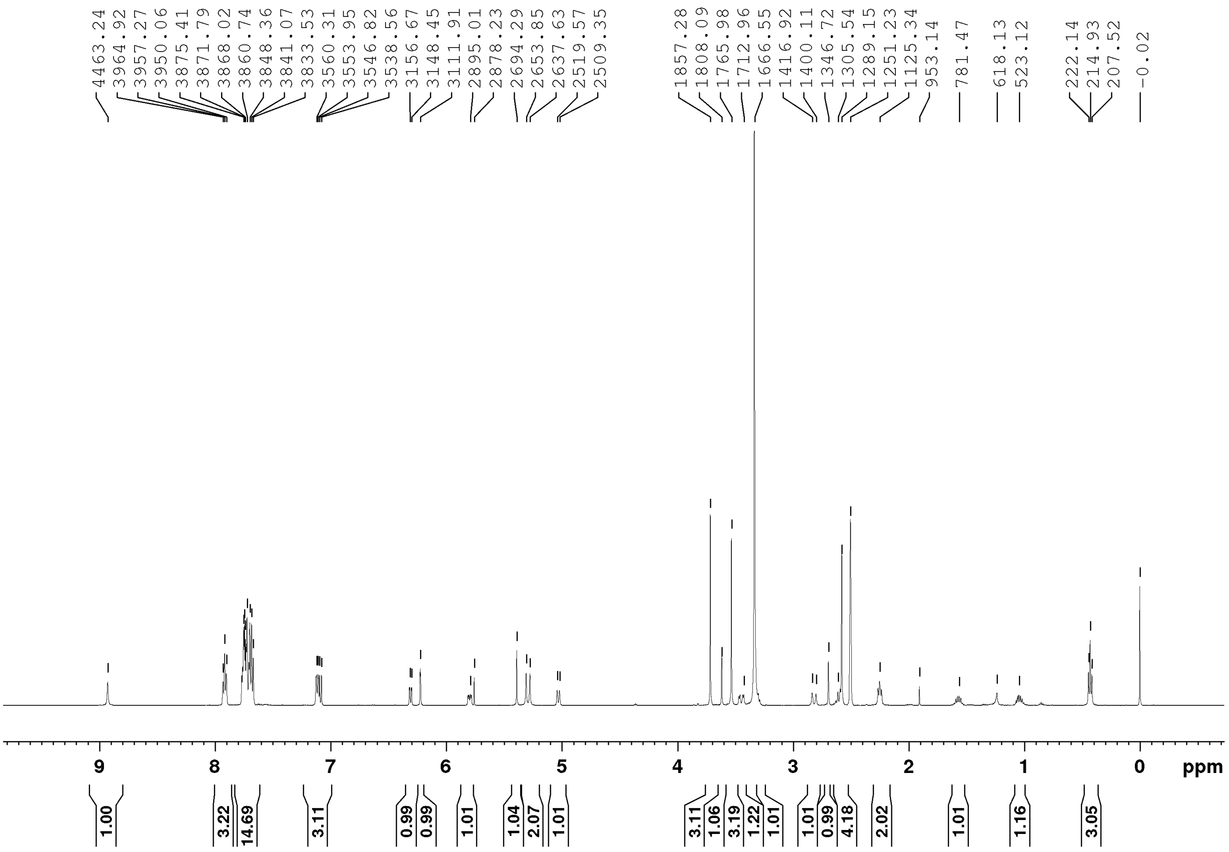

Figure S37. ^{1}H NMR spectrum of compound **12**.

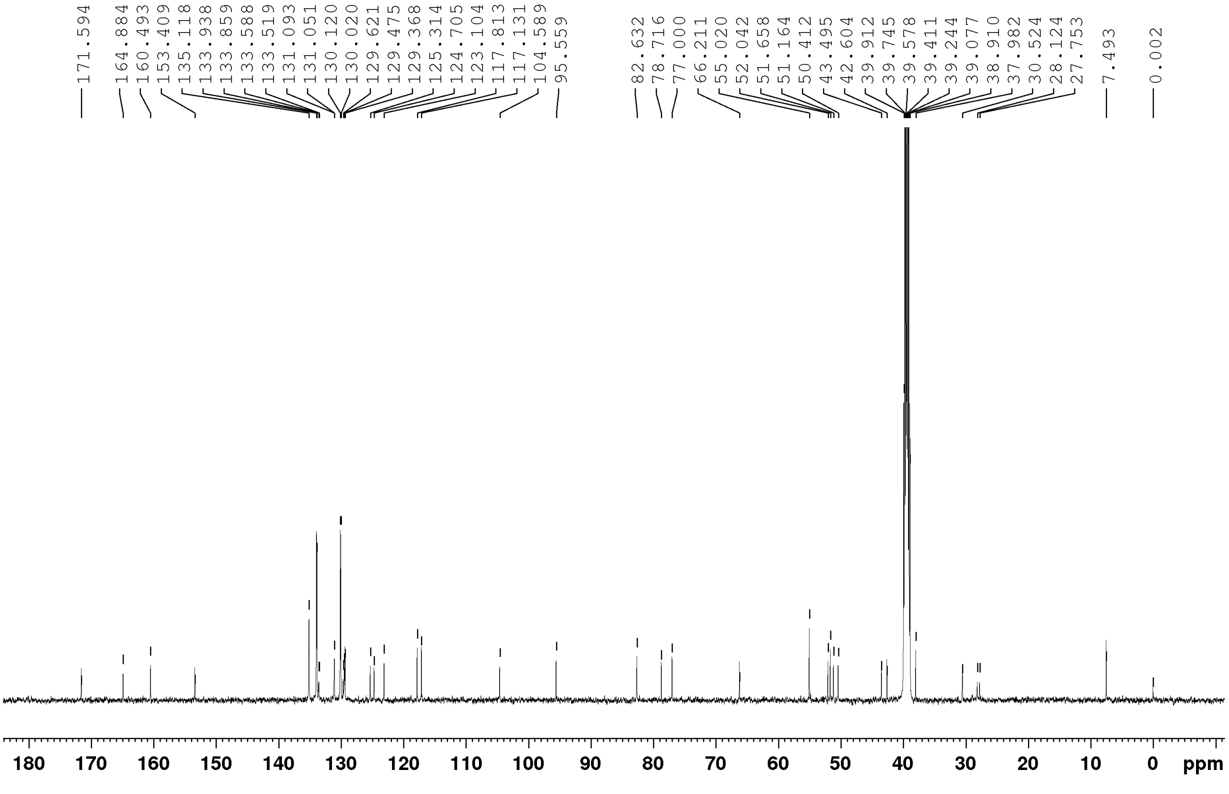

Figure S38. ^{13}C NMR spectrum of compound **12**.

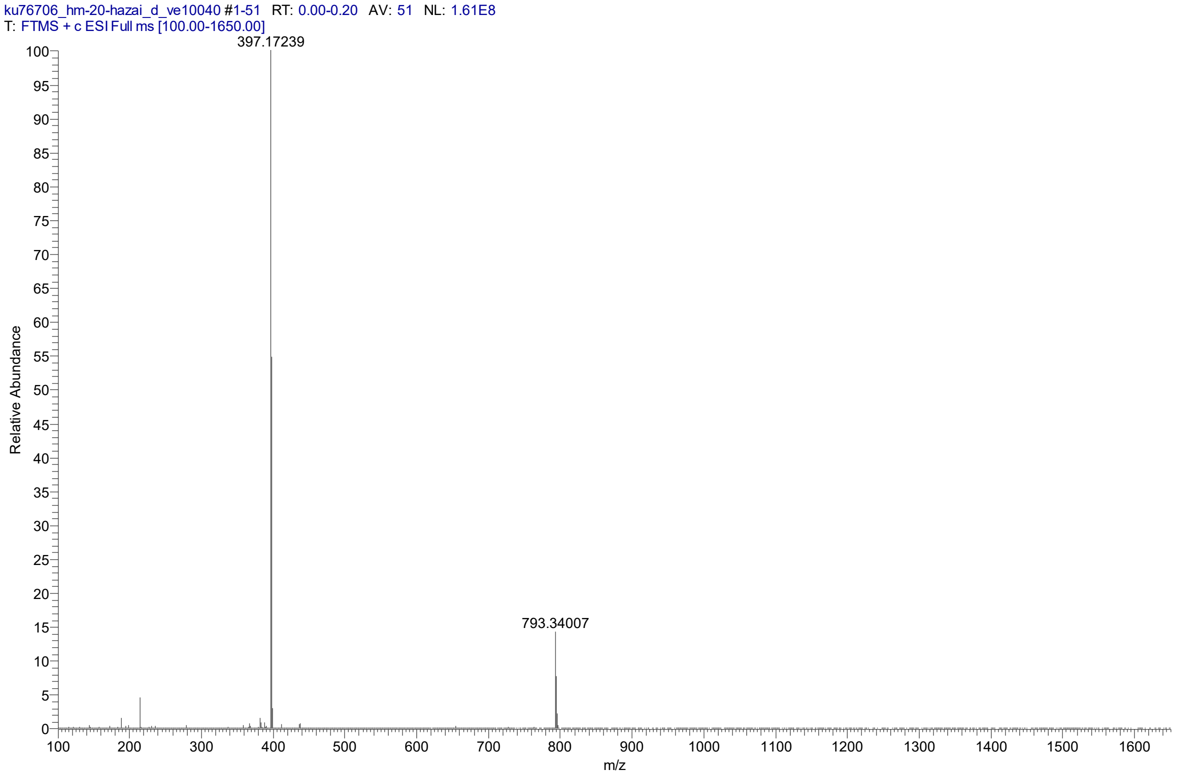

Figure S39. HRMS spectrum of compound **12**.

S1.14. Product 13

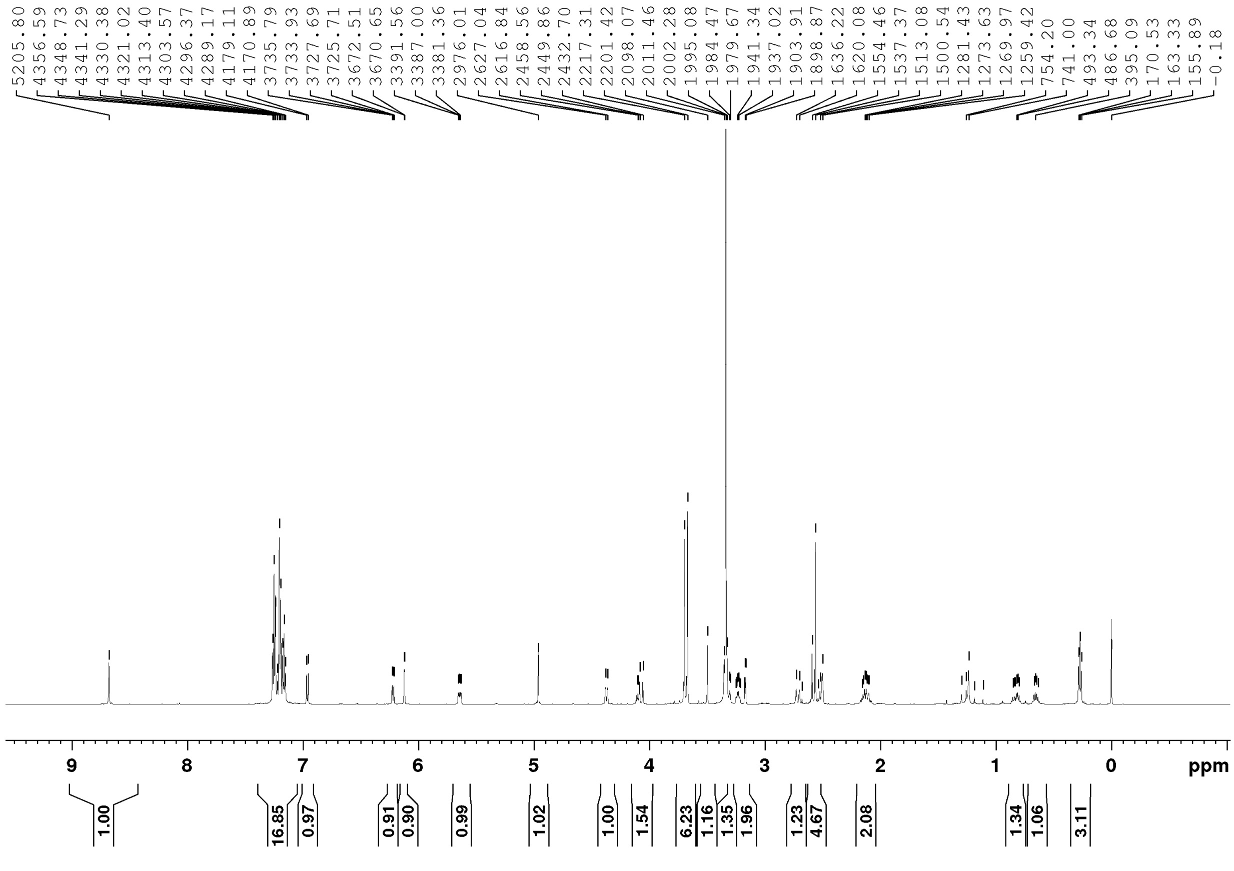

Figure S40. ^{1}H NMR spectrum of compound **13**.

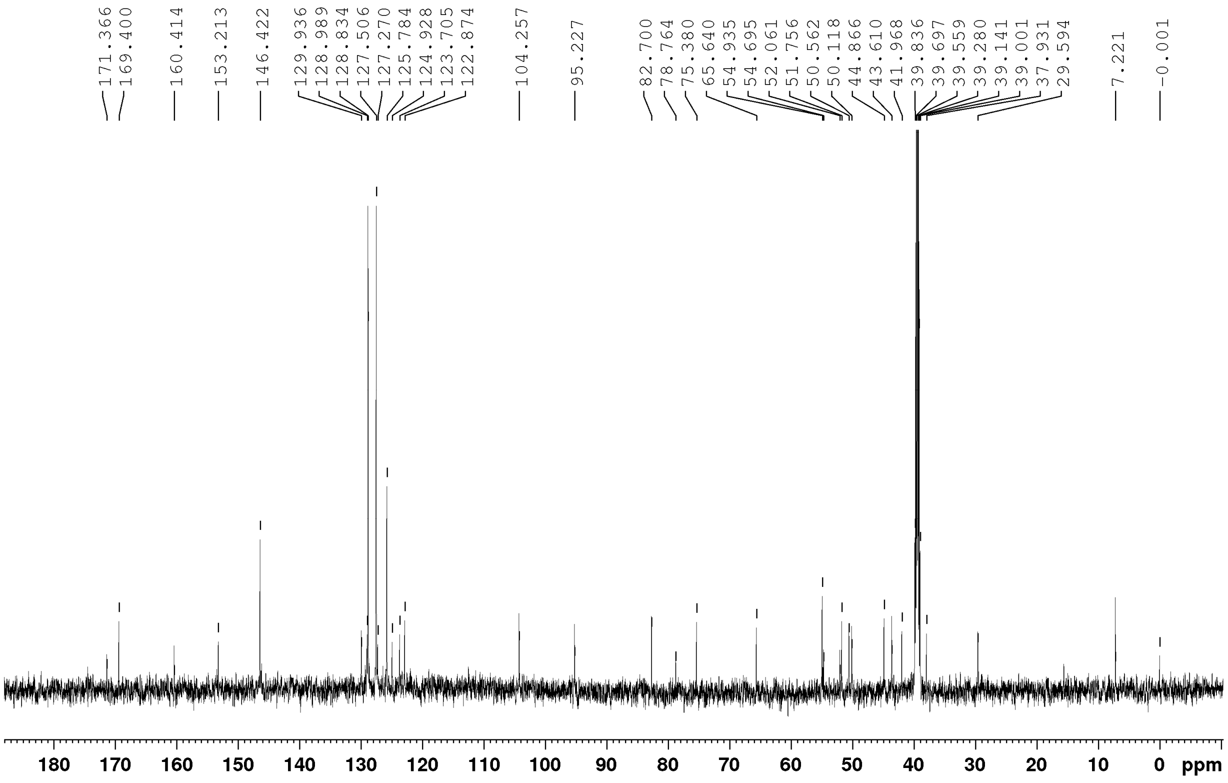

Figure S41. ^{13}C NMR spectrum of compound **13**.

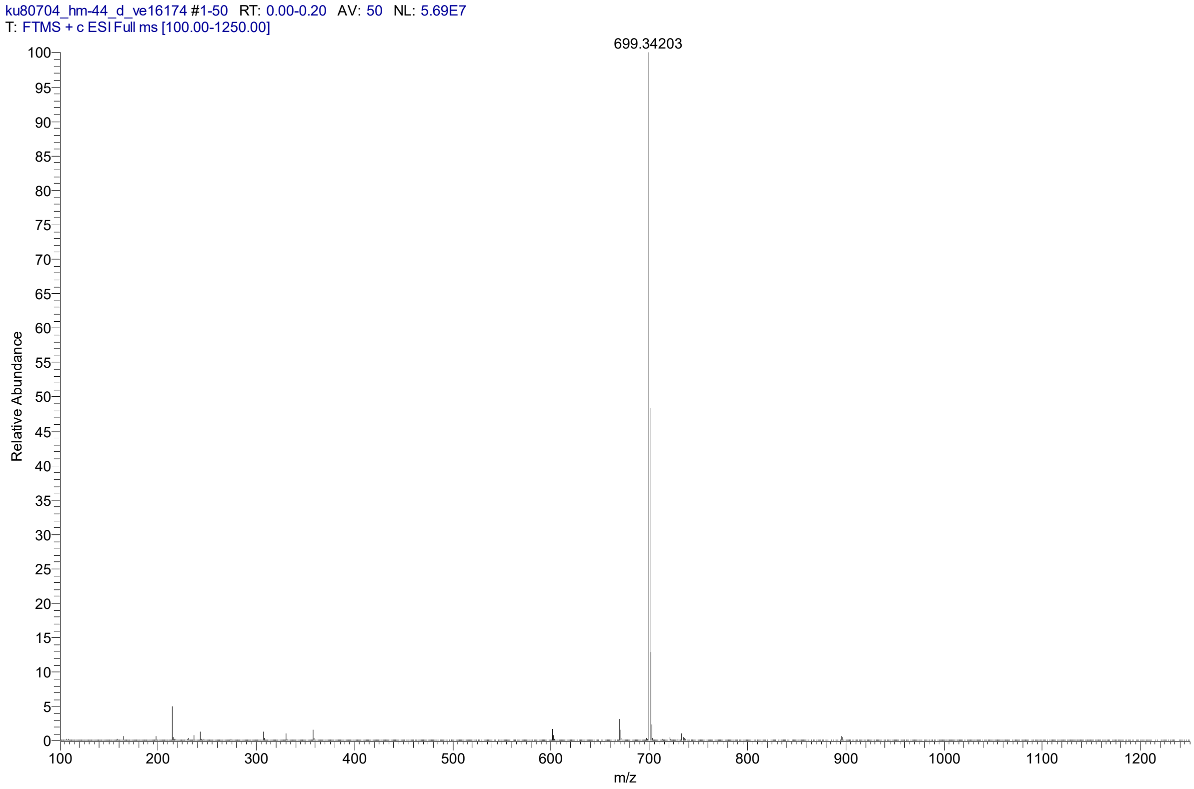

Figure S42. HRMS spectrum of compound **13**.

S1.15. Product 16

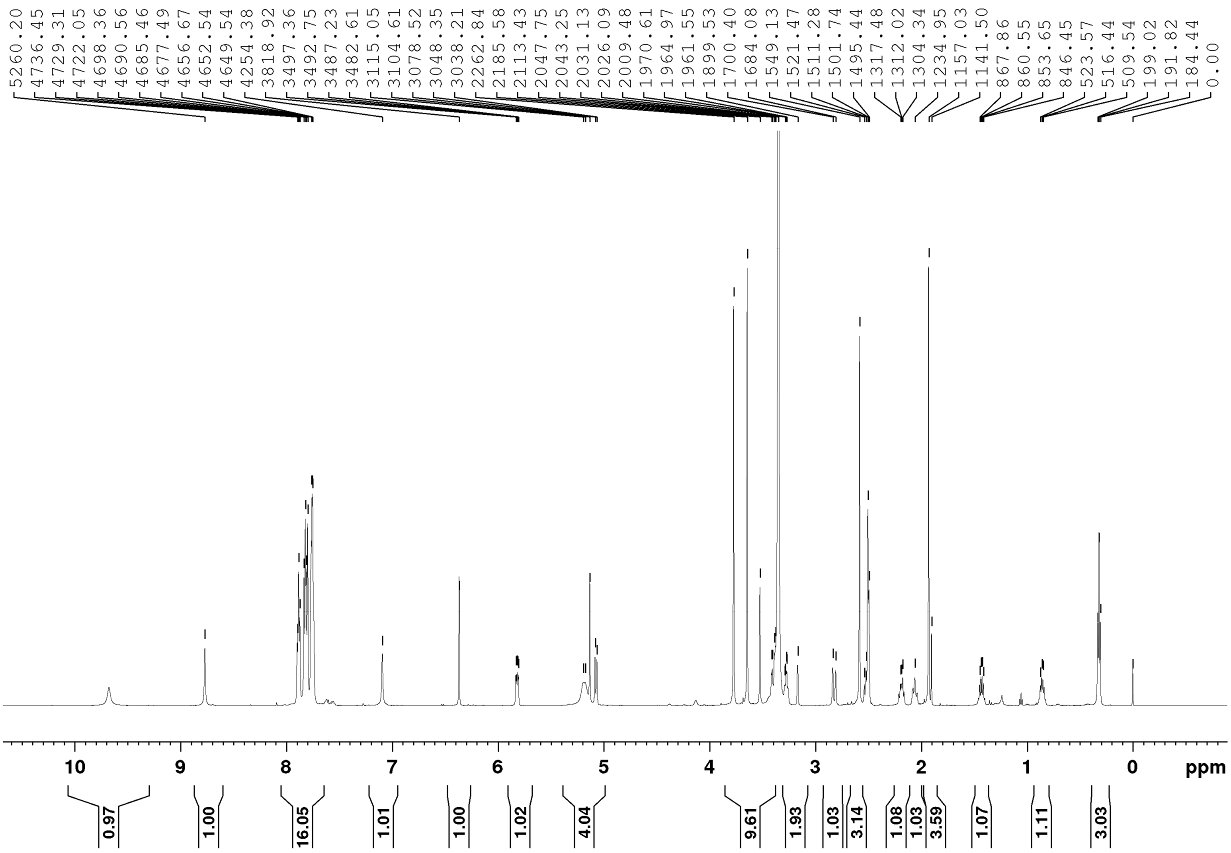

Figure S43. ^{1}H NMR spectrum of compound **16**.

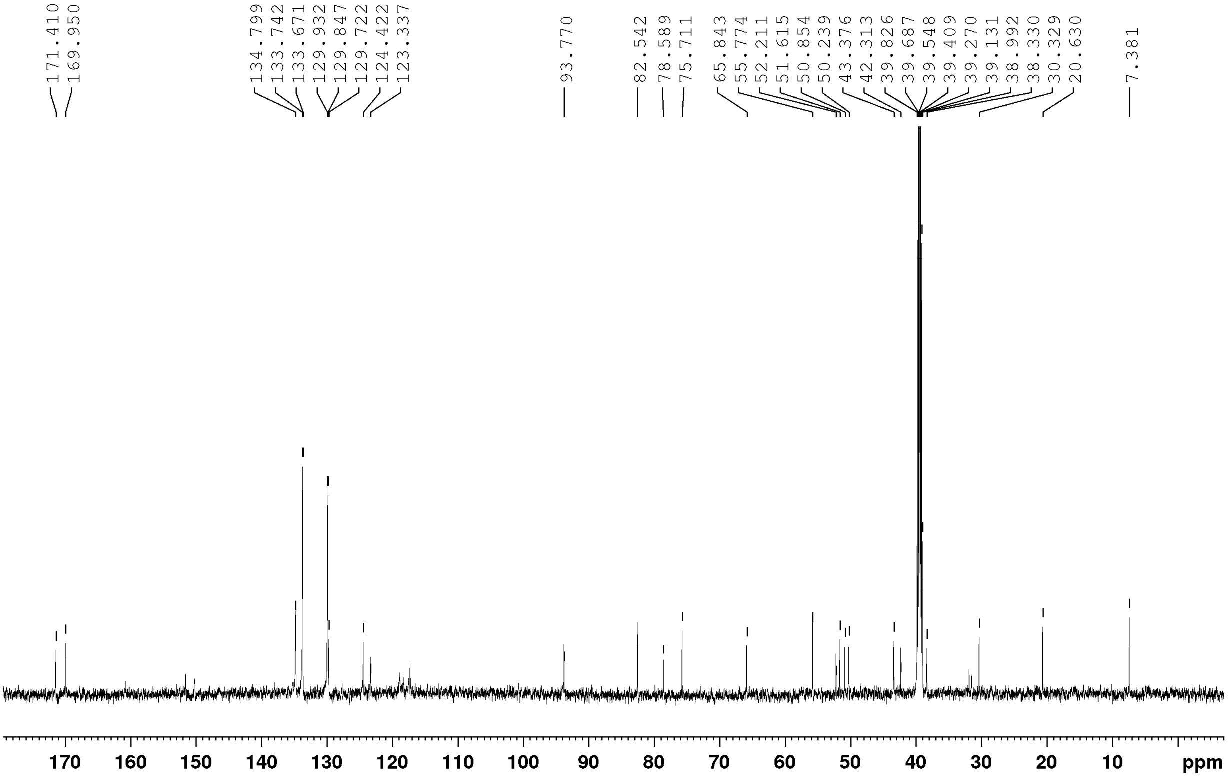

Figure S44. ^{13}C NMR spectrum of compound **16**.

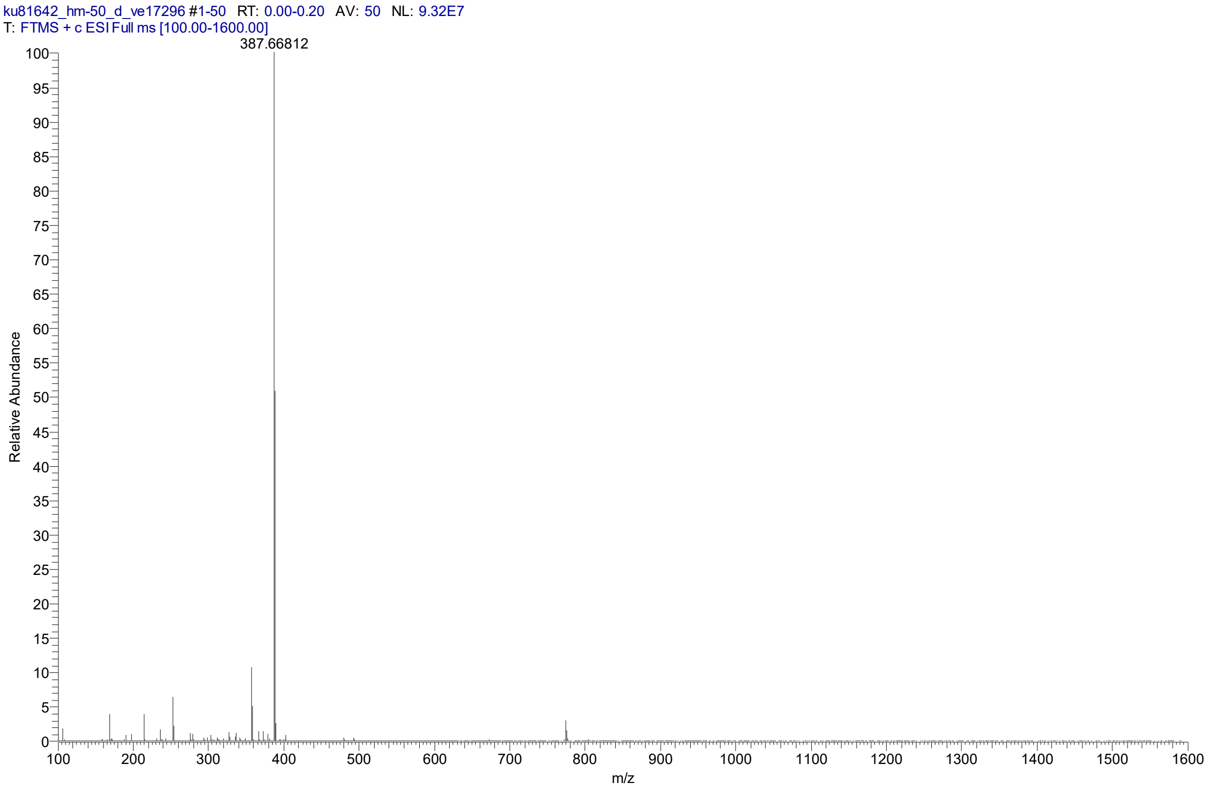

Figure S45. HRMS spectrum of compound **16**.

S2. NCI60 Screening Results

Table S1. Growth percent rates of vindoline (**2**), and compounds **3** and **9a–f** at the concentration of 10 μM against 60 human cancer cell lines *in vitro*. All data with greater than 70% loss of cells are highlighted in bold.

	**2**	**3**	**9a**	**9b**	**9c**	**9d**	**9e**	**9f**
Leukemia								
CCRF-CEM	96.54	11.15	-67.04	**-71.17**	20.01	-40.89	**-95.69**	0.92
HL-60(TB)	103.96	-53.10	-47.53	-39.65	-22.9	-37.12	**-97.22**	-5.95
K-562	103.40	10.42	-13.10	-1.75	21.64	3.31	**-97.52**	-43.4
MOLT-4	103.19	5.41	-45.10	-36.75	37.12	2.35	**-98.31**	21.16
RPMI-8226	100.94	-12.65	-56.31	-45.53	24.38	-43.45	**-98.26**	-38.01
SR	n.d.	9.57	5.74	14.97	42.57	29.14	n.d.	-7.49
Non-small Cell Lung Cancer								
A549/ATCC	93.52	13.44	**-90.26**	**-91.38**	54.49	14.14	**-92.90**	20.13
EKVX	101.77	10.28	**-94.72**	**-85.02**	58.06	15.41	**-98.15**	-8.21
HOP-62	92.23	2.56	**-81.62**	**-81.55**	64.04	4.26	**-97.87**	-13.67
HOP-92	84.21	-40.05	**-83.21**	**-82.89**	-6.20	-43.78	**-98.86**	-58.43
NCI-H226	104.40	13.97	**-77.37**	**-80.30**	46.94	-20.02	**-99.32**	-38.58
NCI-H23	103.04	-8.66	**-90.67**	**-92.48**	35.51	-4.73	**-98.09**	-57.83
NCI-H322M	97.79	35.62	**-98.44**	**-97.68**	65.15	24.47	**-98.13**	25.89
NCI-H460	104.23	6.11	**-85.15**	**-85.23**	30.91	4.64	**-89.49**	-26.85
NCI-H522	88.55	-35.39	**-86.32**	**-89.42**	-17.67	**-82.23**	**-98.40**	**-70.42**
Colon Cancer								
COLO 205	n.d.	**-87.91**	-50.22	-64.39	-18.95	-63.57	**-96.58**	**-90.68**
HCC-2998	117.70	-22.48	**-91.41**	**-89.71**	22.44	**-92.64**	**-97.93**	**-93.07**
HCT-116	104.47	8.72	**-93.37**	-68.79	32.19	0.16	**-93.03**	-51.02
HCT-15	99.02	101.12	**-91.00**	**-86.30**	99.58	76.63	**-93.91**	30.66
HT29	98.55	2.42	**-80.68**	**-84.29**	15.68	-56.71	**-97.47**	-57.82
KM12	102.15	-27.25	**-76.07**	-65.27	21.33	-63.36	**-94.96**	-93.40
SW-620	103.73	17.42	**-85.17**	**-82.38**	45.29	3.91	**-95.83**	-48.69
CNS Cancer								
SF-268	99.98	19.20	-46.26	-34.32	42.32	-54.07	**-97.64**	**-76.11**
SF-295	99.51	23.30	**-89.07**	**-93.70**	84.98	-13.55	**-98.61**	-42.29
SF-539	103.67	16.24	**-96.61**	**-98.88**	24.22	**-86.51**	**-97.25**	**-93.79**
SNB-19	96.60	11.03	**-97.32**	**-96.70**	13.78	-60.00	**-97.12**	**-95.17**
SNB-75	73.08	-4.39	-61.24	-34.02	39.92	-61.44	**-98.91**	**-79.5**
U251	99.44	4.03	**-91.24**	**-93.70**	18.53	**-82.47**	**-96.50**	**-96.9**
Melanoma								
LOX IMVI	99.36	8.81	**-89.35**	**-96.37**	31.05	**-96.00**	**-96.75**	**-95.65**
MALME-3M	97.96	-12.44	**-94.44**	**-97.80**	-32.64	**-96.13**	**-98.40**	**-96.72**
M14	103.84	17.70	**-85.31**	**-88.41**	21.45	**-78.03**	**-97.26**	**-82.93**
MDA-MB-435	105.29	-21.61	-57.48	**-76.90**	6.69	**-80.14**	**-98.17**	**-73.71**
SK-MEL-2	106.74	-65.31	**-85.39**	**-90.56**	-46.87	**-84.88**	**-98.20**	**-97.95**
SK-MEL-28	105.79	20.90	**-96.61**	**-96.29**	-24.10	**-85.89**	**-98.24**	-63.30
SK-MEL-5	102.00	**-91.47**	**-99.44**	**-99.60**	-38.26	**-98.41**	**-97.53**	**-98.38**
UACC-257	98.69	-27.34	**-92.68**	**-93.54**	-34.31	**-80.93**	**-99.30**	**-73.02**
UACC-62	94.73	-59.27	**-96.83**	**-97.13**	-59.52	**-97.32**	**-97.67**	**-73.20**
Ovarian Cancer								
IGROV1	98.42	-0.06	**-89.51**	**-92.12**	-14.96	-85.95	**-98.99**	-43.20
OVCAR-3	112.39	-2.49	-61.89	**-73.60**	21.10	-48.64	**-99.04**	**-75.02**
OVCAR-4	98.82	-3.58	**-73.54**	**-75.27**	48.59	17.74	**-98.66**	-11.05
OVCAR-5	91.21	2139	**-95.94**	**-96.47**	66.96	11.03	n.d.	10.55
OVCAR-8	99.84	3.13	**-84.96**	**-86.23**	28.03	1.19	**-95.46**	6.90
NCI/ADR-RES	110.49	100.23	18.08	79.92	104.37	99.97	**-87.16**	75.94
SK-OV-3	107.57	-21.03	**-81.00**	**-85.14**	54.67	-7.72	**-97.66**	-33.38
Renal Cancer								
786-0	104.33	20.65	**-88.02**	**-89.50**	80.94	-16.25	**-97.92**	**-74.07**
A498	99.59	123.66	**-88.56**	**-83.19**	101.25	21.80	**-98.93**	-10.38
ACHN	101.46	89.87	**-97.78**	**-95.80**	101.43	31.99	**-96.46**	0.60
CAKI-1	97.50	52.20	**-95.32**	**-93.35**	73.08	-8.49	**-97.02**	-40.17
RXF 393	103.81	28.86	**-82.84**	**-92.64**	-10.40	**-70.31**	**-98.62**	**-83.64**
SN12C	96.08	2.73	**-88.10**	**-93.39**	16.58	-40.49	**-96.70**	**-80.64**
TK-10	92.13	22.30	**-88.52**	**-90.65**	63.94	-48.59	**-99.31**	**-88.47**
UO-31	83.93	80.01	**-97.38**	-56.81	81.51	76.82	**n.d.**	44.01
Prostate Cancer								
PC-3	88.31	2.13	**-83.31**	**-78.80**	16.68	-11.98	**-97.70**	4.74
DU-145	107.34	17.99	**-78.50**	**-75.43**	47.69	15.71	**-96.75**	1.94
Breast Cancer								
MCF7	99.03	-3.34	**-76.26**	**-83.04**	7.70	**-72.23**	**-94.55**	-68.14
MDA-MB-231/ATCC	97.65	1.29	**-94.85**	**-96.97**	22.00	**-90.79**	n.d.	**-94.48**
HS 578T	113.32	25.53	-60.26	-65.44	19.11	-33.03	**-98.20**	-47.44
BT-549	114.27	11.04	**-96.88**	**-96.61**	-9.40	**-97.37**	**-97.99**	-64.17
T-47D	95.68	1.08	**-78.43**	-61.39	10.44	-1.81	n.d.	-25.02
MDA-MB-468	101.69	**-75.59**	**-92.14**	**-92.11**	**-74.58**	**-79.60**	**-99.39**	**-84.60**
Mean								
	100.08	3.13	**-78.57**	**-76.15**	26.26	-32.71	**-97.13**	-45.38

Table S2. Growth percent rates of vindoline (**2**), and compounds **4**, **10a–d**, and **12** at the concentration of 10 μM against 60 human cancer cell lines *in vitro*. All data with greater than 70% loss of cells are highlighted in bold.

	**2**	**4**	**10a**	**10b**	**10c**	**10d**	**12**
Leukemia							
CCRF-CEM	96.54	15.57	**-72.4**	-59.09	19.91	-60.23	-46.43
HL-60(TB)	103.96	-63.51	-42.19	-45.16	-32.16	-36.70	-69.05
K-562	103.40	21.66	0.80	1.01	17.11	7.48	-54.90
MOLT-4	103.19	5.66	-24.98	-26.23	29.14	-5.16	-53.05
RPMI-8226	100.94	15.42	-46.00	-68.85	4.14	-44.98	-50.80
SR	n.d.	17.89	25.79	23.23	28.76	19.47	-58.32
Non-small Cell Lung Cancer							
A549/ATCC	93.52	22.07	**-92.2**	**-83.68**	37.55	11.65	30.99
EKVX	101.77	56.10	**-93.84**	-59.67	52.96	13.17	28.74
HOP-62	92.23	26.42	**-84.03**	**-80.79**	50.95	9.52	-32.70
HOP-92	84.21	-30.04	**-77.92**	**-74.51**	-12.2	-44.50	**-73.39**
NCI-H226	104.40	15.10	**-78.61**	-63.57	38.05	-22.04	-23.12
NCI-H23	103.04	6.40	**-92.39**	**-90.74**	23.57	-9.66	0.48
NCI-H322M	97.79	47.05	**-97.58**	**-89.65**	52.89	16.06	19.95
NCI-H460	104.23	18.44	**-86.62**	**-86.99**	20.28	5.27	-62.82
NCI-H522	88.55	-5.50	**-82.65**	**-81.92**	-11.64	**-71.81**	-17.40
Colon Cancer							
COLO 205	n.d.	**-93.73**	-64.14	-64.93	-43.39	**-76.57**	**-87.23**
HCC-2998	117.70	15.90	**-85.78**	**-88.44**	19.44	**-87.80**	**-74.56**
HCT-116	104.47	8.03	**-93.50**	**-94.93**	23.42	4.69	-62.77
HCT-15	99.02	99.26	**-90.08**	-5.69	101.97	75.16	90.07
HT29	98.55	2.65	**-81.77**	-63.14	15.94	-23.96	-39.40
KM12	102.15	3.15	**-73.59**	-68.76	16.03	**-71.07**	-65.80
SW-620	103.73	29.36	**-88.15**	**-88.66**	36.85	2.08	-65.97
CNS Cancer							
SF-268	99.98	26.23	-61.08	-38.08	28.06	**-70.52**	-26.12
SF-295	99.51	58.15	**-91.82**	**-93.61**	78.45	-19.65	5.90
SF-539	103.67	19.02	**-98.66**	**-97.45**	16.02	**-93.90**	**-90.56**
SNB-19	96.60	12.74	**-96.48**	**-95.70**	6.61	**-71.11**	**-72.21**
SNB-75	73.08	-12.82	**-87.64**	**-95.38**	-5.30	**-81.40**	-61.72
U251	99.44	10.93	**-93.59**	**-95.94**	13.02	**-87.89**	**-72.61**
Melanoma							
LOX IMVI	99.36	18.51	**-94.51**	**-96.37**	25.00	**-96.11**	-39.75
MALME-3M	97.96	-35.81	**-95.99**	**-97.16**	-55.92	**-95.92**	**-95.48**
M14	103.84	29.40	**-88.34**	**-89.84**	19.73	**-83.49**	**-73.88**
MDA-MB-435	105.29	-19.67	**-86.76**	**-88.53**	-16.01	**-90.50**	**-88.78**
SK-MEL-2	106.74	-41.58	**-87.25**	**-91.43**	-54.02	**-87.99**	**-77.77**
SK-MEL-28	105.79	12.46	**-97.80**	**-96.85**	-54.87	**-86.50**	**-92.36**
SK-MEL-5	102.00	**-72.86**	**-98.50**	**-97.04**	-59.65	**-96.15**	**-98.50**
UACC-257	98.69	2.15	**-95.01**	**-93.44**	-50.70	**-82.11**	-67.57
UACC-62	94.73	-6.54	**-97.42**	**-95.2**	-69.63	**-96.90**	**-86.94**
Ovarian Cancer							
IGROV1	98.42	-36.41	**-91.80**	**-92.04**	-42.77	**-84.72**	-69.61
OVCAR-3	112.39	-0.29	**-77.10**	**-71.77**	12.56	-60.26	-63.37
OVCAR-4	98.82	5.22	-68.86	-37.70	33.09	12.45	7.65
OVCAR-5	91.21	23.79	**-96.02**	**-98.57**	49.71	5.84	2.52
OVCAR-8	99.84	18.59	**-86.59**	**-90.20**	19.99	-6.33	-18.47
NCI/ADR-RES	110.49	91.61	71.78	84.27	105.46	96.67	-
SK-OV-3	107.57	-2.96	**-82.26**	**-82.55**	41.37	-22.25	-13.15
Renal Cancer							
786-0	104.33	57.21	**-90.97**	**-92.87**	87.05	-36.02	-47.81
A498	99.59	52.19	**-90.92**	-54.72	99.22	-0.04	70.23
ACHN	101.46	93.05	**-99.27**	-49.25	102.51	18.73	73.72
CAKI-1	97.50	85.87	**-95.23**	**-78.52**	73.25	-24.15	-7.04
RXF 393	103.81	-13.31	**-87.60**	**-90.41**	-9.93	**-73.75**	-61.83
SN12C	96.08	4.56	**-88.54**	**-95.29**	10.61	-57.94	-46.47
TK-10	92.13	48.62	**-91.25**	**-88.20**	70.23	-64.11	21.40
UO-31	83.93	81.87	**-96.04**	15.50	85.90	78.86	88.30
Prostate Cancer							
PC-3	88.31	n.d.	**-73.25**	-37.25	15.74	3.69	-21.85
DU-145	107.34	14.71	**-84.54**	-58.22	36.42	9.57	16.90
Breast Cancer							
MCF7	99.03	4.69	**-81.19**	**-82.60**	5.18	-64.15	-52.8
MDA-MB-231/ATCC	97.65	-2.41	**-97.21**	**-97.40**	2.85	**-92.80**	**-91.52**
HS 578T	113.32	9.79	-69.88	-67.45	-2.23	-34.95	-36.57
BT-549	114.27	-1.04	**-97.04**	**-96.65**	-23.54	**-97.39**	**-89.11**
T-47D	95.68	4.99	-64.53	-46.62	12.80	3.83	7.10
MDA-MB-468	101.69	-63.92	**-92.46**	**-92.71**	**-74.63**	**-78.98**	-49.163
Mean							
	100.08	11.87	**-78.72**	**-70.41**	17.02	-36.64	-37.12

S3. NCI60 Screening

A detailed description of the NCI screening procedures [43-47] can also be found on the website of NCI [48], and in our previous work [40,42].

S3.1. One-Dose Screen

All candidates were examined at first at a single high dose (10 μ*M*) in the full NCI60 cell panel [43-48]. The value reported for the one-dose test is growth relative to the no-drug control and relative to the time zero number of cells. This made possible the disclosure of both growth inhibition (numbers between 0 and 100) and lethality (numbers less than 0). For example, a value of 100 means no growth inhibition. A value of 10 would mean 90% growth inhibition. A value of 0 means no net growth over the course of the analysis. A value of −10 would mean 10% lethality. A value of −100 means all cells are dead.

S3.2. Five-Dose Screen

Candidates that showed remarkable growth inhibition in the one-dose assay were subjected to the 60-cell panel at five concentration levels. The human tumor cell lines of the cancer screening panel were grown in RPMI 1640 medium containing 5% fetal bovine serum and 2 m*M* l-glutamine. Characteristically, cells were injected in 96-well microtiter plates in 0.1 mL at plating densities ranging from 5000 to 40,000 cells/well, depending on the doubling time of individual cell lines. After cell inoculation, the microtiter plates were incubated at 37 °C, 5% carbon dioxide, 95% air, and 100% relative humidity for 24 hours before the addition of tested compounds. After 24 hours, 2 plates of each cell line were fixed *in situ* with Cl_3COOH (TCA), to represent a measurement of the cell population for each cell line at the time of drug addition (t_z). Tested compounds were solubilized in DMSO at 400-fold the desired final maximum test concentration and stored frozen before application. In the course of the drug addition, an aliquot of frozen concentrate was thawed and diluted to twice the desired final maximum test concentration with a complete medium containing 50 μg mL^{-1} gentamicin. Additional four, 10-fold or ½ log serial dilutions were produced to furnish a total of five drug concentrations plus control. Aliquots of 0.1 mL of these different drug dilutions were added to the appropriate microtiter wells already containing 0.1 mL of medium, resulting in the required final drug concentrations.

Following drug addition, the plates were incubated at 37 °C, 5% carbon dioxide, 95% air, and 100% relative humidity for an additional 48 hours. For adherent cells, the test was finished by the addition of cold Cl_3COOH. Cells were fixed *in situ* by the addition of 50 μL of cold 50% (w/v) Cl_3COOH, and incubated at 4 °C for 1 hour. The supernatant was discarded, and the plates were washed with H_2O (5×) and dried in air. Sulforhodamine B (SRB) solution (0.1 mL) at 0.4% (w/v) in 1% CH_3COOH was added to each well, and plates were incubated at RT for 10 min. After staining, the unbound dye was removed by washing five times with 1% CH_3COOH, and the plates were dried in the air. The bound stain is afterward solubilized with a 10 mM trizma base, and the absorbance is read on an automated plate reader at $\lambda = 515$ nm. Utilizing the seven absorbance measurements [time zero (t_z), control growth (c), and test growth in the presence of the drug at the five concentration levels (t_i)], the percentage growth was determined at each of the drug concentration levels. Growth inhibition (%) was calculated as:

$$[(t_i - t_z)/(c - t_z)] \times 100, \text{ for concentrations where } t_i \geq t_z \quad \text{(S1)}$$

$$[(t_i - t_z)/(t_z)] \times 100, \text{ for concentrations where } t_i < t_z. \quad \text{(S2)}$$

Three dose–response parameters were calculated as follows. GI_{50} (growth inhibition of 50%) was determined from Equation (S3), which is the drug concentration resulting in a 50% reduction in the net protein increase (as measured by SRB staining) in control cells in the course of the drug incubation.

$$[(t_i - t_z)/(c - t_z)] \times 100 = 50 \quad \text{(S3)}$$
